# Update of guideline for diagnosis and treatment of subacromial pain syndrome: a multidisciplinary review by the Dutch Orthopedic Association: Part 1: preventive measures, diagnostics, and non-surgical treatment of subacromial pain syndrome

**DOI:** 10.2340/17453674.2026.45365

**Published:** 2026-02-16

**Authors:** Frederik O LAMBERS HEERSPINK, Egbert J D VEEN, Oscar DORRESTIJN, Cornelis P J VISSER, Maarten J C LEIJS, Dennis VAN POPPEL, Peter A STROOMBERG, Ramon P G OTTENHEIJM, Jan W KALLEWAARD, Tjerk J W DE RUITER, Henk A MARTENS, Femke M JANSSEN, Tessa GELTINK, Matthijs S RUITER, Jos J A M VAN RAAIJ

**Affiliations:** 1 Department of Orthopaedics, Viecuri Medical Center, Venlo; 2 Department of Orthopaedics and Research School Caphri, Maastricht University Medical Centre+, Maastricht; 3 Department of Orthopaedics, Medical Spectrum Twente, Enschede; 4 Department of Orthopaedics, Sint Maartens Clinic, Nijmegen; 5 Department of Orthopaedics, Alrijne and Eisenhower Clinic, Leiderdorp; 6 Department of Orthopaedics, Reinier Haga Orthopedic Center, Zoetermeer; 7 Department of Physical Therapy, PECE Zorg, Fontys, Eindhoven; 8 Department of Radiology, Isala clinic, Zwolle; 9 Department of General Practice, Maastricht University, Maastricht; 10 Department of Anesthesiology, Rijnstate Clinic, Arnhem; 11 Department of Rehabilitation, De Ruiter Rehabilitation, Hengelo; 12 Department of Rheumatology, Sint Maartens Clinic, Nijmegen, the Netherlands; 13 Knowledge Institute of the Federation of Medical Specialists, advisor, Utrecht; 14 Department of Orthopaedics, Martini Hospital Groningen, Groningen, the Netherlands

## Abstract

**Background and purpose:**

In 2013, the first clinical practice guideline for subacromial pain syndrome (SAPS) was developed in the Netherlands to support healthcare professionals. SAPS refers to non-traumatic, non-rheumatologic shoulder complaints that are particularly painful during arm elevation. It includes conditions such as supraspinatus tendinosis, calcific tendinitis, and degenerative supraspinatus tears. Over 50,000 patients annually consult orthopedic surgeons for these issues. In response to new evidence and clinical needs, an updated guideline was developed. Part 1 addresses prevention, diagnosis, imaging, and non-surgical treatment. Using a multidisciplinary, evidence-based approach, the guideline aims to answer key clinical questions around SAPS.

**Methods:**

Initiated by the Dutch Orthopedic Society, the guideline committee identified knowledge gaps through group sessions. Each module was based on a PICO-formatted key question and reviewed by professionals from different fields. The AGREE and GRADE methods were applied to ensure a systematic evaluation of evidence, leading to conclusions and recommendations.

**Results:**

(i) Inform patients about the potential positive effects of a healthy lifestyle and encourage gradual exercise within sport and work. (ii) Perform a cluster of physical diagnostic tests to diagnose SAPS. (iii) Perform ultrasonography in patients with clinical suspicion of (partial thickness) rupture of the supraspinatus tendon. Consider MRI if ultrasound is not available or inconclusive. (iv) Consider barbotage for symptomatic calcific tendinosis, preferably with corticosteroid injection in the bursa, if a previous corticosteroid injection was ineffective. (v) Consider a subacromial corticosteroid injection (with a local anesthetic) to enable exercise therapy in patients with severe complaints that impair their ability to participate in exercise therapy. (vi) Consider suprascapular nerve block for patients with therapy-resistant SAPS when other non-surgical treatment is ineffective.

**Conclusion:**

The updated guideline provides multidisciplinary recommendations for physical examination, imaging, and conservative management of SAPS.

Subacromial pain syndrome (SAPS) is a condition treated by various healthcare providers. In the Netherlands, the diagnosis of “supraspinatus tendinitis”, a component of SAPS, is made 50,000 to 60,000 times each year by orthopedic surgeons [1]. In 2013, the Dutch Orthopedic Association released a multidisciplinary guideline for SAPS treatment, advising caution with decompression surgery [2]. In 2021, the module on surgical treatment for SAPS was updated, strongly recommending against subacromial decompression surgery. This recommendation is based on scientific evidence showing that surgery offers no additional benefit over non-surgical treatment [3-5]. Since the guideline’s introduction, national referrals to orthopedic surgeons decreased, resulting in a 37% drop in acromioplasties [1]. This decrease in surgeries underscores the impact of guideline implementation in clinical practice. Recent research has provided new insights into SAPS management, such as treating modalities for calcifying tendinitis, treatment of supraspinatus tears, and long head biceps pathology, prompting the need for further guideline updates [6].

In this article, we describe the process of updating the multidisciplinary evidence-based guideline to facilitate clinical decision-making, diagnosis, and treatment of SAPS for all healthcare professionals involved in treating patients with SAPS in the setting of secondary care,, including orthopedic surgeons, anesthesiologists, and radiologists. Moreover, this guideline aligns with the guidelines for general practitioners and primary care physiotherapists, making it relevant not only for these professionals but also for others such as rehabilitation physicians, rheumatologists, social and insurance physicians, and occupational physicians. For clarity, the guideline is divided into 2 parts. This part 1 addresses preventive measures and diagnostics in SAPS; furthermore, non-surgical treatment modalities for calcifying tendinitis are compared; subacromial injections and suprascapular nerve blocks (SSNB) are discussed. Part 2 discusses the treatment, prognostic factors, and rehabilitation of isolated degenerative supraspinatus tendon tears and compares surgical treatment for calcifying tendinitis with needle aspiration of the calcific depot [7].

## Methods

### Terminology

As there is no universal definition of SAPS, the guideline working group formulated the following definition for SAPS. Subacromial pain syndrome (SAPS) refers to non-traumatic, non-rheumatological shoulder complaints characterized primarily by pain during arm elevation. It is a condition presenting with a range of pathological features, including supraspinatus tendinosis, calcific tendinitis, and degenerative changes of the rotator cuff, such as degenerative isolated full-thickness supraspinatus tendon tears. Although most studies on SAPS terminology exclude rotator cuff tears, the guideline development group chose to include degenerative isolated full-thickness supraspinatus tears, given the ongoing debate surrounding their optimal management [8]. Although the guideline development group recognizes that atraumatic instability can contribute to secondary SAPS symptoms, this was not addressed in the guideline. Moreover, conditions such as frozen shoulder, isolated long head biceps pathology, glenohumeral and acromioclavicular (AC) osteoarthritis, multi-tendon or irreparable rotator cuff tears, primary intra-articular pathology, and neurological disorders were excluded from the guideline’s scope.

### Guideline working group formation and the formulation of scoping questions

A guideline working group was established in 2022 by order of the Dutch Orthopedic Society to update the guideline from 2013 [2]. The working group included expert representatives from relevant disciplines (orthopedic surgeons, radiologists, anesthesiologists, rheumatologists, general practitioners, rehabilitation and occupational therapists, and physical therapists). This expert group, specialized in shoulder pathology, received methodological support from methodologists of the Knowledge Institute of the Dutch Association of Medical Specialists.

In the preparatory phase, a written invitational conference was held to gather input from relevant stakeholders on clinical questions concerning the optimal care of patients with SAPS. This input has been discussed in the guideline working group and processed into a concept framework of scoping questions. Patient perspective was secured through invitation of the Dutch Patient Federation (Patiëntenfederatie Nederland) to give input into the invitational conference and in the commentary phase.

2 guideline modules (outcome measures for SAPS and patient perspectives on SAPS) were considered up-to-date and were therefore retained without updates [9]. 11 clinical questions were formulated. Table 1 presents the clinical topic with corresponding clinical questions and the outcome measures used. Each clinical question was then elaborated into separate guideline modules. In part 1 we focus on preventive measures and diagnostics in SAPS; furthermore, non-surgical treatment modalities for calcifying tendinitis are compared, and subacromial corticosteroid injections and suprascapular nerve blocks (SSNB) are discussed. Part 2 covers the treatment, prognostic factors, and rehabilitation of isolated degenerative supraspinatus tendon tears and compares surgical treatment for calcifying tendinitis with needle aspiration of the calcific depot [7]. A guideline module consists of a scoping question, background, methods, results of a systematic evidence review, considerations, rationale, and recommendations.

**Table 1:** Scoping questions and outcome measures per module in Part 1 of the guideline

Clinical topic	Scoping questions	Outcomes
1. Preventive measures		
	What preventive measures can be used in the working population to prevent recurrent SAPS?	SAPS complaints (pain), return to work or leisure, healthcare consumption
2. Diagnostic accuracy of physical examination		
	What is the diagnostic accuracy for using a combination of multiple tests compared with a single test in diagnosing or ruling out of SAPS?	Pain, functionality, return to work or leisure, diagnostic test accuracy measures (sensitivity, specificity)
3. Ultrasonography		
	What is the value of ultrasonography vs MRI in patients who are suspected to have a partial thickness tear in the supraspinatus tendon?	Diagnostic accuracy: false negatives (FN), sensitivity, negative predictive value (NPV), false positives (FP), true positives (TP), true negatives (TN), specificity, positive predictive value (PPV)
4. Barbotage compared with shockwave therapy		
	What is the effectiveness of barbotage compared with shockwave in patients with tendinosis calcarea on patient-reported outcome measures?	Pain, PROMs for function (CMS, DASH, WORC, ASES, OSS, DSST), patient satisfaction, complications/adverse events, return to work or leisure
5. Subacromial injection		
	What are the benefits and harms of subacromial cortico-steroid injection in addition to exercise in patients with subacromial shoulder complaints?	Pain, function, complications, recurrence, patient satisfaction, return to work
6. Suprascapular nerve block		
	What is the effectiveness of suprascapularis blockade vs corticosteroid injection in SAPS patients on patient-reported outcome measures?	Pain reduction, function (constant Murley score), quality of life, rehabilitation time, return to work or leisure

SAPS: subacromial pain syndrome; SA: subacromial SA; SSNB: suprascapular nerve block; CMS: Constant-Murley score; DASH: Disabilities of the Arm, Shoulder and Hand; WORC: Western Ontario Rotator Cuff index; ASES: American Shoulder and Elbow Surgeons score; OSS: Oxford Shoulder Score; DSST: Dutch Simple Shoulder Test; PROMs: patient-reported outcome measures.

### Guideline methodology

Scoping questions were converted into PICOs (patient population, intervention, comparison, outcomes). Outcomes were categorized into crucial or important outcome measures for clinical decision-making. Minimally clinically important differences (MCIDs) were identified per outcome measure. For each PICO, a systematic literature search was conducted in multiple relevant databases (see Supplementary data). Study selection was performed by 2 members of the guideline working group and checked by a methodologist.

The supplementary material provides detailed information on the guideline methodology, including the systematic literature search, selection strategy, risk of bias, and GRADE assessment of the separate guideline modules. This guideline was developed in conformance with the Appraisal of Guidelines for Research and Evaluation II (AGREE II) [10], and the Grading of Recommendations, Assessment, Development and Evaluations (GRADE) method was used to rate the certainty of the evidence [11]. Elaboration on the GRADE quality of evidence is provided in Table 2.

**Table 2:** GRADE level of evidence classifications

GRADE quality of evidence	Definition
High	There is high confidence that the true effect of treatment is close to the estimated effect of treatment; it is very unlikely that the literature conclusion will change in a clinically relevant way when results of new large-scale research are added to the literature analysis.
Moderate	There is reasonable assurance that the true effect of treatment is close to the estimated effect of treatment; it is possible that the conclusion will change in a clinically relevant way when the results of new large-scale studies are added to the literature analysis.
Low	There is low certainty that the true effect of treatment is close to the estimated effect of treatment; there is a real chance that the conclusion will change in a clinically relevant way when results of new large‐scale research are added to the literature analysis.
Very low	There is very low certainty that the true effect of treatment is close to the estimated effect of treatment; the literature conclusion is very uncertain.

GRADE: Grading of Recommendations, Assessment, Development and Evaluation.

### From evidence to recommendation

The strength of the evidence and considerations together form the foundation on which the strength of the recommendation is determined. A low quality of evidence does not exclude the possibility of issuing a strong recommendation. In line with the Evidence-to-Decision framework, the considerations section of the guideline module addresses additional factors such as patient values and preferences, financial considerations, resource use, and feasibility. The recommendations are grounded in an assessment of the balance between positive and negative effects. Potential prerequisites and barriers to implementation were identified, and corresponding action items with assigned responsibilities were outlined to support optimal implementation of the recommendations.

### Consensus process

Consensus on recommendations was obtained through group meetings (digital and in person) in which all guideline working group members had an equal voice. After the development phase, the concept guideline was distributed amongst all relevant stakeholders for commentary. After processing of the comments, the final version of the guideline was again distributed amongst relevant stakeholders for authorization.

### Funding, use of AI, and disclosures

The Guideline update was financed by the Quality Fund for Medical Specialists (Stichting Kwaliteitsgelden Medisch Specialisten, SKMS), which is a quality fund for medical specialists in the Netherlands. The financier has had no influence on the content of the guideline modules.

No generative artificial intelligence tools were used in the preparation of this manuscript.

Complete disclosure of interest forms according to ICMJE are available on the article page, doi: 10.2340/17453674.2026.45365

## Results

### Scoping question 1: What preventive measures can be used in the working population to prevent recurrent SAPS?

A systematic search identified 683 articles, but none met the selection criteria after title and abstract screening. Therefore, the recommendations are based on literature on (tendon) recovery, clinical reasoning, biomechanical insights, expert opinions of occupational disability specialists, and company doctors. Central to preventing (recurrent) SAPS is behavior change, which should ideally encompass both lifestyle and work domains.

***Recommendations regarding lifestyle:***

- Inform patients about the potential positive effects of a healthy lifestyle.
- Consider advising caution with prolonged NSAID use and limiting frequent corticosteroid injections. These should be considered as a facilitator to support physical therapy (see Question 5), rather than as a standalone treatment.
- Encourage gradual exercise within sports and work to increase the load-bearing capacity of the cuff.

***Recommendations regarding work:***

- Advise patients to move within their comfort zone and avoid activities that are too long, heavy, or frequent.
- Limit movements such as reaching, forward flexion, and abduction above shoulder level, as well as internal and external rotation to the limits of the ROM.
- Optimize workspace layout, take regular breaks, and use assistive devices.

### Clinical question 2: What is the diagnostic accuracy for using a combination of multiple tests compared with a single test in diagnosing or ruling out SAPS?

A systematic literature search resulted in 629 hits. 15 studies were initially selected based on title and abstract screening. After full text review, 2 studies were included [12,13]. These studies focused on different categories: patients with SAPS with an intact rotator cuff and patients with a rotator cuff tear. This guideline focuses on SAPS.

Positive predictive value (PPV) refers to the proportion of individuals with a positive test result who actually have the condition. In Michener’s study, the empty can test (PPV 0.61) and the external rotation resistance test (PPV 0.64) scored highest. However, it is important to note that using multiple tests slightly increases the likelihood of a false positive (PPV 0.54).

Negative predictive value (NPV), the proportion of individuals with a negative test result who truly do not have the condition, ranged from 0.80 to 0.88 in Michener’s study for individual or combined tests.

***Recommendations:***

- Performing a cluster of physical diagnostic tests slightly improves diagnostic accuracy for SAPS compared with a single test. During physical examination, multiple tests should be performed to rule out other pathologies such as rotator cuff tears, labral tears, and biceps tendon pathology, as these may require different treatment approaches.

### Clinical question 3: What is the value of ultrasonography vs MRI in patients suspected of having a partial thickness tear in the supraspinatus tendon?

There is sufficient scientific evidence showing that MRI is not superior to ultrasonography in detecting rotator cuff tears [14]. However, this is less clear for partial-thickness tears. In patients with full-thickness tears, surgical intervention may be considered. If ultrasonography provides a reliable diagnosis of partial-thickness tears in these patients, MRI is not necessary. This helps to avoid costs related to unnecessary imaging.

A systematic literature search resulted in 639 hits. After reading full text, 3 studies were included [14-16]. Based on the evidence synthesis, the quality of evidence was low. The use of ultrasonography may not differ significantly from MRI in terms of false positives, true negatives, specificity, and positive predictive value.

***Recommendation:***

- Perform ultrasonography in patients with clinical suspicion of full-thickness or partial-thickness rupture of the supraspinatus tendon to guide treatment decisions.

### Clinical question 4: What is the effectiveness of barbotage compared with shockwave therapy in patients with calcifying tendinitis on patient-reported outcome measures?

13 studies were initially selected based on title and abstract. After full-text review, 5 studies were included in the analysis [17-21]. The important outcome measures were defined as pain and functioning. The overall evidence quality for pain at 6 months and 12 months was very low due to risk of bias, a limited number of included patients, and inconsistency in reported findings. Thus, the current literature provides limited guidance for decision-making. Conflicting results have been reported for barbotage [22,23]. High-energy shockwave therapy shows significantly better outcomes than low-energy shockwave therapy. However, high-energy shockwave therapy is not routinely available in daily practice and can be very painful.

***Recommendations:***

- Start treatment with corticosteroid injections in the subacromial bursa.
- Consider barbotage for symptomatic calcific tendinosis with corticosteroid injection in the bursa, acknowledging that recent evidence suggests limited additional benefit over corticosteroid injection alone. Barbotage may be most beneficial in selected patients with large calcifications who have failed initial corticosteroid injection.

### Clinical question 5: What are the benefits and harms of subacromial corticosteroid injection in addition to exercise in patients with subacromial shoulder complaints?

The literature search resulted in 264 hits. 5 studies were included [24-28]. The overall evidence quality for pain at 6 months and 12 months was low. No significant difference in pain or function was found between exercise therapy alone and exercise therapy combined with corticosteroid injection. Exercise therapy alone may reduce the risk of recurrence compared with the combination of exercise therapy and corticosteroid injection. Complications were not reported in the included studies. From a patient perspective, corticosteroid injections offer the advantage of quick pain relief, but patients should understand that exercise therapy is essential for long-term resolution.

***Recommendation:***

- Consider a subacromial corticosteroid injection (with a local anesthetic) to enable exercise therapy in patients with severe complaints that impair their ability to participate in exercise therapy.

### Clinical question 6: What is the effectiveness of suprascapular nerve block (SSNB) vs corticosteroid injection in SAPS patients on patient-reported outcome measures?

The literature search resulted in 1 systematic review, which included 11 RCTs [29]. 4 of these RCTs met the PICO criteria but were excluded after full text review. Consequently, no conclusion could be drawn based on the literature. However, an RCT comparing SSNB with corticosteroid injections found after 12 weeks of follow-up significantly better outcomes for the SSNB group in patients with a rotator cuff tear, although this effect was not observed at earlier time points [30]. The workgroup, based on expert opinion, concludes that this effect can also be expected in patients with SAPS and an intact rotator cuff.

***Recommendations:***

- Consider SSNB for patients with therapy-resistant SAPS when other non-surgical treatment is ineffective.

## Discussion

The goal of this guideline is to decrease variation in clinical practice, improve clinical outcomes, and support shared decision-making. As a consequence, patient perspective is therefore incorporated into the guideline. This guideline has been developed to inform all healthcare professionals involved in the management of SAPS, including orthopedic surgeons, physical therapists, anesthesiologists, radiologists, and general practitioners.

The 1st, 5th and 6th clinical questions address non-surgical measures in the treatment of SAPS. For patients with SAPS, strong advice against subacromial decompression surgery was already given in 2021. This module was not updated; strong scientific evidence has emerged showing no primary role for subacromial decompression surgery in patients with SAPS.

### Lifestyle

If a patient suffers from SAPS, preventive measures can be taken to avoid recurrence, which is discussed in clinical question 1. However, no studies were found comparing the effectiveness of preventive measures vs no preventive measures. Factors that negatively influence tissue regeneration, like smoking, obesity, poor physical condition, and long-term use of NSAID or corticosteroids, will also negatively influence SAPS [2,31]. Additionally, poor thoracic posture can negatively influence the outcome of SAPS treatment. The guideline emphasizes shared decision-making and empowering patients with the skills needed to manage their pain effectively [32]. Encouraging patients to maintain a healthy lifestyle is also a key focus. As a result, the guideline committee recommends the restrained use of corticosteroids. These should be considered as a facilitator to support physical therapy (see Question 5), rather than as a standalone treatment.

### Non-surgical therapy

Non-surgical therapy should comprise exercise therapy; however, scientific evidence is lacking on the form or protocol of this therapy. The benefits and harms of subacromial corticosteroid injection in addition to exercise were reviewed in clinical question 5. Little to no difference in pain and function in the exercise therapy group compared with the combined group (exercise and corticosteroid injection) was found. The quality of evidence, however, was low. From the patient’s perspective pain relief is important. In most cases, an injection with corticosteroid and local anesthetic relieves pain directly. The cost of an additional injection alongside exercise therapy is low. In patients with SAPS where pain hinders adequate exercise therapy, an additional injection is advised. When an injection is administered, it is important to inform the patient that the pain relief is temporary and exercise therapy combined with advice to avoid prolonged shoulder strain is warranted to achieve structural healing. In the 6th clinical question, the effectiveness of SSNB vs corticosteroid injection in SAPS patients was evaluated. No studies meeting the inclusion criteria were found comparing the effect of both modalities in SAPS patients with an intact rotator cuff. In an RCT comparing SSNB with corticosteroid injection in patients with a symptomatic cuff rupture, a difference in CMS was found at 12 weeks of follow-up in favor of the SSNB group [30]. However, costs of corticosteroid injection are lower than SSNB, as ultrasonography and the expertise of an anesthesiologist is needed. Therefore, the guideline development group advises the use of SSNB only in patients with therapy-resistant SAPS, where all other non-surgical measures are ineffective.

### Physical examination

The second clinical question focused on the physical examination of patients with SAPS. A systematic review of the literature was conducted to assess the diagnostic accuracy of using a combination of tests vs a single test in diagnosing or ruling out SAPS. 2 studies comparing single tests with combinations of tests for SAPS diagnosis were included [12,13]. Both studies reported sensitivity and specificity as key outcomes, but the level of evidence for these measures was low, resulting in limited confidence in the reported data. The studies were over 10 years old, not all available rotator cuff tests were included, and arthroscopy was used as the gold standard.

The guideline development group considers that there is low-quality evidence proving single tests may be sufficient to diagnose SAPS. Multiple guidelines advise using a cluster of tests to diagnose SAPS. The guideline working group emphasizes the importance of conducting a thorough anamnesis and complete physical examination to rule out other causes of shoulder pain, such as intra-articular lesions (e.g., Bankart lesions) and multidirectional instability mimicking SAPS complaints, AC joint osteoarthritis, massive rotator cuff tears, neurological conditions causing scapular dyskinesia and secondary SAPS, or oncological causes of shoulder pain.

### Ultrasonography vs MRI

In the third clinical question, the value of ultrasonography vs MRI for patients suspected of having a partial thickness tear in the supraspinatus tendon is evaluated. A systematic review including 3 studies was retrieved, which concluded that with reasonable certainty ultrasonography, when performed by an experienced professional using high-end equipment, is equivalent to non-contrast MRI in detecting and ruling out supraspinatus tendon injuries [14-16]. Low quality of evidence suggests no significant difference in PPV and NPV between ultrasonography and MRI for partial-thickness rotator cuff tears.

MRI is superior to ultrasonography for assessing rotator cuff atrophy and fatty infiltration [33]. A fair correlation exists between the Goutallier stage of the supraspinatus and infraspinatus muscles assessed on MRI and CT, compared with the visual assessment of fatty infiltration on ultrasonography.

While the diagnostic accuracy of ultrasonography for full-thickness rotator cuff tears is well established and equivalent to MRI, less data exists for partial-thickness tears. However, as ultrasonography can reliably diagnose or exclude partial-thickness tears, this avoids the need for MRI in many patients, thereby reducing unnecessary imaging costs while maintaining diagnostic accuracy. Our recommendation therefore includes both partial- and full-thickness tears, with MRI reserved for cases where ultrasonography findings are inconclusive or when detailed assessment of muscle atrophy and fatty infiltration is needed for surgical planning.

### Barbotage

For the 4th clinical question, a systematic review addressing the effectiveness of barbotage compared with shockwave in patients with calcifying tendinitis was conducted. 5 studies were included [16-20], with very low evidence quality for pain outcomes at 6 and 12 months due to risk of bias, limited patient numbers, and inconsistent findings.

Recent evidence has added important nuance to this discussion. In October 2023, a rigorous randomized controlled trial by Moosmayer et al. compared barbotage with sham treatment [23]. This study found no significant functional differences between barbotage and sham treatment at 4 and 24 months of follow-up, although patients receiving barbotage showed improved functional scores at 6 weeks. Notably, a key limitation was that complete calcification removal could not be achieved in many patients, which may have influenced outcomes. These findings contrast with an earlier RCT by de Witte et al., which demonstrated statistically significant improvements with barbotage compared with subacromial corticosteroid injection at 1-year follow-up [22].

Several factors may explain these conflicting results. First, the timing of outcome assessment appears critical: recovery following barbotage typically takes 6 to 12 months, which may explain the absence of differences at the 4-month follow-up in the sham-controlled study. Second, calcific tendinitis is a self-limiting condition, which could account for the similar long-term outcomes between treatment groups at 24 months. This natural history was confirmed at 5-year follow-up by de Witte et al. [34]. Third, patient selection may be crucial: post-hoc analyses suggest that patients with larger calcifications may derive greater benefit from barbotage, a hypothesis also proposed by Moosmayer et al. Fourth, the technical success of the procedure—specifically, the ability to completely remove or fragment the calcification—appears to influence outcomes significantly.

Regarding shockwave therapy, there is some evidence for high-energy protocols, though these treatments can be very painful and are not routinely available in daily practice. Convincing evidence for low-energy shockwave therapy is lacking [34].

While evidence suggests that barbotage may provide no clinically meaningful benefit for shoulder pain, it may still be considered in selected cases. Our approach is pragmatic: initiate treatment with a subacromial corticosteroid injection. For patients with persistent symptoms, particularly those with large calcifications visible on imaging, barbotage with additional corticosteroid injection in the bursa represents a reasonable next step, despite limited evidence for additional benefit over injection alone. However, patients should be counseled that (i) current evidence suggests modest additional benefit over corticosteroid injection alone, (ii) the procedure is most likely to benefit those with larger calcifications, (iii) recovery typically takes 6–12 months, and (iv) the condition is often self-limiting regardless of intervention.

This guideline was composed for managing SAPS. In part 1 we have focused on preventive measures, diagnostics, and non-surgical therapy of SAPS.

### Supplementary data

Work group details, conflict of interests, search strategies and evidence tables are available as supplementary data on the article page, doi: 10.2340/17453674.2026.45365

## Supplementary Material



## Notes

All authors contributed to data analysis, participated in consensus meetings, and were involved in writing the manuscript.

Handling co-editor: Jeppe Vejlgaard Rasmussen

*Acta* thanks Teemu Karjalainen and Adam Witten for help with peer review of this manuscript.
