# Supplementary material for: Update of guideline for diagnosis and treatment of subacromial pain syndrome: a multidisciplinary review by the Dutch Orthopedic Association: Part 1: preventive measures, diagnostics, and non-surgical treatment of subacromial pain syndrome
Source: Acta Orthop. 2026 Feb 16;97:91–8. doi: 10.2340/17453674.2026.45365 (PMC12908218; doi:10.2340/17453674.2026.45365)
Supplement: Supplementary file 1 [file ActaO-97-45365-s1.pdf]

Supplementary file

Formaterat: Bredd: 29,66 cm, Höjd: 20,99 cm

Composition of the guideline working group.

Working Group

Dr. J.J.A.M. (Jos) van Raaij, orthopedic surgeon, Martini Hospital Groningen, NOV (Chairman)
Dr. C.P.J. (Cornelis) Visser, orthopedic surgeon, Alrijne and Eisenhower Clinic, NOV
Dr. F.O. (Okke) Lambers Heerspink, orthopedic surgeon, VieCuri Medical Center, NOV
Dr. E.J.D. (Bart Jan) Veen, orthopedic surgeon, Medical Spectrum Twente, NOV
Dr. O. (Oscar) Dorrestijn, orthopedic surgeon, Sint Maartens Clinic, NOV
Dr. M.J.C. (Maarten) Leijs, orthopedic surgeon, Reinier Haga Orthopedic Center, NOV
Dr. D. (Dennis) van Poppel, manual therapist, sports physiotherapist, PECE Zorg, Fontys Paramedical, KNGF
Drs. P.A. (Peter) Stroomberg, radiologist, Isala, NVvR
Dr. R.P.G. (Ramon) Ottenheijm, general practitioner, Department of General Practice, Maastricht University, NHG
Dr. J.W. (Jan Willem) Kallewaard, anesthesiologist, Rijnstate, NVA
Drs. T.J.W. (Tjerk) de Ruiter, rehabilitation physician, De Ruiter Rehabilitation, VRA
Dr. H.A. (Henk) Martens, rheumatologist, Sint Maartens Clinic, NVR

Advisory group

Drs. R.J. (René) Naber, occupational physician, Amsterdam UMC Occupational Health Service, NVAB
Drs. Y.B. (Yvonne) Suijkerbuijk, physician-researcher, Amsterdam UMC and insurance physician, UWV, NVVG

Methodological support

Drs. F.M. (Femke) Janssen, junior advisor, Knowledge Institute of the Federation of Medical Specialists (until October 2023, from May 2024)
Drs. T. (Tessa) Geltink, advisor, Knowledge Institute of the Federation of Medical Specialists (until May 2024)
Dr. M.S. (Matthijs) Ruiter, senior advisor, Knowledge Institute of the Federation of Medical Specialists (from May 2024)
Dr. J.G.M. (Jacqueline) Jennen, advisor, Knowledge Institute of the Federation of Medical Specialists (October 2023 to May 2024)

Declaration of interests

The code "prevention of influence through conflicts of interest" has been followed. All guideline working group members have provided written declarations regarding whether they have had direct financial interests (employment with a commercial company, personal financial interests, research funding) or indirect interests (personal relationships, reputation management) in the last three years.

During the development or revision of a module, any changes in interests were reported. The declaration of interests were reconfirmed during the commentary phase.

An overview of the interests of the working group members and the assessment of how any potential conflicts of interest are managed can be found in Table 1.

The Guideline was financed by the Quality Fund for Medical Specialists (*Stichting Kwaliteitsgelden Medisch Specialisten, SKMS)*, which is a quality fund for medical specialists in the Netherlands. The financier has had no influence on the content of the guideline modules.

Working group member	Function	Secondary positions	Declared interests	Measures undertaken
Van Raaij (chairman)	Chairman Guideline group	Orthopedic surgeon, scientific associate (Orthoresearch North Foundation), Martini Hospital Groningen (unpaid).<br><br>Board member, Shoulder/Elbow Working Group of the Dutch Orthopaedic Association (NOV) (unpaid).<br><br>Member of the Registration Advisory Board (RAR), Dutch National Orthopedic Implant	None	No restrictions

Formaterat: Avstånd Efter: 0 pt, Radavstånd: enkelt

Formaterad tabell

Formaterat: Avstånd Efter: 0 pt, Radavstånd: enkelt

		Registry (LROI) (unpaid).<br><br>Member of LEARN (University of Groningen) – research into training/education (unpaid).<br><br>Course leader for skills training for orthopedic surgery residents (TechMed Centre, University of Twente) (unpaid).<br><br>Member of the working group on the guideline for chronic shoulder instability (FMS, Knowledge Institute).<br><br>Chairperson of the guideline cluster for the upper extremity (FMS, Knowledge Institute).<br><br>Member of the working group developing the guideline for shoulder complaints, KNGF (Royal Dutch Society for Physical Therapy).		
Visser	Orthopedic surgeon Alrijne	Orthopedic surgeon, Eisenhower Clinic<br>Member of the Scientific Advisory Board (WAR), Dutch National Orthopedic Implant Registry (LROI) (unpaid)<br>Member of the Audit Committee	None	No restrictions

Formaterat: Avstånd Efter: 0 pt, Radavstånd: enkelt

		of the Dutch Orthopaedic Association (NOV) (unpaid)		
Lambers Heerspink	Orthopedic surgeon VieCuri Medical Center	Committee of Inquiry, VieCuri Hospital (unpaid)<br>Member of the Scientific Committee, VieCuri Hospital (unpaid)<br>Chairperson of BELG (Upper Extremity Limburg Society) (unpaid)	Presentation for orthopedic company Arthrex on proximal humerus fracture (paid)<br>Externally funded research (Funder, topic):<br>• Arthrex and Science & Innovation Fund VieCuri – Optimal positioning of the glenoid in reverse shoulder prosthesis<br>• Science & Innovation Fund VieCuri – Post-treatment of shoulder prosthesis via app<br>• Science & Innovation Fund VieCuri – Prevention of crystallopathy in patients with a degenerative rotator cuff tear	No restrictions; the subject of the externally funded research falls outside the scope of the guideline.
Veen	Orthopedic surgeon, Medisch Spectrum Twente	None	None	No restrictions
Dorrestijn	Orthopedic surgeon	Employment at Sint Maartenskliniek – however, no direct financial benefit.	None	No restrictions
Leijs	Team doctor for Excelsior and orthopedic surgeon.	None	None	No restrictions
Van Poppel	Manual therapist, sports physiotherapist, movement scientist, lecturer, and researcher at PECE Care,	Independent lecturer, author, and researcher, paid.<br><br>Lecturer in the Master's Program	None	No restrictions

Formaterat: Avstånd Efter: 0 pt, Radavstånd: enkelt

Formaterat: Avstånd Efter: 0 pt, Radavstånd: enkelt

Formaterat: Avstånd Efter: 0 pt, Radavstånd: enkelt

Formaterat: Avstånd Efter: 0 pt, Radavstånd: enkelt

Formaterat: Avstånd Efter: 0 pt, Radavstånd: enkelt

	Shoulder Expertise Center, and Fontys University of Applied Sciences.	in Sports Physiotherapy at Rotterdam University of Applied Sciences, paid.<br><br>Member of the working group developing the guideline for shoulder complaints, KNGF (Royal Dutch Society for Physical Therapy).<br><br>Auditor for Health Care Auditing, paid.<br><br>Member of the Regional Disciplinary Board for Healthcare, paid.		
Stroomberg<br><br>*Participation from 09-10-2023*	*Until 31-10-2024: Radiology Fellow, Rijnstate Hospital*<br>*From 01-11-2024: Radiologist, Isala Hospital*	None	None	No restrictions
Koen<br><br>*Participation until 09-10-2023*	Radiologist at Meander Medical Center, Screening Radiologist for Breast Cancer Screening Program..	None	None	No restrictions
Ottenheijm	University Lecturer; Department of General Practice, Maastricht University; Specialist General Practitioner in Musculoskeletal Medicine: working as a self-employed professional for MCC Omnes, Pluspunt MC, and ZBC Optimus Orthopedics.	Chairperson of the Optimus Clinics Foundation (ZBC) (unpaid)<br><br>Medical Director of Optimus Orthopedics BV (unpaid)	Works as a self-employed specialist general practitioner in 1.5 line outpatient clinics and in an orthopedic ZBC, providing care for shoulder patients.<br>Co-shareholder of Optimus Orthopedics BV.	No restrictions

Formaterat: Avstånd Efter: 0 pt, Radavstånd: enkelt

Formaterat: Avstånd Efter: 0 pt, Radavstånd: enkelt

Formaterat: Avstånd Efter: 0 pt, Radavstånd: enkelt

		Board member of the NHG Expert Group on the Movement Framework (association of specialist general practitioners in musculoskeletal medicine) until June 2022.	Co-applicant for a ZonMW-funded efficiency study on shoulder complaints in general practice (Principal investigator works at Erasmus MC).	
Kallewaard	Anesthesiologist, Rijnstate Hospital	Involved in other guidelines:<br>BBC NVA (Dutch Pain Society) Section Pain, NVA Head Cluster Pain, participant.	Externally funded research (Funder, topic):<br>Boston Scientific (Neuromodulation and Endometriosis)<br>Saluda (Neuromodulation PSPS2)<br>DTM (Neuromodulation Virgin Back)	No restrictions; the subject of the externally funded research falls outside the scope of the guideline.
De Ruiter	Rehabilitation physician at De Ruiter Rehabilitation	Rotterdam Knowledge Ambassador, Unpaid<br><br>Advisor for the Foundation for Mobility for the Disabled, Unpaid<br><br>Founder of Perpetual Prosthetics, Unpaid<br><br>Member of the Membership Committee, International Society on Prosthetics and Orthotics, Unpaid	None	No restrictions
Martens	Rheumatologist Sint Maartenskliniek	None	None	No restrictions

Formaterat: Avstånd Efter: 0 pt, Radavstånd: enkelt

Formaterat: Avstånd Efter: 0 pt, Radavstånd: enkelt

Formaterat: Avstånd Efter: 0 pt, Radavstånd: enkelt

Advisory group

**Sounding Board Group Member**	**Role**	**Secondary functions**	**Declared interests**	**Measures undertaken**
Naber	occupational physician AUMC	Secretary of NVAB Working Group for Occupational Physicians in Healthcare (unpaid)<br><br>Member of NVAB Committee for Guideline Development and Science (unpaid)<br><br>Member of NVAB Committee for Intercollegial Review and Professional Development (unpaid)<br>Member of the Early Detection Alcohol Problems Collaboration (on behalf of NVAB, unpaid)<br><br>Member workinggroup SRI PBM<br>Member workinggroup SRI BRMO<br>Member workinggroup SRI MRSA	None	No restrictions
Suijkerbuijk	Medical researcher (PhD candidate) at the Knowledge Center for Insurance Medicine, Amsterdam UMC, AMC location (paid)<br>Insurance physician at UWV: WIA assessments (paid)	Member of the Science Committee of NVVG: Reviewing and participating in the development of guidelines. Currently involved in the development of the multidisciplinary guideline on Depression (Trimbos) (unpaid)<br><br>Member of the Committee for Occupational and Insurance Physicians at Radboud Health Academy: Organizing symposia; currently involved in a symposium on long-COVID and work (unpaid)	PhD research funded by UWV.	No restrictions

tog bort: Functions

Formaterat: Avstånd Efter: 0 pt, Radavstånd: enkelt

Formaterad tabell

Formaterat: Avstånd Efter: 0 pt, Radavstånd: enkelt

Formaterat: Avstånd Efter: 0 pt, Radavstånd: enkelt

		Member of the Advisory Committee for a UWV-funded implementation study (WerkWeb Autism UMCG): Low-frequency meetings focused on advice/input, no executive role (unpaid)		

Methodological support

Working group member	Function	Secondary positions	Declared interests	Measures undertaken
Geltink	Advisor to the Knowledge Institute of the Federation of Medical Specialists.	None	None	No restrictions
De Ruiter	Senior advisor to the Knowledge Institute of the Federation of Medical Specialists.	None	None	No restrictions
Janssen	Junior advisor to the Knowledge Institute of the Federation of Medical Specialists.	None	None	No restrictions
Jennen	Advisor to the Knowledge Institute of the Federation of Medical Specialists.	None	None	No restrictions

Formaterat: Avstånd Efter: 0 pt, Radavstånd: enkelt

Formaterad tabell

Formaterat: Avstånd Efter: 0 pt, Radavstånd: enkelt

Formaterat: Avstånd Efter: 0 pt, Radavstånd: enkelt

Formaterat: Avstånd Efter: 0 pt, Radavstånd: enkelt

Formaterat: Avstånd Efter: 0 pt, Radavstånd: enkelt

Methodology guideline development process

AGREE

This guideline module was developed in accordance with the requirements outlined in the report Medical Specialist Guidelines 3.0 by the Guideline Advisory Committee of the Quality Council. This report is based on the AGREE II instrument (Appraisal of Guidelines for Research & Evaluation II; Brouwers, 2010).

Problem Analysis and Key Questions

During the preparatory phase, the working group identified challenges in the care for patients with SAPS. Additional challenges were raised by affiliated scientific medical societies: *Health and Youth Care Inspectorate; Dutch Federation of University Medical Centers; Dutch College of General Practitioners; Dutch Hospitals Association*; *Physiotherapy Netherlands*; *Specialist Medical Centers Association*; *Nurses and Care Professionals Netherlands*; *Dutch Association of Anesthesiology and Pain Medicine; Dutch Institute for the Implementation of Healthcare*; *Dutch Association of Healthcare Providers in the Independent Health Sector*; *Health Insurance Netherlands*; *Association of Independent Healthcare Providers*; *Dutch Orthopaedic Association*; *Royal Dutch Society for Physical Therapy*; *Dutch Association for Rehabilitation Medicine*; *Dutch Pain Society*; *Physiotherapy Federation Netherlands*; *Dutch Association of Radiologists*; *Dutch Rheumatism Association*; *Dutch Association for Occupational Medicine*, and insurance physicians through a problem analysis (invitational conference). A report of this is included under related products.

Based on the findings from the problem analysis, the working group drafted and finalized concept key questions.

Outcome measures

After formulating the research question corresponding to the key question, the working group identified patient-relevant outcome measures, considering both desired and undesired effects. A maximum of eight outcome measures was used. The working group ranked these outcome measures according to their relative importance in making recommendations: as critical (essential for decision-making), important (but not critical), or unimportant. For at least the critical outcome measures, the group also defined clinically (patient) relevant differences.

Method of literature summary

A detailed description of the strategy for searching and selecting literature can be found under 'Search and Selection' in the supporting evidence. Where possible, data from different studies were pooled using a [random-effects model]. Statistical analyses were performed using [Review Manager 5.4]. The assessment of the strength of the scientific evidence is explained below.

Assessment of the strength of scientific evidence

The strength of the scientific evidence was determined using the GRADE methodology. GRADE stands for Grading Recommendations Assessment, Development, and Evaluation (see http://www.gradeworkinggroup.org/). The core principles of the GRADE methodology include: identifying and prioritizing clinically (patient) relevant outcome measures, conducting a systematic review for each outcome measure, and evaluating the quality of evidence per outcome measure based on eight GRADE domains (downgrading domains: risk of bias, inconsistency, indirectness, imprecision, and publication bias; upgrading domains: dose-response relationship, large effect, and residual plausible confounding).

GRADE distinguishes four levels of quality for scientific evidence: high, moderate, low, and very low. These levels reflect the degree of confidence in the literature conclusion, particularly the extent to which the conclusion adequately supports the recommendation (Schünemann, 2013; Hultcrantz, 2017).

GRADE	Definition
High certainty	There is high confidence that the true value of the estimate of interest is on one side of a threshold of interest or within a specific range.
Moderate certainty	There is moderately confidence that that the true value of the estimate of interest is on one side of a threshold of interest or within a certain range. The true value of the estimate may deviate slightly from the target of the certainty rating (i.e. may possibly fall in a different range).
Low certainty	There is low confidence that that the true value of the estimate of interest is on one side of a threshold of interest or within a certain range. The true value of the estimate may deviate from the target of the certainty rating (i.e. likely fall in a different range).
Very low certainty	There is very-low confidence that that the true value of the estimate of interest is on one side of a threshold of interest or within a certain range. The true value of the estimate may deviate significantly from target of the certainty rating (i.e. probably fall in a different range).

Table 2. GRADE Levels for the certainty of evidence

Ändrad fältkod

formaterade: Engelska (USA)

tog bort: There is high confidence the true effect of treatment is close to the estimated effect of treatment; it is very unlikely that the literature conclusion will change clinically relevant when results of new large-scale research are added to the literature analysis.

formaterade: Engelska (USA)

tog bort: There is reasonable assurance the true effect of treatment is close to the estimated effect of treatment; it is possible that the conclusion changes clinically relevant when results of new large-scale studies are added to the literature analysis.

formaterade: Engelska (USA)

tog bort: There is low certainty the true effect of treatment is close to the estimated effect of treatment; there is a real chance that the conclusion will change clinically relevant when results of new large-scale research are added to the literature analysis

tog bort: .

formaterade: Engelska (USA)

tog bort: There is very low certainty the true effect of treatment is close to the estimated effect of treatment; the literature conclusion is very uncertain.

tog bort: Classifications

Assessing (grading) the strength of scientific evidence in guidelines using the GRADE methodology

When assessing the strength of scientific evidence in guidelines according to the GRADE methodology, thresholds for clinical decision-making play an important role (Hultcrantz, 2017). These thresholds indicate when a recommendation might need to be adjusted if exceeded. Determining these thresholds requires considering all relevant outcome measures and considerations.

The thresholds for clinical decision-making are therefore not directly equivalent to the Minimal Clinically Important Difference (MCID). Particularly in cases where an intervention has no significant disadvantages and costs are relatively low, the threshold for clinical decision-making regarding the effectiveness of the intervention may be closer to the null effect than the MCID (Hultcrantz, 2017).

Considerations (from evidence to recommendation)

To formulate a recommendation, factors beyond (the quality of) scientific evidence are also important and are taken into account. These include additional arguments from fields like biomechanics or physiology, patient values and preferences, costs (resource use), acceptability, feasibility, and implementation. These aspects are systematically documented and evaluated (weighed) under the heading ‘Considerations’ and may also be based on expert opinion.

A structured format, based on the evidence-to-decision framework of the international GRADE Working Group (Alonso-Coello, 2016a; Alonso-Coello, 2016b), was used for this purpose. This evidence-to-decision framework is an integral component of the GRADE methodology.

Formulating recommendations

Recommendations answer the key question and are based on the available scientific evidence, key considerations, and a weighing of the favorable and unfavorable effects of the relevant interventions. The strength of the scientific evidence, combined with the weight assigned by the working group to the considerations, determines the strength of the recommendation.

In line with the GRADE methodology, low-quality evidence from conclusions in the systematic literature analysis does not automatically exclude strong recommendations, and weak recommendations are possible even with high-quality evidence (Agoritsas, 2017;

Neumann, 2016). The strength of a recommendation is always determined by weighing all relevant arguments together. The working group documented the rationale behind the direction and strength of each recommendation.

In the GRADE methodology, a distinction is made between strong and weak (or conditional) recommendations. The strength of a recommendation reflects the level of confidence that the benefits of the intervention outweigh the harms (or vice versa) across the entire spectrum of patients for whom the recommendation is intended.

The strength of a recommendation has clear implications for patients, healthcare providers, and policymakers (see table below). A recommendation is not a mandate; even a strong recommendation based on high-quality evidence (GRADE rating HIGH) will not always be applicable in every possible circumstance or for every individual patient.

Organization of care

In the problem analysis and during the development of the guideline module, explicit attention was given to the organization of care: all aspects that are essential prerequisites for providing care (such as coordination, communication, (financial) resources, manpower, and infrastructure). Prerequisites relevant to addressing this specific key question are mentioned under the considerations. More general, overarching, or additional aspects of the organization of care are addressed in the module Organization of Care.

Commentary and authorization phase

The draft guideline module was presented to the involved (scientific) associations and (patient) organizations for feedback. Comments were collected and discussed with the working group. Based on the comments, the draft guideline module was revised and finalized by the working group. The final guideline module was submitted to the participating (scientific) associations and (patient) organizations for authorization and was subsequently authorized or approved by them.

Literature

Agoritsas T, Merglen A, Heen AF, Kristiansen A, Neumann I, Brito JP, Brignardello-Petersen R, Alexander PE, Rind DM, Vandvik PO, Guyatt GH. UpToDate adherence to GRADE criteria for strong recommendations: an analytical survey. BMJ Open. 2017 Nov 16;7(11):e018593. doi: 10.1136/bmjopen-2017-018593. PubMed PMID: 29150475; PubMed Central PMCID: PMC5701989.

Alonso-Coello P, Schünemann HJ, Moberg J, Brignardello-Petersen R, Akl EA, Davoli M, Treweek S, Mustafa RA, Rada G, Rosenbaum S, Morelli A, Guyatt GH, Oxman AD; GRADE Working Group. GRADE Evidence to Decision (EtD) frameworks: a systematic and transparent approach to making well informed healthcare choices. 1: Introduction. BMJ. 2016 Jun 28;353:i2016. doi: 10.1136/bmj.i2016. PubMed PMID: 27353417.

Alonso-Coello P, Oxman AD, Moberg J, Brignardello-Petersen R, Akl EA, Davoli M, Treweek S, Mustafa RA, Vandvik PO, Meerpohl J, Guyatt GH, Schünemann HJ; GRADE Working Group. GRADE Evidence to Decision (EtD) frameworks: a systematic and transparent approach to making well informed healthcare choices. 2: Clinical practice guidelines. BMJ. 2016 Jun 30;353:i2089. doi: 10.1136/bmj.i2089. PubMed PMID: 27365494.

Brouwers MC, Kho ME, Browman GP, Burgers JS, Cluzeau F, Feder G, Fervers B, Graham ID, Grimshaw J, Hanna SE, Littlejohns P, Makarski J, Zitzelsberger L; AGREE Next Steps Consortium. AGREE II: advancing guideline development, reporting and evaluation in health care. CMAJ. 2010 Dec 14;182(18):E839-42. doi: 10.1503/cmaj.090449. Epub 2010 Jul 5. Review. PubMed PMID: 20603348; PubMed Central PMCID: PMC3001530.

Hultcrantz M, Rind D, Akl EA, Treweek S, Mustafa RA, Iorio A, Alper BS, Meerpohl JJ, Murad MH, Ansari MT, Katikireddi SV, Östlund P, Tranæus S, Christensen R, Gartlehner G, Brozek J, Izcovich A, Schünemann H, Guyatt G. The GRADE Working Group clarifies the construct of certainty of evidence. J Clin Epidemiol. 2017 Jul;87:4-13. doi: 10.1016/j.jclinepi.2017.05.006. Epub 2017 May 18. PubMed PMID: 28529184; PubMed Central PMCID: PMC6542664.

Medisch Specialistische Richtlijnen 2.0 (2012). Adviescommissie Richtlijnen van de Raad Kwalitieit. http://richtlijnendatabase.nl/over_deze_site/over_richtlijnontwikkeling.html

Neumann I, Santesso N, Akl EA, Rind DM, Vandvik PO, Alonso-Coello P, Agoritsas T, Mustafa RA, Alexander PE, Schünemann H, Guyatt GH. A guide for health professionals to interpret and use recommendations in guidelines developed with the GRADE approach. J Clin Epidemiol. 2016 Apr;72:45-55. doi: 10.1016/j.jclinepi.2015.11.017. Epub 2016 Jan 6. Review. PubMed PMID: 26772609.

Schünemann H, Brożek J, Guyatt G, et al. GRADE handbook for grading quality of evidence and strength of recommendations. Updated October 2013. The GRADE Working Group, 2013. Available from http://gdt.guidelinedevelopment.org/central_prod/_design/client/handbook/handbook.html.

Module 1

Search and select
A systematic review of the literature was performed to answer the following question: What preventive measures can be used in the working population to prevent recurrent SAPS?

Patients	patients who had SAPS complaints and have recovered
Intervention	preventive measures
Control	no preventive measures
Outcomes	SAPS complaints (pain), return to work or leisure, health care consumption

Search and select (Methods)

The databases Medline (via OVID) and Embase (via Embase.com) were searched with relevant search terms from 2000 until 19 June 2023. The detailed search strategy is depicted under the tab Methods. The systematic orienting literature search resulted in 683 hits. Studies were selected based on the following criteria:

- Study design: randomized controlled trial, systematic review or observational studies about recurrence of SAPS and preventive measures.
- Describing at least one of the relevant outcomes specified in the PICO.

A systematic search resulted in 683 articles that were selected for title and abstract screening. After the title and abstract screening, none of these articles met the selection criteria. Full-text selection was therefore not performed.

Results

No studies were included in the analysis of the literature.

Embase.com

No.	Query	Results
#13	#10 OR #11 OR #12	411
#12	#5 AND (#8 OR #9) NOT (#10 OR #11) = observationeel	251
#11	#5 AND #7 NOT #10 = RCT	112
#10	#5 AND #6 = SR	48
#9	'case control study'/de OR 'comparative study'/exp OR 'control group'/de OR 'controlled study'/de OR 'controlled clinical trial'/de OR 'crossover procedure'/de OR 'double blind procedure'/de OR 'phase 2 clinical trial'/de OR 'phase 3 clinical trial'/de OR 'phase 4 clinical trial'/de OR 'pretest posttest design'/de OR 'pretest posttest control group design'/de OR 'quasi experimental study'/de OR 'single blind procedure'/de OR 'triple blind procedure'/de OR (((control OR controlled) NEAR/6 trial):ti,ab,kw) OR (((control OR controlled) NEAR/6 (study OR studies)):ti,ab,kw) OR (((control OR controlled) NEAR/1 active):ti,ab,kw) OR 'open label*':ti,ab,kw OR (((double OR two OR three OR multi OR trial) NEAR/1 (arm OR arms)):ti,ab,kw) OR ((allocat* NEAR/10 (arm OR arms)):ti,ab,kw) OR placebo*:ti,ab,kw OR 'sham-control*':ti,ab,kw OR (((single OR double OR triple OR assessor) NEAR/1 (blind* OR masked)):ti,ab,kw) OR nonrandom*:ti,ab,kw OR 'non-random*':ti,ab,kw OR 'quasi-experiment*':ti,ab,kw OR crossover:ti,ab,kw OR 'cross over':ti,ab,kw OR 'parallel group*':ti,ab,kw OR 'factorial trial':ti,ab,kw OR ((phase NEAR/5 (study OR trial)):ti,ab,kw) OR ((case* NEAR/6 (matched OR control*)):ti,ab,kw) OR ((match* NEAR/6 (pair OR pairs OR cohort* OR control* OR group* OR healthy OR age OR sex OR gender OR patient* OR subject* OR participant*)):ti,ab,kw) OR ((propensity NEAR/6 (scor* OR match*)):ti,ab,kw) OR versus:ti OR vs:ti OR compar*:ti OR ((compar* NEAR/1 study):ti,ab,kw) OR (('major clinical study'/de OR 'clinical study'/de OR 'cohort analysis'/de OR 'observational study'/de OR 'cross-sectional study'/de OR 'multicenter study'/de OR 'correlational study'/de OR 'follow up'/de OR	14177310

Formaterat: Avstånd Efter: 0 pt, Radavstånd: enkelt

Formaterat: Avstånd Efter: 0 pt, Radavstånd: enkelt

Formaterat: Avstånd Efter: 0 pt, Radavstånd: enkelt

Formaterat: Avstånd Efter: 0 pt, Radavstånd: enkelt

Formaterat: Avstånd Efter: 0 pt, Radavstånd: enkelt

Formaterat: Avstånd Efter: 0 pt, Radavstånd: enkelt

	cohort*:ti,ab,kw OR 'follow up':ti,ab,kw OR followup:ti,ab,kw OR longitudinal*:ti,ab,kw OR prospective*:ti,ab,kw OR retrospective*:ti,ab,kw OR observational*:ti,ab,kw OR 'cross sectional*':ti,ab,kw OR cross?ectional*:ti,ab,kw OR multicent*:ti,ab,kw OR 'multi-cent*':ti,ab,kw OR consecutive*:ti,ab,kw) AND (group:ti,ab,kw OR groups:ti,ab,kw OR subgroup*:ti,ab,kw OR versus:ti,ab,kw OR vs:ti,ab,kw OR compar*:ti,ab,kw OR 'odds ratio*':ab OR 'relative odds':ab OR 'risk ratio*':ab OR 'relative risk*':ab OR 'rate ratio':ab OR aor:ab OR arr:ab OR rrr:ab OR ((('or' OR 'rr') NEAR/6 ci):ab)))	
#8	'major clinical study'/de OR 'clinical study'/de OR 'case control study'/de OR 'family study'/de OR 'longitudinal study'/de OR 'retrospective study'/de OR 'prospective study'/de OR 'comparative study'/de OR 'cohort analysis'/de OR ((cohort NEAR/1 (study OR studies)):ab,ti) OR (('case control' NEAR/1 (study OR studies)):ab,ti) OR (('follow up' NEAR/1 (study OR studies)):ab,ti) OR (observational NEAR/1 (study OR studies)) OR ((epidemiologic NEAR/1 (study OR studies)):ab,ti) OR (('cross sectional' NEAR/1 (study OR studies)):ab,ti)	6767914
#7	'clinical trial'/exp OR 'randomization'/exp OR 'single blind procedure'/exp OR 'double blind procedure'/exp OR 'crossover procedure'/exp OR 'placebo'/exp OR 'prospective study'/exp OR rct:ab,ti OR random*:ab,ti OR 'single blind':ab,ti OR 'randomised controlled trial':ab,ti OR 'randomized controlled trial'/exp OR placebo*:ab,ti	3302394
#6	'meta analysis'/exp OR 'meta analysis (topic)'/exp OR metaanaly*:ti,ab OR 'meta analy*':ti,ab OR metanaly*:ti,ab OR 'systematic review'/de OR 'cochrane database of systematic reviews'/jt OR prisma:ti,ab OR prospero:ti,ab OR (((systemati* OR scoping OR umbrella OR 'structured literature') NEAR/3 (review* OR overview*)):ti,ab) OR ((systemic* NEAR/1 review*):ti,ab) OR (((systemati* OR literature OR database* OR 'data base*') NEAR/10 search*):ti,ab) OR (((structured OR comprehensive* OR systemic*) NEAR/3 search*):ti,ab) OR (((literature NEAR/3 review*):ti,ab) AND (search*:ti,ab OR database*:ti,ab OR 'data base*':ti,ab)) OR (('data extraction':ti,ab OR 'data source*':ti,ab) AND 'study selection':ti,ab) OR ('search strategy':ti,ab AND 'selection criteria':ti,ab) OR ('data source*':ti,ab AND 'data synthesis':ti,ab) OR medline:ab OR pubmed:ab OR embase:ab OR cochrane:ab OR (((critical OR rapid) NEAR/2 (review* OR overview* OR synthes*)):ti) OR ((((critical* OR rapid*)	

Formaterat: Avstånd Efter: 0 pt, Radavstånd: enkelt

Formaterat: Avstånd Efter: 0 pt, Radavstånd: enkelt

Formaterat: Avstånd Efter: 0 pt, Radavstånd: enkelt

	NEAR/3 (review* OR overview* OR synthes*)):ab) AND (search*:ab OR database*:ab OR 'data base*':ab)) OR metasynthes*:ti,ab OR 'meta synthes*':ti,ab	
#5	#4 AND [2000-2023]/py	608
#4	#3 NOT ('conference abstract'/it OR 'editorial'/it OR 'letter'/it OR 'note'/it) NOT (('animal'/exp OR 'animal experiment'/exp OR 'animal model'/exp OR 'nonhuman'/exp) NOT 'human'/exp)	718
#3	#1 AND #2	861
#2	'recurrence risk'/exp OR 'recurrent disease'/exp OR recurr*:ab,ti,kw OR relaps*:ab,ti,kw OR recrudesc*:ab,ti,kw	1450947
#1	'shoulder impingement syndrome'/exp/mj OR 'shoulder impingement syndrome':ti,ab,kw OR 'subacromial impingement syndrome':ti,ab,kw OR (('bursitis'/exp/mj OR bursitis:ti,ab,kw OR 'tendinitis'/exp/mj OR tendinitis:ti,ab,kw OR 'impingement':ti,ab,kw) AND ('shoulder'/exp/mj OR 'rotator cuff':ti,ab,kw OR acromion:ti,ab,kw OR acromial:ti,ab,kw OR subacromial:ti,ab,kw OR shoulder*:ti,ab,kw)) OR 'rotator cuff injury'/exp/mj OR 'rotator cuff rupture'/exp/mj OR ((('rotator cuff' OR 'shoulder cuff' OR infraspinatus OR supraspinatus OR subscapularis) NEAR/3 (tear* OR lesion* OR rupture* OR tendinopath* OR laceration*)):ti,ab,kw) OR (('full-thickness':ti,ab,kw OR partial:ti,ab,kw) AND 'rotator cuff':ti,ab,kw) OR ('rotator cuff'/exp/mj AND ('rupture'/exp/mj OR 'injury'/mj OR 'laceration'/exp/mj))	18074

Ovid/Medline

#	Searches	Results
13	10 or 11 or 12	570
12	(5 and (8 or 9)) not (10 or 11) = observationeel	420
11	(5 and 7) not 10 = RCT	95
10	5 and 6 = SR	55
9	Case-control Studies/ or clinical trial, phase ii/ or clinical trial, phase iii/ or clinical trial, phase iv/ or comparative study/ or control groups/ or controlled	5448124

Formaterat: Avstånd Efter: 0 pt, Radavstånd: enkelt
Formaterat: Avstånd Efter: 0 pt, Radavstånd: enkelt
Formaterat: Avstånd Efter: 0 pt, Radavstånd: enkelt
Formaterat: Avstånd Efter: 0 pt, Radavstånd: enkelt
Formaterat: Avstånd Efter: 0 pt, Radavstånd: enkelt
Formaterat: Avstånd Efter: 0 pt, Radavstånd: enkelt
Formaterat: Avstånd Efter: 0 pt, Radavstånd: enkelt
Formaterat: Avstånd Efter: 0 pt, Radavstånd: enkelt
Formaterat: Avstånd Efter: 0 pt, Radavstånd: enkelt
Formaterat: Avstånd Efter: 0 pt, Radavstånd: enkelt
Formaterat: Avstånd Efter: 0 pt, Radavstånd: enkelt

	before-after studies/ or controlled clinical trial/ or double-blind method/ or historically controlled study/ or matched-pair analysis/ or single-blind method/ or (((control or controlled) adj6 (study or studies or trial)) or (compar* adj (study or studies)) or ((control or controlled) adj1 active) or "open label*" or ((double or two or three or multi or trial) adj (arm or arms)) or (allocat* adj10 (arm or arms)) or placebo* or "sham-control*" or ((single or double or triple or assessor) adj1 (blind* or masked)) or nonrandom* or "non-random*" or "quasi-experiment*" or "parallel group*" or "factorial trial" or "pretest posttest" or (phase adj5 (study or trial)) or (case* adj6 (matched or control*)) or (match* adj6 (pair or pairs or cohort* or control* or group* or healthy or age or sex or gender or patient* or subject* or participant*)) or (propensity adj6 (scor* or match*))).ti,ab,kf. or (confounding adj6 adjust*).ti,ab. or (versus or vs or compar*).ti. or ((exp cohort studies/ or epidemiologic studies/ or multicenter study/ or observational study/ or seroepidemiologic studies/ or (cohort* or 'follow up' or followup or longitudinal* or prospective* or retrospective* or observational* or multicent* or 'multi-cent*' or consecutive*).ti,ab,kf.) and ((group or groups or subgroup* or versus or vs or compar*).ti,ab,kf. or ('odds ratio*' or 'relative odds' or 'risk ratio*' or 'relative risk*' or aor or arr or rrr).ab. or (("OR" or "RR") adj6 CI).ab.))	
8	Epidemiologic studies/ or case control studies/ or exp cohort studies/ or Controlled Before-After Studies/ or Case control.tw. or cohort.tw. or Cohort analy$.tw. or (Follow up adj (study or studies)).tw. or (observational adj (study or studies)).tw. or Longitudinal.tw. or Retrospective*.tw. or prospective*.tw. or consecutive*.tw. or Cross sectional.tw. or Cross-sectional studies/ or historically controlled study/ or interrupted time series analysis/ [Onder exp cohort studies vallen ook longitudinale, prospectieve en retrospectieve studies]	4464295
7	exp clinical trial/ or randomized controlled trial/ or exp clinical trials as topic/ or randomized controlled trials as topic/ or Random Allocation/ or Double-Blind Method/ or Single-Blind Method/ or (clinical trial, phase i or clinical trial, phase ii or clinical trial, phase iii or clinical trial, phase iv or controlled clinical trial or randomized controlled trial or multicenter study or clinical trial).pt. or random*.ti,ab. or (clinic* adj trial*).tw. or ((singl* or doubl* or treb* or tripl*) adj (blind$3 or mask$3)).tw. or Placebos/ or placebo*.tw.	2600532

Formaterat: Avstånd Efter: 0 pt, Radavstånd: enkelt

Formaterat: Avstånd Efter: 0 pt, Radavstånd: enkelt

6	meta-analysis/ or meta-analysis as topic/ or (metaanaly* or meta-analy* or metanaly*).ti,ab,kf. or systematic review/ or cochrane.jw. or (prisma or prospero).ti,ab,kf. or ((systemati* or scoping or umbrella or "structured literature") adj3 (review* or overview*)).ti,ab,kf. or (systemic* adj1 review*).ti,ab,kf. or ((systemati* or literature or database* or data-base*) adj10 search*).ti,ab,kf. or ((structured or comprehensive* or systemic*) adj3 search*).ti,ab,kf. or ((literature adj3 review*) and (search* or database* or data-base*)).ti,ab,kf. or (("data extraction" or "data source*") and "study selection").ti,ab,kf. or ("search strategy" and "selection criteria").ti,ab,kf. or ("data source*" and "data synthesis").ti,ab,kf. or (medline or pubmed or embase or cochrane).ab. or ((critical or rapid) adj2 (review* or overview* or synthes*)).ti. or (((critical* or rapid*) adj3 (review* or overview* or synthes*)) and (search* or database* or data-base*)).ab. or (metasynthes* or meta-synthes*).ti,ab,kf.	674975
5	limit 4 to yr="2000 -Current"	825
4	3 not (comment/ or editorial/ or letter/) not ((exp animals/ or exp models, animal/) not humans/)	965
3	1 and 2	993
2	exp Recurrence/ or (recurr* or relaps* or recrudesc*).ti,ab,kf.	953623
1	Shoulder Impingement Syndrome/ or 'shoulder impingement syndrome'.ti,ab,kf. or 'subacromial impingement syndrome'.ti,ab,kf. or ((exp bursitis/ or exp Tendinopathy/ or bursitis.ti,ab,kf. or tendinitis.ti,ab,kf. or 'impingement'.ti,ab,kf.) and (exp Shoulder/ or 'rotator cuff'.ti,ab,kf. or acromion.ti,ab,kf. or acromial.ti,ab,kf. or subacromial.ti,ab,kf. or shoulder*.ti,ab,kf.)) or Rotator Cuff/ or exp Rotator Cuff Injuries/ or (('rotator cuff' or 'shoulder cuff' or infraspinatus or supraspinatus or subscapularis) adj3 (tear* or lesion* or rupture* or tendinopath* or laceration*)).ti,ab,kf. or (('full-thickness' or partial) and 'rotator cuff').ti,ab,kf.	19733

Formaterat: Avstånd Efter: 0 pt, Radavstånd: enkelt

Formaterat: Avstånd Efter: 0 pt, Radavstånd: enkelt

Formaterat: Avstånd Efter: 0 pt, Radavstånd: enkelt

Formaterat: Avstånd Efter: 0 pt, Radavstånd: enkelt

Formaterat: Avstånd Efter: 0 pt, Radavstånd: enkelt

Formaterat: Avstånd Efter: 0 pt, Radavstånd: enkelt

Module 2

What is the diagnostic accuracy for using a combination of multiple tests compared to a single test in diagnosing or ruling out of SAPS?

Patients	patients with (suspected) SAPS, with exception of a subscapularis rupture
Index test	combination of multiple tests (for example empty can/neer/painful arc/exorotation againts resistance /Yocum/Hawkins)
Comparator test	solitary test (for example Hawkins)
Reference standard	imaging (ultrasound or MRI) or arthroscopy
Outcomes	pain, functionality, return to work or leisure, diagnostic test accuracy measures (sensitivity, specificity)
Timing and setting	outpatient orthopedic consultation

Zoekstrategie

Embase.com

No.	Query	Results
#13	#10 OR #11 OR #12	366
#12	#7 NOT (#10 OR #11) **= overige studies**	293
#11	#7 AND #9 NOT #10 **= RCT**	35
#10	#7 AND #8 **= SR**	38
#9	'randomized controlled trial'/exp OR random*:ti,ab OR (((pragmatic OR practical) NEAR/1 'clinical trial*'):ti,ab) OR ((('non inferiority' OR noninferiority OR superiority OR equivalence) NEAR/3 trial*):ti,ab) OR rct:ti,ab,kw	2018091

#8	'meta analysis'/exp OR 'meta analysis (topic)'/exp OR metaanaly*:ti,ab OR 'meta analy*':ti,ab OR metanaly*:ti,ab OR 'systematic review'/de OR 'cochrane database of systematic reviews'/jt OR prisma:ti,ab OR prospero:ti,ab OR (((systemati* OR scoping OR umbrella OR 'structured literature') NEAR/3 (review* OR overview*)):ti,ab) OR ((systemic* NEAR/1 review*):ti,ab) OR (((systemati* OR literature OR database* OR 'data base*') NEAR/10 search*):ti,ab) OR (((structured OR comprehensive* OR systemic*) NEAR/3 search*):ti,ab) OR (((literature NEAR/3 review*):ti,ab) AND (search*:ti,ab OR database*:ti,ab OR 'data base*':ti,ab)) OR (('data extraction':ti,ab OR 'data source*':ti,ab) AND 'study selection':ti,ab) OR ('search strategy':ti,ab AND 'selection criteria':ti,ab) OR ('data source*':ti,ab AND 'data synthesis':ti,ab) OR medline:ab OR pubmed:ab OR embase:ab OR cochrane:ab OR (((critical OR rapid) NEAR/2 (review* OR overview* OR synthes*)):ti) OR ((((critical* OR rapid*) NEAR/3 (review* OR overview* OR synthes*)):ab) AND (search*:ab OR database*:ab OR 'data base*':ab)) OR metasynthes*:ti,ab OR 'meta synthes*':ti,ab	733409
#7	#4 AND #5 AND #6 AND ([english]/lim OR [dutch]/lim) AND [2008-2023]/py NOT ('conference abstract'/it OR 'editorial'/it OR 'letter'/it OR 'note'/it) NOT (('animal experiment'/exp OR 'animal model'/exp OR 'nonhuman'/exp) NOT 'human'/exp)	366
#6	'diagnostic procedure'/exp OR diagnos*:ti,ab OR 'sensitivity and specificity'/de OR sensitiv*:ab,ti OR specific*:ab,ti OR predict*:ti,kw OR 'roc curve':ab,ti OR 'receiver operator':ab,ti OR 'receiver operators':ab,ti OR likelihood:ab,ti OR 'diagnostic error'/exp OR 'diagnostic accuracy'/exp OR 'diagnostic test accuracy study'/exp OR 'inter observer':ab,ti OR 'intra observer':ab,ti OR interobserver:ab,ti OR intraobserver:ab,ti OR validity:ab,ti OR kappa:ab,ti OR reliability:ab,ti OR reproducibility:ab,ti OR ((test NEAR/2 're-test'):ab,ti) OR ((test NEAR/2 'retest'):ab,ti) OR 'reproducibility'/exp OR accuracy:ab,ti OR 'differential diagnosis'/exp OR 'validation study'/de OR 'measurement precision'/exp OR 'diagnostic value'/exp OR 'reliability'/exp OR 'predictive value'/exp OR ppv:ti,ab,kw OR npv:ti,ab,kw OR 'logistic regression analysis'/exp OR 'area under the curve'/exp OR 'logistic regression*':ti,ab,kw OR auc:ti,ab,kw OR 'area under the curve':ti,ab,kw	24732558
#5	'physical examination'/exp/mj OR 'functional assessment'/exp/mj OR 'physical examination test*':ti,ab,kw OR 'physical test*':ti,ab,kw OR 'index test*':ti,ab,kw OR test*:ti OR (((empty OR full) NEAR/3 can):ti,ab,kw) OR neer:ti,ab,kw OR 'painful arc':ti,ab,kw OR yocum*:ti,ab,kw OR hawkins*:ti,ab,kw OR (((exorotation OR rotation) NEAR/5 (resistance OR resisted)):ti,ab,kw) OR jobe*:ti,ab,kw OR kennedy*:ti,ab,kw	617431

#4	'shoulder impingement syndrome'/exp/mj OR 'shoulder impingement syndrome':ti,ab,kw OR 'subacromial impingement syndrome':ti,ab,kw OR (('bursitis'/exp/mj OR bursitis:ti,ab,kw OR 'tendinitis'/exp/mj OR tendinitis:ti,ab,kw OR 'impingement':ti,ab,kw) AND ('shoulder'/exp/mj OR 'rotator cuff':ti,ab,kw OR acromion:ti,ab,kw OR acromial:ti,ab,kw OR subacromial:ti,ab,kw OR shoulder*:ti,ab,kw)) OR 'rotator cuff injury'/exp/mj OR 'rotator cuff rupture'/exp/mj OR ((('rotator cuff' OR 'shoulder cuff' OR infraspinatus OR supraspinatus OR subscapularis) NEAR/3 (tear* OR lesion* OR rupture* OR tendinopath* OR laceration*)):ti,ab,kw) OR (('full-thickness':ti,ab,kw OR partial:ti,ab,kw) AND 'rotator cuff':ti,ab,kw) OR ('rotator cuff'/exp/mj AND ('rupture'/exp/mj OR 'injury'/mj OR 'laceration'/exp/mj))	17682

Ovid/Medline

#	Searches	Results
11	8 or 9 or 10	447
10	5 not (8 or 9) **= overage studies**	359
9	(5 and 7) not 8 **= RCT**	48
8	5 and 6 **= SR**	40
7	exp randomized controlled trial/ or randomized controlled trials as topic/ or random*.ti,ab. or rct?.ti,ab. or ((pragmatic or practical) adj "clinical trial*").ti,ab,kf. or ((non-inferiority or noninferiority or superiority or equivalence) adj3 trial*).ti,ab,kf.	1588225
6	meta-analysis/ or meta-analysis as topic/ or (metaanaly* or meta-analy* or metanaly*).ti,ab,kf. or systematic review/ or cochrane.jw. or (prisma or prospero).ti,ab,kf. or ((systemati* or scoping or umbrella or "structured literature") adj3 (review* or overview*)).ti,ab,kf. or (systemic* adj1 review*).ti,ab,kf. or ((systemati* or literature or database* or data-base*) adj10 search*).ti,ab,kf. or ((structured or comprehensive* or systemic*) adj3 search*).ti,ab,kf. or ((literature adj3 review*) and (search* or database* or data-base*)).ti,ab,kf. or (("data extraction" or "data source*") and "study selection").ti,ab,kf. or ("search strategy" and "selection criteria").ti,ab,kf. or ("data source*" and "data synthesis").ti,ab,kf. or (medline or pubmed or embase or cochrane).ab. or ((critical or rapid) adj2 (review* or overview* or synthes*)).ti. or (((critical* or rapid*) adj3 (review* or overview* or synthes*)) and (search* or database* or data-base*)).ab. or (metasynthes* or meta-synthes*).ti,ab,kf.	649856
5	limit 4 to ((english language or dutch) and yr="2008 -Current")	447
4	1 and 2 and 3	626

3	exp "Sensitivity and Specificity"/ or (Sensitiv* or Specific*).ti,ab. or predict*.ti,kf. or (ROC-curve or receiver-operator*).ti,ab. or (likelihood or LR*).ti,ab. or exp Diagnostic Errors/ or (inter-observer or intra-observer or interobserver or intraobserver or validity or kappa or reliability).ti,ab. or reproducibility.ti,ab. or (test adj2 (re-test or retest)).ti,ab. or "Reproducibility of Results"/ or accuracy.ti,ab. or Diagnosis, Differential/ or Validation Study/ or exp "Predictive Value of Tests"/ or ppv.ti,ab. or npv.ti,ab. or diagnos*.ti,ab. or predict*.ab,ti. or exp Logistic Models/ or exp Regression Analysis/ or 'logistic regression*'*.ti,ab,kf. or AUC*.ti,ab,kf. or 'area under the curve'*.ti,ab,kf.	9902765
2	exp *Physical Examination/ or 'physical examination test*'.ti,ab,kf. or 'physical test*'.ti,ab,kf. or 'index test*'.ti,ab,kf. or test*.ti. or (empty adj3 can).ti,ab,kf. or neer.ti,ab,kf. or 'painful arc'.ti,ab,kf. or yocum*.ti,ab,kf. or Hawkins*.ti,ab,kf. or ((exorotation or rotation) adj5 (resistance or resisted)).ti,ab,kf. or Jobe*.ti,ab,kf. or Kennedy*.ti,ab,kf.	891795
1	*Shoulder Impingement Syndrome/ or 'shoulder impingement syndrome'.ti,ab,kf. or 'subacromial impingement syndrome'.ti,ab,kf. or ((exp *bursitis/ or exp *Tendinopathy/ or bursitis.ti,ab,kf. or tendinitis.ti,ab,kf. or 'impingement'.ti,ab,kf.) and (exp *Shoulder/ or 'rotator cuff'.ti,ab,kf. or acromion.ti,ab,kf. or acromial.ti,ab,kf. or subacromial.ti,ab,kf. or shoulder*.ti,ab,kf.)) or *Rotator Cuff/ or exp *Rotator Cuff Injuries/ or (('rotator cuff' or 'shoulder cuff' or infraspinatus or supraspinatus or subscapularis) adj3 (tear* or lesion* or rupture* or tendinopath* or laceration*)).ti,ab,kf. or (('full-thickness' or partial) and 'rotator cuff').ti,ab,kf.	17825

Evidence table module 2

Study reference	Study characteristics	Patient characteristics	Index test (test of interest)	Reference test	Follow-up	Outcome measures and effect size	Comments
Michener, 2009	Type of study: Diagnostic test accuracy study (prospective, blinded study design)<br><br>Setting and country: Orthopedic surgeon shoulder clinic<br><br>Funding and conflicts of interest: No commercial party having a direct financial interest in the results	Inclusion criteria:<br>- Consecutive patients presenting with shoulder pain to an orthopedic surgeon's office<br>- patients had to report shoulder pain for at least 1 week, and shoulder pain had to be their primary complaint.<br><br>Exclusion criteria: Not reported.<br><br>Characteristics N=55 patients<br><br>Age,mean ± SD (range): 40.6 ± 15.1 years (range 18–83y)<br><br>Sex: 47 M/8 F<br><br>Average symptom duration 33.8 ± 48.9 months (range 2-230 months)	5 shoulder tests were compared to each other:<br>Neer, Hawkins-Kennedy, painful arc, empty can (Jobe), and external rotation resistance test.<br><br>Neer test: the Neer test was performed with the examiner stabilizing the scapula with a downward force while fully flexing the humerus overhead maximally while applying overpressure. A positive test was reproduction of pain of the superior shoulder.<br><br>Hawkins-Kennedy test: performed by the examiner flexing the humerus and elbow to 90° and then maximally internally rotating the shoulder and applying overpressure. A positive test was reproduction of pain of the superior shoulder.<br><br>Painful arc: performed by asking the patient to actively abduct his/her shoulder and report any pain during abduction. If pain of the superior shoulder was noted between 60° and 120° of abduction, the test was considered positive.<br><br>Empty can test (Jobe test): performed by the examiner elevating the shoulder to 90° in the scapular plane (30°– 40° anterior to the coronal plane) and then placing the shoulder in internal	The operative findings were used as the reference standard, and the patients were classified as positive or negative for SAIS based on the surgical findings.<br><br>Description:<br>The reference standard was determined via operative findings reported by an operative surgeon blinded to the clinical examination findings.<br><br>The intraoperative reference standard criteria for SAIS were the presence of any of the following: visually enlarged bursa, fibrotic appearing bursa, or degeneration of the supraspinatus tendon at the superficial aspect. Patients with additional shoulder pathologies such as partial or full-thickness rotator cuff tears, labral tears, or fraying and instability were not excluded.	After completion of the history and physical examination, the patients underwent an arthroscopic examination within an average of 2.6 months (+-2.7mo, range: 1d–8mo) after the clinical examination.<br><br>There were no missing values.	Outcome measures and effect size<br><br>ROC curve analysis:<br><br>The cut point to discriminate between patients with and without SAIS was 3 positive tests out of 5 (AUC=0.79, 95% CI 0.66–0.92; P=0.001).<br><br>Diagnostic accuracy for any test combination (≥ 3 out of 5 positive):<br><br>Sens: 0.75 (95% CI 0.54-0.96)<br>Spec: 0.74 (95% CI 0.61-0.88)<br>LR+: 2.93 (95% CI 1.60-5.36)<br>LR-: 0.34 (95% CI 0.14-0.80)<br><br>Posttest probabilities:<br><br>≥ 3 out of 5 positive: LR+= 54.4%<br>< 3 out of 5 positive: LR- = 12.1%	Author's conclusion:<br><br>The single tests of painful arc, external rotation resistance test, and empty can provide the best diagnostic utility and reliability. The Neer test has clinical utility to screen for SAIS but has only fair reliability. Also of diagnostic utility is the use of the cut point of 3+/5 tests, with 3 or more tests positive of 5 useful in confirming SAIS, whereas less than 3 positive of the 5 tests is helpful in decreasing the likelihood of SAIS.

Formaterat: Avstånd Efter: 0 pt, Radavstånd: enkelt

Formaterad tabell

Formaterat: Avstånd Efter: 0 pt, Radavstånd: enkelt

Formaterat: Avstånd Efter: 0 pt, Radavstånd: enkelt

formaterade: Franska (standard)

Study reference	Study characteristics	Patient characteristics	Index test (test of interest)	Reference test	Follow-up	Outcome measures and effect size	Comments
	of the research supporting this article has or will confer a benefit on the authors or on any organization with which the authors are associated.		rotation by asking the patient to rotate the shoulder so that his/her thumb was pointing toward the floor. The examiner then applied a downward directed forced at the wrist while the patient attempted to resist. A positive test was considered if weakness was detected of the involved shoulder as compared bilaterally.<br><br>External rotation resistance test: performed by placing the arm at the patient's side and flexing their elbow to 90°. A medially directed force was exerted on the distal forearm to resist shoulder external rotation. A positive test was considered if weakness was detected of the involved shoulder as compared bilaterally.<br><br>Cut-off point(s):<br>An ROC curve analysis for each physical examination test was used to calculate the AUC, which represents the probability that the test can discriminate between healthy and disease states. The AUC values range from 0 to 1, with an AUC of 1 indicating 100% probability that a given test can discriminate between healthy and SAIS.<br><br>The cut point for discrimination was 3 positive tests out of 5.				

Formaterat: Avstånd Efter: 0 pt, Radavstånd: enkelt

Formaterad tabell

Formaterat: Avstånd Efter: 0 pt, Radavstånd: enkelt

Module 3

What is the value of ultrasonography versus MRI in patients who are suspected to have a partial thickness tear in the supraspinatus tendon?

Patients	Patients who are suspected to have a partial thickness tear in the supraspinatus tendon
Index test	Utrasound
Comparator test	MRI
Reference standard	Arthroscopy
Outcomes	Diagnostic accuracy: false negatives (FN), sensitivity, negative predictive value (NPV), false positives (FP), true positives (TP), true negatives (TN), specificity, positive predictive value (PPV), Fatty-infiltration, Percentage tear rupture, return to work or leisure
Timing and setting	Arthroscopy within 6 months preoperative US/MRI

Embase.com

No.	Query	Results
#17	#9 NOT (#14 OR #15 OR #16) = overig	182
#16	#9 AND (#12 OR #13) NOT (#14 OR #15) = observationeel	509
#15	#9 AND #11 NOT #14 = RCT	241
#14	#9 AND #10 = SR	66

Formaterat: Avstånd Efter: 0 pt, Radavstånd: enkelt
Formaterat: Avstånd Efter: 0 pt, Radavstånd: enkelt
Formaterat: Avstånd Efter: 0 pt, Radavstånd: enkelt
Formaterat: Avstånd Efter: 0 pt, Radavstånd: enkelt
Formaterat: Avstånd Efter: 0 pt, Radavstånd: enkelt

#13	'case control study'/de OR 'comparative study'/exp OR 'control group'/de OR 'controlled study'/de OR 'controlled clinical trial'/de OR 'crossover procedure'/de OR 'double blind procedure'/de OR 'phase 2 clinical trial'/de OR 'phase 3 clinical trial'/de OR 'phase 4 clinical trial'/de OR 'pretest posttest design'/de OR 'pretest posttest control group design'/de OR 'quasi experimental study'/de OR 'single blind procedure'/de OR 'triple blind procedure'/de OR (((control OR controlled) NEAR/6 trial):ti,ab,kw) OR (((control OR controlled) NEAR/6 (study OR studies)):ti,ab,kw) OR (((control OR controlled) NEAR/1 active):ti,ab,kw) OR 'open label*':ti,ab,kw OR (((double OR two OR three OR multi OR trial) NEAR/1 (arm OR arms)):ti,ab,kw) OR ((allocat* NEAR/10 (arm OR arms)):ti,ab,kw) OR placebo*:ti,ab,kw OR 'sham-control*':ti,ab,kw OR (((single OR double OR triple OR assessor) NEAR/1 (blind* OR masked)):ti,ab,kw) OR nonrandom*:ti,ab,kw OR 'non-random*':ti,ab,kw OR 'quasi-experiment*':ti,ab,kw OR crossover:ti,ab,kw OR 'cross over':ti,ab,kw OR 'parallel group*':ti,ab,kw OR 'factorial trial':ti,ab,kw OR ((phase NEAR/5 (study OR trial)):ti,ab,kw) OR ((case* NEAR/6 (matched OR control*)):ti,ab,kw) OR ((match* NEAR/6 (pair OR pairs OR cohort* OR control* OR group* OR healthy OR age OR sex OR gender OR patient* OR subject* OR participant*)):ti,ab,kw) OR ((propensity NEAR/6 (scor* OR match*)):ti,ab,kw) OR versus:ti OR vs:ti OR compar*:ti OR ((compar* NEAR/1 study):ti,ab,kw) OR (('major clinical study'/de OR 'clinical study'/de OR 'cohort analysis'/de OR 'observational study'/de OR 'cross-sectional study'/de OR 'multicenter study'/de OR 'correlational study'/de OR 'follow up'/de OR cohort*:ti,ab,kw OR 'follow up':ti,ab,kw OR followup:ti,ab,kw OR longitudinal*:ti,ab,kw OR prospective*:ti,ab,kw OR retrospective*:ti,ab,kw OR observational*:ti,ab,kw OR 'cross sectional*':ti,ab,kw OR cross?ectional*:ti,ab,kw OR multicent*:ti,ab,kw OR 'multi-cent*':ti,ab,kw OR consecutive*:ti,ab,kw) AND (group:ti,ab,kw OR groups:ti,ab,kw OR subgroup*:ti,ab,kw OR versus:ti,ab,kw OR vs:ti,ab,kw OR compar*:ti,ab,kw OR 'odds ratio*':ab OR 'relative odds':ab OR 'risk ratio*':ab OR 'relative risk*':ab OR 'rate ratio':ab OR aor:ab OR arr:ab OR rrr:ab OR ((('or' OR 'rr') NEAR/6 ci):ab)))	14157688
#12	'major clinical study'/de OR 'clinical study'/de OR 'case control study'/de OR 'family study'/de OR 'longitudinal study'/de OR 'retrospective study'/de OR 'prospective study'/de OR 'comparative study'/de OR 'cohort analysis'/de OR ((cohort NEAR/1 (study OR studies)):ab,ti) OR (('case control' NEAR/1 (study OR studies)):ab,ti) OR	7679776

Formaterat: Avstånd Efter: 0 pt, Radavstånd: enkelt

Formaterat: Avstånd Efter: 0 pt, Radavstånd: enkelt

	(('follow up' NEAR/1 (study OR studies)):ab,ti) OR (observational NEAR/1 (study OR studies)) OR ((epidemiologic NEAR/1 (study OR studies)):ab,ti) OR (('cross sectional' NEAR/1 (study OR studies)):ab,ti)	
#11	'clinical trial'/exp OR 'randomization'/exp OR 'single blind procedure'/exp OR 'double blind procedure'/exp OR 'crossover procedure'/exp OR 'placebo'/exp OR 'prospective study'/exp OR rct:ab,ti OR random*:ab,ti OR 'single blind':ab,ti OR 'randomised controlled trial':ab,ti OR 'randomized controlled trial'/exp OR placebo*:ab,ti	3806445
#10	'meta analysis'/exp OR 'meta analysis (topic)'/exp OR metaanaly*:ti,ab OR 'meta analy*':ti,ab OR metanaly*:ti,ab OR 'systematic review'/de OR 'cochrane database of systematic reviews'/jt OR prisma:ti,ab OR prospero:ti,ab OR (((systemati* OR scoping OR umbrella OR 'structured literature') NEAR/3 (review* OR overview*)):ti,ab) OR ((systemic* NEAR/1 review*):ti,ab) OR (((systemati* OR literature OR database* OR 'data base*') NEAR/10 search*):ti,ab) OR (((structured OR comprehensive* OR systemic*) NEAR/3 search*):ti,ab) OR (((literature NEAR/3 review*):ti,ab) AND (search*:ti,ab OR database*:ti,ab OR 'data base*':ti,ab)) OR (('data extraction':ti,ab OR 'data source*':ti,ab) AND 'study selection':ti,ab) OR ('search strategy':ti,ab AND 'selection criteria':ti,ab) OR ('data source*':ti,ab AND 'data synthesis':ti,ab) OR medline:ab OR pubmed:ab OR embase:ab OR cochrane:ab OR (((critical OR rapid) NEAR/2 (review* OR overview* OR synthes*)):ti) OR ((((critical* OR rapid*) NEAR/3 (review* OR overview* OR synthes*)):ab) AND (search*:ab OR database*:ab OR 'data base*':ab)) OR metasynthes*:ti,ab OR 'meta synthes*':ti,ab	933865
#9	#8 AND [2010-2023]/py	99
#8	#7 NOT ('conference abstract'/it OR 'editorial'/it OR 'letter'/it OR 'note'/it) NOT (('animal'/exp OR 'animal experiment'/exp OR 'animal model'/exp OR 'nonhuman'/exp) NOT 'human'/exp) NOT (('adolescent'/exp OR 'child'/exp OR adolescent*:ti,ab,kw OR child*:ti,ab,kw OR schoolchild*:ti,ab,kw OR infant*:ti,ab,kw OR girl*:ti,ab,kw OR boy*:ti,ab,kw OR teen:ti,ab,kw OR teens:ti,ab,kw OR teenager*:ti,ab,kw OR youth*:ti,ab,kw OR pediatr*:ti,ab,kw OR paediatr*:ti,ab,kw OR puber*:ti,ab,kw) NOT ('adult'/exp OR 'aged'/exp OR 'middle aged'/exp OR adult*:ti,ab,kw OR man:ti,ab,kw OR men:ti,ab,kw OR woman:ti,ab,kw OR women:ti,ab,kw))	1548

Formaterat: Avstånd Efter: 0 pt, Radavstånd: enkelt

Formaterat: Avstånd Efter: 0 pt, Radavstånd: enkelt

Formaterat: Avstånd Efter: 0 pt, Radavstånd: enkelt

Formaterat: Avstånd Efter: 0 pt, Radavstånd: enkelt

#7	#5 AND #6	1827
#6	'sensitivity and specificity'/de OR sensitivity:ab,ti OR sensitive:ab,ti OR specificity:ab,ti OR predict*:ab,ti OR 'roc curve':ab,ti OR 'receiver operator':ab,ti OR 'receiver operators':ab,ti OR likelihood:ab,ti OR 'diagnostic error'/exp OR 'diagnostic accuracy'/exp OR 'diagnostic test accuracy study'/exp OR 'inter observer':ab,ti OR 'intra observer':ab,ti OR interobserver:ab,ti OR intraobserver:ab,ti OR validity:ab,ti OR kappa:ab,ti OR reliability:ab,ti OR reproducibility:ab,ti OR ((test NEAR/2 're-test'):ab,ti) OR ((test NEAR/2 'retest'):ab,ti) OR 'reproducibility'/exp OR accuracy:ab,ti OR 'differential diagnosis'/exp OR 'validation study'/de OR 'measurement precision'/exp OR 'diagnostic value'/exp OR 'reliability'/exp OR 'predictive value'/exp OR ppv:ti,ab,kw OR npv:ti,ab,kw OR (((false OR true) NEAR/3 (negative OR positive)):ti,ab)	6682052
#5	#1 AND #4	5782
#4	#2 OR #3	2745662
#3	'nuclear magnetic resonance imaging'/exp OR 'mri scanner'/exp OR ('magnetic resonance':ti,ab,kw AND (image:ti,ab,kw OR images:ti,ab,kw OR imaging:ti,ab,kw)) OR mri:ti,ab,kw OR mris:ti,ab,kw OR nmr:ti,ab,kw OR mra:ti,ab,kw OR mras:ti,ab,kw OR zeugmatograph*:ti,ab,kw OR 'mr tomography':ti,ab,kw OR 'mr tomographies':ti,ab,kw OR 'mr tomographic':ti,ab,kw OR 'mr imag*':ti,ab,kw OR 'proton spin':ti,ab,kw OR ((magneti*:ti,ab,kw OR 'chemical shift':ti,ab,kw) AND imaging:ti,ab,kw) OR fmri:ti,ab,kw OR fmris:ti,ab,kw	1547891
#2	'ultrasound'/exp OR 'ultrasound scanner'/exp OR 'echography'/exp OR ultraso*:ti,ab,kw OR sonograph*:ti,ab,kw OR echograph*:ti,ab,kw OR sonogram*:ti,ab,kw	1405182
#1	'rotator cuff rupture'/exp OR ((('rotator cuff' OR supraspinatus OR subscapularis OR 'teres minor' OR 'glenoid labral' OR 'shoulder tendon*') NEAR/4 (ruptur* OR tear* OR tendinit* OR tendinos* OR tendinopath*)):ti,ab,kw)	14434

Ovid/Medline

#	Searches	Results
17	9 not (14 or 15 or 16) = overig	183

Formaterat: Avstånd Efter: 0 pt, Radavstånd: enkelt
Formaterat: Avstånd Efter: 0 pt, Radavstånd: enkelt
Formaterat: Avstånd Efter: 0 pt, Radavstånd: enkelt
Formaterat: Avstånd Efter: 0 pt, Radavstånd: enkelt
Formaterat: Avstånd Efter: 0 pt, Radavstånd: enkelt
Formaterat: Avstånd Efter: 0 pt, Radavstånd: enkelt
Formaterat: Avstånd Efter: 0 pt, Radavstånd: enkelt
Formaterat: Avstånd Efter: 0 pt, Radavstånd: enkelt
Formaterat: Avstånd Efter: 0 pt, Radavstånd: enkelt

16	(9 and (12 or 13)) not (14 or 15) = observationeel	554
15	(9 and 11) not 14 = RCT	61
14	9 and 10 = SR	48
13	Case-control Studies/ or clinical trial, phase ii/ or clinical trial, phase iii/ or clinical trial, phase iv/ or comparative study/ or control groups/ or controlled before-after studies/ or controlled clinical trial/ or double-blind method/ or historically controlled study/ or matched-pair analysis/ or single-blind method/ or (((control or controlled) adj6 (study or studies or trial)) or (compar* adj (study or studies)) or ((control or controlled) adj1 active) or "open label*" or ((double or two or three or multi or trial) adj (arm or arms)) or (allocat* adj10 (arm or arms)) or placebo* or "sham-control*" or ((single or double or triple or assessor) adj1 (blind* or masked)) or nonrandom* or "non-random*" or "quasi-experiment*" or "parallel group*" or "factorial trial" or "pretest posttest" or (phase adj5 (study or trial)) or (case* adj6 (matched or control*)) or (match* adj6 (pair or pairs or cohort* or control* or group* or healthy or age or sex or gender or patient* or subject* or participant*)) or (propensity adj6 (scor* or match*))).ti,ab,kf. or (confounding adj6 adjust*).ti,ab. or (versus or vs or compar*).ti. or ((exp cohort studies/ or epidemiologic studies/ or multicenter study/ or observational study/ or seroepidemiologic studies/ or (cohort* or 'follow up' or followup or longitudinal* or prospective* or retrospective* or observational* or multicent* or 'multi-cent*' or consecutive*).ti,ab,kf.) and ((group or groups or subgroup* or versus or vs or compar*).ti,ab,kf. or ('odds ratio*' or 'relative odds' or 'risk ratio*' or 'relative risk*' or aor or arr or rrr).ab. or (("OR" or "RR") adj6 CI).ab.))	5439931
12	Epidemiologic studies/ or case control studies/ or exp cohort studies/ or Controlled Before-After Studies/ or Case control.tw. or cohort.tw. or Cohort analy$.tw. or (Follow up adj (study or studies)).tw. or (observational adj (study or studies)).tw. or Longitudinal.tw. or Retrospective*.tw. or prospective*.tw. or consecutive*.tw. or Cross sectional.tw. or Cross-sectional studies/ or historically controlled study/ or interrupted time series analysis/ [Onder exp cohort studies vallen ook longitudinale, prospectieve en retrospectieve studies]	4455625
11	exp clinical trial/ or randomized controlled trial/ or exp clinical trials as topic/ or randomized controlled trials as topic/ or Random Allocation/ or Double-Blind Method/ or Single-Blind Method/ or (clinical trial, phase i or clinical trial, phase ii	2596278

	or clinical trial, phase iii or clinical trial, phase iv or controlled clinical trial or randomized controlled trial or multicenter study or clinical trial).pt. or random*.ti,ab. or (clinic* adj trial*).tw. or ((singl* or doubl* or treb* or tripl*) adj (blind$3 or mask$3)).tw. or Placebos/ or placebo*.tw.	
10	meta-analysis/ or meta-analysis as topic/ or (metaanaly* or meta-analy* or metanaly*).ti,ab,kf. or systematic review/ or cochrane.jw. or (prisma or prospero).ti,ab,kf. or ((systemati* or scoping or umbrella or "structured literature") adj3 (review* or overview*)).ti,ab,kf. or (systemic* adj1 review*).ti,ab,kf. or ((systemati* or literature or database* or data-base*) adj10 search*).ti,ab,kf. or ((structured or comprehensive* or systemic*) adj3 search*).ti,ab,kf. or ((literature adj3 review*) and (search* or database* or data-base*)).ti,ab,kf. or (("data extraction" or "data source*") and "study selection").ti,ab,kf. or ("search strategy" and "selection criteria").ti,ab,kf. or ("data source*" and "data synthesis").ti,ab,kf. or (medline or pubmed or embase or cochrane).ab. or ((critical or rapid) adj2 (review* or overview* or synthes*)).ti. or (((critical* or rapid*) adj3 (review* or overview* or synthes*)) and (search* or database* or data-base*)).ab. or (metasynthes* or meta-synthes*).ti,ab,kf.	672666
9	limit 8 to yr="2010 -Current"	846
8	7 not (comment/ or editorial/ or letter/) not ((exp animals/ or exp models, animal/) not humans/) not ((Adolescent/ or Child/ or Infant/ or adolescen*.ti,ab,kf. or child*.ti,ab,kf. or schoolchild*.ti,ab,kf. or infant*.ti,ab,kf. or girl*.ti,ab,kf. or boy*.ti,ab,kf. or teen.ti,ab,kf. or teens.ti,ab,kf. or teenager*.ti,ab,kf. or youth*.ti,ab,kf. or pediatr*.ti,ab,kf. or paediatr*.ti,ab,kf. or puber*.ti,ab,kf.) not (Adult/ or adult*.ti,ab,kf. or man.ti,ab,kf. or men.ti,ab,kf. or woman.ti,ab,kf. or women.ti,ab,kf.))	1314
7	5 and 6	1348
6	exp "Sensitivity and Specificity"/ or (sensitivity or sensitive or specificity).ti,ab. or (predict* or ROC-curve or receiver-operator*).ti,ab. or (likelihood or LR*).ti,ab. or exp Diagnostic Errors/ or (inter-observer or intra-observer or interobserver or intraobserver or validity or kappa or reliability).ti,ab. or reproducibility.ti,ab. or (test adj2 (re-test or retest)).ti,ab. or "Reproducibility of Results"/ or accuracy.ti,ab. or Diagnosis, Differential/ or Validation Study/ or ((false or true) adj3 (negative or positive)).ti,ab.	5335333

Formaterat: Avstånd Efter: 0 pt, Radavstånd: enkelt

Formaterat: Avstånd Efter: 0 pt, Radavstånd: enkelt

Formaterat: Avstånd Efter: 0 pt, Radavstånd: enkelt

Formaterat: Avstånd Efter: 0 pt, Radavstånd: enkelt

Formaterat: Avstånd Efter: 0 pt, Radavstånd: enkelt

5	1 and 4	4294
4	2 or 3	1620282
3	exp magnetic resonance imaging/ or ("magnetic resonance" and (image or images or imaging)).ti,ab,kf. or mri.ti,ab,kf. or mris.ti,ab,kf. or nmr.ti,ab,kf. or mra.ti,ab,kf. or mras.ti,ab,kf. or zeugmatograph*.ti,ab,kf. or "mr tomography".ti,ab,kf. or "mr tomographies".ti,ab,kf. or "mr tomographic".ti,ab,kf. or 'mr imag*'.ti,ab,kf. or "proton spin".ti,ab,kf. or ((magneti* or "chemical shift") and imaging).ti,ab,kf. or fmri.ti,ab,kf. or fmris.ti,ab,kf.	948288
2	exp Ultrasonography/ or exp Ultrasonics/ or ultraso*.ti,ab,kf. or sonograph*.ti,ab,kf. or echograph*.ti,ab,kf. or sonogram*.ti,ab,kf.	745809
1	exp Rotator Cuff Injuries/ or (exp Rotator Cuff/ and (exp Rupture/ or exp Tears/)) or (('rotator cuff' or supraspinatus or subscapularis or 'teres minor' or 'glenoid labral' or 'shoulder tendon*') adj4 (ruptur* or tear* or tendinit* or tendinos* or tendinopath*)).ti,ab,kf.	12436

Formaterat: Avstånd Efter: 0 pt, Radavstånd: enkelt

Formaterat: Avstånd Efter: 0 pt, Radavstånd: enkelt

Formaterat: Avstånd Efter: 0 pt, Radavstånd: enkelt

Formaterat: Avstånd Efter: 0 pt, Radavstånd: enkelt

Formaterat: Avstånd Efter: 0 pt, Radavstånd: enkelt

Evidence table module 3

<table>
<tr><th>Study reference</th><th>Study characteristics</th><th>Patient characteristics</th><th>Index test
(test of interest)</th><th>Reference test</th><th>Follow-up</th><th>Outcome measures and effect size</th><th>Comments</th></tr>
<tr>
<td>Farooqi, 2021

PS., study characteristics and results are extracted from the SR (unless stated otherwise: * indicates data was extracted from individual studies)</td>
<td>SR and meta-analysis

Literature search up to January 1, 2010, and April 1, 2020

Five studies of the 23 included studies in de SR compared the diagnostic capability of US and
MRI for partial supraspinatus tears using arthroscopy as the reference standard and were included:

A: Abd-ElGawad (2013)2
B: Day (2016)10
C: Elmorsy (2017)15
D: Rutten (2010)48
E: Sabharwal (2019)50

<u>Study design</u>: cohort, case-control [prospective / retrospective]</td>
<td>Inclusion criteria SR: studies that evaluated the diagnostic accuracy of US in rotator cuff tears utilizing arthroscopy as the reference standard

Exclusion criteria SR: review articles, meta-analyses, systematic reviews, case reports, cadaveric studies, non-English text, studies with <10 patients, studies including massive tears without reporting diagnostic data for specific tendons, and studies lacking diagnostic outcome data.

<u>Important patient characteristics</u>:</td>
<td>Index (1) and comparator tests (2)* and cut-off point(s):

A-1: US (GE Logiq 5, 12 MHz); cut-off NA [operator: general radiologist]
A-2: MRI, cut-off NA MRI examinations were performed with a 1TMR Imagingunit(Intera, Philips Medical Systems, NeberlandB.V) and one surface coil was used

B-1: US; cut-off NA [operator: surgeon]
B-1: MRI, cut-off NA (MRI details/transducer not reported)

C-1: US; cut-off NA [operator: MSK radiologist]
C-2: MRI, cut-off NA MRI scans were performed using 1.5 or 3 Tesla MRI scanners

D-1: US; cut-off NA [operator: MSK radiologist]</td>
<td>Reference test and cut-off point(s):

A: arthroscopy; cut-off NR
B: arthroscopy; cut-off NR
C: arthroscopy; cut-off NR
D: arthroscopy; cut-off NR
E: arthroscopy; cut-off NR

Prevalence partial thickness cuff tear (PPT) (%)*
[based on refence test at specified cut-off point]
A: 13 (32.5%)
B: 7 (36.8%)
C: 13 (10.4%)
D: 9 (18.4%)
E: 20 (33.3%)

For how many participants were no complete outcome data available?
N (%)
A: NR
B: NR
C: NR
D: NR
E: NR</td>
<td>Endpoint of follow-up:
A: NR
B: NR
C: NR
D: NR
E: NR</td>
<td><u>TP/FP/FN/TN*:</u>
A:
<table><tr><td></td><td>PTT+</td><td>PTT-</td><td></td></tr><tr><td>US+</td><td>TP: 12</td><td>FP: 3</td><td>15</td></tr><tr><td>US-</td><td>FN: 1</td><td>TN: 24</td><td>25</td></tr><tr><td></td><td>13</td><td>27</td><td>40</td></tr></table>
<table><tr><td></td><td>PTT+</td><td>PTT-</td><td></td></tr><tr><td>MRI+</td><td>TP: 11</td><td>FP: 2</td><td>13</td></tr><tr><td>MRI-</td><td>FN: 2</td><td>TN: 25</td><td>27</td></tr><tr><td></td><td>13</td><td>27</td><td>40</td></tr></table>
B:
<table><tr><td></td><td>PTT+</td><td>PTT-</td><td></td></tr><tr><td>US+</td><td>TP: 5</td><td>FP: 2</td><td>7</td></tr><tr><td>US-</td><td>FN: 2</td><td>TN: 10</td><td>12</td></tr><tr><td></td><td>7</td><td>12</td><td>19</td></tr></table>
<table><tr><td></td><td>PTT+</td><td>PTT-</td><td></td></tr><tr><td>MRI+</td><td>TP: 6</td><td>FP: 2</td><td>7</td></tr><tr><td>MRI-</td><td>FN: 1</td><td>TN: 11</td><td>12</td></tr><tr><td></td><td>7</td><td>12</td><td>19</td></tr></table>
C:
<table><tr><td></td><td>PTT+</td><td>PTT-</td><td></td></tr></table></td>
<td><u>Study aim:</u> To evaluate the diagnostic accuracy of US for partial- and full-thickness rotator cuff tears and biceps tendon tears, compare diagnostic values with those of magnetic resonance imaging (MRI) using arthroscopy as the reference standard, assess longitudinal improvements in accuracy, and compare diagnostic values from operators with different training backgrounds.

<u>Study quality (ROB):</u> The quality and risk of bias for each included study was assessed by 2 authors (A.S.F. and A.L.) using the Quality Assessment of Diagnostic Accuracy Studies–2 (QUADAS-2) Tool across 4 domains (For each domain, studies were assigned a score of low (low risk of bias or low concern regarding applicability), unclear, or high (high risk of bias or high concern regarding applicability).):

Patient selection
A: high
B: low</td>
</tr>
</table>

Formaterat: Avstånd Efter: 0 pt, Radavstånd: enkelt
Formaterat: Avstånd Efter: 0 pt, Radavstånd: enkelt
Formaterat: Avstånd Efter: 0 pt, Radavstånd: enkelt
Formaterat: Avstånd Efter: 0 pt, Radavstånd: enkelt
Formaterat: Avstånd Efter: 0 pt, Radavstånd: enkelt
Formaterat: Avstånd Efter: 0 pt, Radavstånd: enkelt
Formaterat: Avstånd Efter: 0 pt, Radavstånd: enkelt
Formaterat: Avstånd Efter: 0 pt, Radavstånd: enkelt
Formaterat: Avstånd Efter: 0 pt, Radavstånd: enkelt
Formaterat: Avstånd Efter: 0 pt, Radavstånd: enkelt
Formaterat: Avstånd Efter: 0 pt, Radavstånd: enkelt
Formaterat: Avstånd Efter: 0 pt, Radavstånd: enkelt
Formaterat: Avstånd Efter: 0 pt, Radavstånd: enkelt
Formaterat: Avstånd Efter: 0 pt, Radavstånd: enkelt
Formaterat: Avstånd Efter: 0 pt, Radavstånd: enkelt
Formaterat: Avstånd Efter: 0 pt, Radavstånd: enkelt
Formaterat: Avstånd Efter: 0 pt, Radavstånd: enkelt
Formaterat: Avstånd Efter: 0 pt, Radavstånd: enkelt

<table>
<tr>
<td></td>
<td>A: Abd-ElGawad (2013)2
B: Day (2016)10
C: Elmorsy (2017)15
D: Rutten (2010)48
E: Sabharwal (2019)50

<u>Setting and Country</u>: USA

<u>Source of funding and conflicts of interest:</u> One or more of the authors has declared the following potential conflict of interest or source of funding: X.L. has received consulting fees and royalties from FH Ortho. J.D.K. has received consulting fees from Flexion and royalties from SLACK and Springer. R.L.P. has received grant/educational support from Arthrex. AOSSM checks author disclosures against the Open Payments Database (OPD). AOSSM has not</td>
<td><u>N, mean age</u>
A: 40, 55 yrs
B: 19, 55 yrs
C: 125, 52 yrs
D: 48, 68 yrs
E: 60, 45 yrs</td>
<td>D-2: MRI, cut-off NA
All MRI examinations were performed with an 1.5-T MR system (Signa Horizon, GE, 's-Hertogenbosch, The Netherlands).

E-1: US (GE Voluson P8, 7-12 MHz); cut-off NA [operator: general radiologist]
E-2: MRI, cut-off NA (MRI details/transducer not reported)</td>
<td>Reasons for incomplete outcome data described?
A: NR
B: NR
C: NR
D: NR
E: NR</td>
<td></td>
<td>
<table>
<tr><td>US +</td><td>TP: NR</td><td>FP: NR</td><td></td></tr>
<tr><td>US -</td><td>FN: NR</td><td>TN: NR</td><td></td></tr>
<tr><td></td><td></td><td></td><td>125</td></tr>
</table>
<table>
<tr><td></td><td>PTT +</td><td>PTT -</td><td></td></tr>
<tr><td>MRI +</td><td>TP: NR</td><td>FP: NR</td><td></td></tr>
<tr><td>MRI -</td><td>FN: NR</td><td>TN: NR</td><td></td></tr>
<tr><td></td><td></td><td></td><td>125</td></tr>
</table>
D:
<table>
<tr><td></td><td>PTT +</td><td>PTT -</td><td></td></tr>
<tr><td>US +</td><td>TP: 8</td><td>FP: 22</td><td>20</td></tr>
<tr><td>US -</td><td>FN: 1</td><td>TN: 47</td><td>48</td></tr>
<tr><td></td><td>9</td><td>69</td><td>68</td></tr>
</table>
<table>
<tr><td></td><td>PTT +</td><td>PTT -</td><td></td></tr>
<tr><td>MRI +</td><td>TP: 6</td><td>FP: 8</td><td>14</td></tr>
<tr><td>MRI -</td><td>FN: 3</td><td>TN: 51</td><td>54</td></tr>
<tr><td></td><td>9</td><td>59</td><td>68</td></tr>
</table>
E:
<table>
<tr><td></td><td>PTT +</td><td>PTT -</td><td></td></tr>
<tr><td>US +</td><td>TP: 19</td><td>FP: 3</td><td>22</td></tr>
<tr><td>US -</td><td>FN: 1</td><td>TN: 37</td><td>38</td></tr>
<tr><td></td><td>20</td><td>40</td><td>60</td></tr>
</table>
</td>
<td>C: low
D: low
E: low

Index test – US
A: unclear
B: low
C: unclear
D: low
E: low

Index test – MRI
A: unclear
B: low
C: unclear
D: NA
E: low

Reference standard
A: unclear
B: low
C: unclear
D: low
E: low

Flow and Timing
A: high
B: unclear
C: high
D: unclear
E: unclear

<u>Place of the index test in the clinical pathway</u>: add-on (MRI after US)

<u>Choice of cut-off point</u>: visual via operation

<u>Brief description of author's discussion/ conclusion:</u> "This study demonstrated that US is highly sensitive and specific in the diagnosis of supraspinatus tears and</td>
</tr>
</table>

Formaterat: Avstånd Efter: 0 pt, Radavstånd: enkelt
Formaterat: Avstånd Efter: 0 pt, Radavstånd: enkelt
Formaterat: Avstånd Efter: 0 pt, Radavstånd: enkelt
Formaterat: Avstånd Efter: 0 pt, Radavstånd: enkelt
Formaterat: Avstånd Efter: 0 pt, Radavstånd: enkelt
Formaterat: Avstånd Efter: 0 pt, Radavstånd: enkelt
Formaterat: Avstånd Efter: 0 pt, Radavstånd: enkelt
Formaterat: Avstånd Efter: 0 pt, Radavstånd: enkelt
Formaterat: Avstånd Efter: 0 pt, Radavstånd: enkelt
Formaterat: Avstånd Efter: 0 pt, Radavstånd: enkelt
Formaterat: Avstånd Efter: 0 pt, Radavstånd: enkelt
Formaterat: Avstånd Efter: 0 pt, Radavstånd: enkelt
Formaterat: Avstånd Efter: 0 pt, Radavstånd: enkelt
Formaterat: Avstånd Efter: 0 pt, Radavstånd: enkelt
Formaterat: Avstånd Efter: 0 pt, Radavstånd: enkelt
Formaterat: Avstånd Efter: 0 pt, Radavstånd: enkelt
Formaterat: Avstånd Efter: 0 pt, Radavstånd: enkelt
Formaterat: Avstånd Efter: 0 pt, Radavstånd: enkelt
Formaterat: Avstånd Efter: 0 pt, Radavstånd: enkelt

<table>
<tr><th></th><th></th><th></th><th></th><th></th><th></th><th></th><th></th><th></th></tr>
<tr>
<td></td>
<td>conducted an independent investigation on the OPD and disclaims any liability or responsibility relating thereto.</td>
<td></td>
<td></td>
<td></td>
<td></td>
<td></td>
<td>
<table>
<tr><td></td><td>PT T+</td><td>PT T-</td><td></td></tr>
<tr><td>MR I+</td><td>TP : 17</td><td>FP : 3</td><td>2 0</td></tr>
<tr><td>MR I-</td><td>FN : 3</td><td>TN : 37</td><td>4 0</td></tr>
<tr><td></td><td>20</td><td>40</td><td>6 0</td></tr>
</table>

<u>Sensitivity/ specificity/ diagnostic accuracy-1/2*</u>
A-1: 92.3%/ 92.6%/ 92.5%
A-2: 84.6%/ 92.6%/ 90%

B-1: 0.71/1.00/ 74%
B-2: 1.00/ 1.00/ 100%
But for all tears, no distinction in partial/ full tears → own calculations

C-1: 23%/ 90.1%
C-2: 54.1% (P=0.333) / 72.6% (P=0.0008)

D-1: 89% [52%-100%]/ 80% [67%-89%]/ 81% [70%-89%]
D-2: 67% [30%-93%]/ 86% [75%-94%]/ 84% [73%-92%]

E-1: 95.0%/ 92.5%/ 93.3%
E-2: 85.0%/ 92.5%/ 90.0%

US median sensitivity 0.89
US median specificity 0.89
MRI median sensitivity 0.85
MRI median specificity 0.87

Pooled characteristics:
sensitivity = –2.36 [95% CI, –21.51 to 16.79], P = .81
specificity = 4.30 [95% CI, –7.06 to 15.66], P = .46
diagnostic accuracy = 5.25 [95% CI, –1.60 to 12.10], P = .13
</td>
<td>is more accurate in the diagnosis of full-thickness tears
as compared to partial-thickness tears.”

“[..] US demonstrated a lower median sensitivity and specificity for partial thickness tears, at 0.65 and 0.86, respectively. In support
of these findings, 1 study reported that diagnostic accuracy increased with tear size for full-thickness supraspinatus
tears.9 One proposed contributing factor to the lower US sensitivity realized for partial-thickness tears may be the
variable echogenicity of synovial proliferation, granulation,
and scar tissue formation surrounding a partial tear, thus
impeding clear tissue differentiaiton.40 Because of the
lower US sensitivity for partial-thickness tears, it has been
recommended to follow a negative US examination with an
MRI in patients who do not experience symptomatic relief
following conservative treatment.53 We propose a similar diagnostic US screening algorithm shown in Figure 5.”</td>
</tr>
</table>

Formaterat: Avstånd Efter: 0 pt, Radavstånd: enkelt

Formaterat: Avstånd Efter: 0 pt, Radavstånd: enkelt

Formaterat: Avstånd Efter: 0 pt, Radavstånd: enkelt

Formaterat: Avstånd Efter: 0 pt, Radavstånd: enkelt

Formaterat: Avstånd Efter: 0 pt, Radavstånd: enkelt

						Heterogeneity (reasons):<br>No significant heterogeneity was observed among the studies<br>in US and MRI diagnostic outcomes when evaluating partial-thickness<br>tears (I^2 = 0%-41%; P = 0.15-0.50)<br><br>PPV/NPV-1/2*<br>**A-1**: NR (own calculations)<br>**A-2**: NR (own calculations)<br><br>**B-1**: 1.00/ 0.29<br>**B-2**: 1.00/ 1.00<br><br>**C-1**: 21.4%/ 90.9%<br>**C-2**: 30.9% (p=0.73) / 87.5% (p=0.48)<br><br>**D-1**: 40% [19%-64%]/ 98% [89%-100%]<br>**D-2**: 43% [18%-71%]/ 94% [85%-99%]<br><br>**E-1**: 86.4%/ 97.4%<br>**E-2**: 85.0%/ 92.5%	

*comparator test equals the C of the PICO; two or more index/ comparator tests may be compared; note that a comparator test is not the same as a reference test (golden standard)

Formaterat: Avstånd Efter: 0 pt, Radavstånd: enkelt

Study reference	Study characteristics	Patient characteristics	Index test (test of interest)	Reference test	Follow-up	Outcome measures and effect size	Comments
Li, 2023	Type of study[1]: retrospective study; to	Inclusion criteria:<br>: 1)	Index test 1: US<br>US and PUSB examinations were performed using	Reference test[3]: shoulder arthroscopy performed by an associate chief	Time between the index test and reference test: NR	TP/FP/FN/TN*:	Author conclusion: In conclusion, PUSB is highly accurate, sensitive,

Formaterat: Avstånd Efter: 0 pt, Radavstånd: enkelt

[1] In geval van een case-control design moeten de patiëntkarakteristieken per groep (cases en controls) worden uitgewerkt. NB; case control studies zullen de accuratesse overschatten (Lijmer et al., 1999)

[3] De referentiestandaard is de test waarmee definitief wordt aangetoond of iemand al dan niet ziek is. Idealiter is de referentiestandaard de Gouden standaard (100% sensitief en 100% specifiek). Let op! dit is niet de "comparison test/index 2".

<table>
<tr><th>Study reference</th><th>Study characteristics</th><th>Patient characteristics</th><th>Index test
(test of interest)</th><th>Reference test</th><th>Follow-up</th><th>Outcome measures and effect size</th><th>Comments</th></tr>
<tr>
<td></td>
<td>explore the feasibility and diagnostic value of percutaneous US-guided subacromial bursography (PUSB) in evaluating rotator cuff tears

Setting and country: single-centre, China

Funding and conflicts of interest:
Funding: NR;
Conflict of interest: none</td>
<td>having complete clinical, US, MRI and PUSB data; 2) underwent shoulder arthroscopy surgery.

Exclusion criteria: 1) having incomplete imaging data; 2) patients unsuitable for PUSB examination; 3) patients who have already undergone previous surgery on the shoulder area

N=Seventy-eight patients with shoulder arthroscopic surgery and images of conventional US,
MRI and PUSB examined in our</td>
<td>SIEMENS ACUSON Sequoia (Siemens Medical Solu_tions, USA). US examination was performed with a 6-18 MHz linear array probe (18L6). The patient was seated
and facing the operator, who performs the procedure according to the shoulder US technical guidelines recommended by the European Society of Musculoskeletal Radiology [15]. The biceps long-head tendon, subscapularis tendon, supraspinatus tendon, infraspinatus and teres minor tendons were examined successively. Transverse and longitudinal images were performed, and the dynamic
and static images were retained.

Index test 2: a 4-10 MHz linear array probe (10L4) was used to
perform PUSB. The tip of the needle was directed into
the subacromial bursa and the contrast agent was slowly</td>
<td>physician with more than 10 years of shoulder arthroscopy experience

Cut-off point(s): diagnostic criteria

the types of rotator cuff tears were classified
as full-thickness tears, partial-thickness tears and no tears according to whether there were any rotator cuff defects and the location and size of defects.</td>
<td>For how many participants were no complete outcome data available?
N (%): NR, it was an exclusion criterium

Reasons for incomplete outcome data described?
NR, it was an exclusion criterium</td>
<td>
<table>
<tr><td></td><td>PTT +</td><td>PTT -</td><td></td></tr>
<tr><td>US +</td><td>TP: 27</td><td>FP: 4</td><td>31</td></tr>
<tr><td>US -</td><td>FN: 15</td><td>TN: 32</td><td>47</td></tr>
<tr><td></td><td>42</td><td>36</td><td>78</td></tr>
</table>
<table>
<tr><td></td><td>PTT +</td><td>PTT -</td><td></td></tr>
<tr><td>PUSB +</td><td>TP: 40</td><td>FP: 1</td><td>41</td></tr>
<tr><td>PUSB -</td><td>FN: 2</td><td>TN: 35</td><td>37</td></tr>
<tr><td></td><td>42</td><td>36</td><td>78</td></tr>
</table>
<table>
<tr><td></td><td>PTT +</td><td>PTT -</td><td></td></tr>
<tr><td>MRI +</td><td>TP: 32</td><td>FP: 4</td><td>36</td></tr>
<tr><td>MRI -</td><td>FN: 10</td><td>TN: 32</td><td>42</td></tr>
<tr><td></td><td>42</td><td>36</td><td>78</td></tr>
</table>
<u>Sensitivity</u>:
US: 64.3 (49.17,79.40)
PUBS: 95.2 (88.52,101.95)
MRI: 76.2 (62.76,89.62)

<u>Specificity:</u>
US: 88.9 (78.10,99.67)
PUBS: 97.2 (91.58,102.86)
MRI: 88.9 (78.10,99.67)

<u>PPV:</u>
US: 87.1 (74.60,99.60)
</td>
<td>and specific for the diagnosis of rotator cuff tears. At the
same time, PUSB can be used to dynamically observe the
rotator cuff tears in a timely manner. When patients have
MRI contraindications or MRI cannot accurately deter_mine the types of rotator cuff tears, PUSB can be used
for auxiliary diagnosis with decreased cost and increased
efficiency, making this method a good choice for patients
in urgent need of surgery.</td>
</tr>
</table>

[4] Beschrijf de statistische parameters voor de vergelijking van de indextest(en) met de referentietest, en voor de vergelijking tussen de indextesten onderling (als er twee of meer indextesten worden vergeleken).

Formaterat: Avstånd Efter: 0 pt, Radavstånd: enkelt
Formaterat: Avstånd Efter: 0 pt, Radavstånd: enkelt
Formaterat: Avstånd Efter: 0 pt, Radavstånd: enkelt
Formaterat: Avstånd Efter: 0 pt, Radavstånd: enkelt
Formaterat: Avstånd Efter: 0 pt, Radavstånd: enkelt
Formaterat: Avstånd Efter: 0 pt, Radavstånd: enkelt
Formaterat: Avstånd Efter: 0 pt, Radavstånd: enkelt
Formaterat: Avstånd Efter: 0 pt, Radavstånd: enkelt
Formaterat: Avstånd Efter: 0 pt, Radavstånd: enkelt
Formaterat: Avstånd Efter: 0 pt, Radavstånd: enkelt
Formaterat: Avstånd Efter: 0 pt, Radavstånd: enkelt
Formaterat: Avstånd Efter: 0 pt, Radavstånd: enkelt

Study reference	Study characteristics	Patient characteristics	Index test (test of interest)	Reference test	Follow-up	Outcome measures and effect size	Comments
		department between July 2019 to October 2021.<br><br>Prevalence: 42 cases of PTTs (48%)<br><br>Mean age (range): mean age 53.9±9.1 years; age range, 31-70 years<br><br>Sex: 32 males, 46 females	injected. At the same time, the probe was rotated to observe the distribution of contrast agent in the bursa and tendon. Typical images were captured and stored during the inspection for recording and analysis. After the examination, the puncture site was disinfected and covered with a sterile dressing.<br><br>Comparator test[2]: MRI<br>MRI was performed with 1.5 T superconducting MRI equipment from the German Siemens Magnetom Avanto, equipped with a special coil for the shoulder joint. For coronal section scanning, the scanning plane was perpendicular to the glenoid cavity and ranged from the acromion to subscapular humerus with a fast-spin echo T2-weighted sequence (TR/TE=2200 ms/84 ms) and a			PUBS: 97.6 (92.63,102.49)<br>MRI: 88.9 (78.10,99.67)<br><br><u>NPV:</u><br>US: 68.1 (54.25,81.92)<br>PUBS: 94.6 (86,95,102.24)<br>MRI: 76.2 (62.76,89.62)	

[2] Comparator test is vergelijkbaar met de C uit de PICO van een interventievraag. Er kunnen ook meerdere tests worden vergeleken. Voeg die toe als comparator test 2 etc. Let op: de comparator test kan <u>nooit</u> de referentiestandaard zijn.

Study reference	Study characteristics	Patient characteristics	Index test (test of interest)	Reference test	Follow-up	Outcome measures and effect size	Comments
			spin echo T1 weighted sequence (TR/TE=450 ms/16 ms). For oblique coronal scanning, the scanning plane was parallel to the long axis of the supraspinatus muscle and ranged from the outer end of the clavicle to the ac_romion with rapid spin echo T2-weighted imaging (TR/ TE= 2370 ms/39 ms). The scanning parameters were as follows: a FOV = 20 cm×20 cm; a matrix =257×192; a layer thickness =4 mm; and a layer spacing = 4.8 mm.<br><br>Cut-off point(s): Diagnostic criteria<br><br>MRI: (1) Full-thickness tear: the supraspinatus tendon was thickened and twisted, with a high signal involving the whole layer. (2) Partial-thickness tear: the supraspinatus tendon was irregular in shape, with a focal high signal, and the whole layer is not involved [16,17].				

Study reference	Study characteristics	Patient characteristics	Index test (test of interest)	Reference test	Follow-up	Outcome measures and effect size	Comments
			US: (1) Full-thickness tear: (i) a hypoechoic defect extends from the bursal to the articular sides; (ii) local defects involving both the bursal and articular sides in the short-axis and long-axis views; and (iii) the rotatorcuff not visible due to extensive full-thickness tears and retraction below the acromion. (2) Partial-thickness tear: (i) an obvious hypoechoic defect area or a discontinuous area on the bursal or articular sides of the tendon is pre_sent; (ii) focal hypoechoic defects within the tendon are seen in the longitudinal and transverse planes [18]. (3) No tear (NTs): a normal subacromial-subdeltoid bursa (SASD) appeared as a hypoechoic line between two hy_perechoic planes, with total thickness of less than 2 mm [12].<br><br>PUSB: (1) Full-thickness tear: the contrast agent leaks from the defect area of the bursal side through the supraspinatus into the articular side. (2) Partial-thickness tear: for the part of bursal-side tears, PUSB shows that the contrast				

Study reference	Study characteristics	Patient characteristics	Index test (test of interest)	Reference test	Follow-up	Outcome measures and effect size	Comments
			agent filled the bursal-side tear part and the contrast agent flows from the subacromial bursa to the bursal-side tears area in the PUSB dynamic imaging. For the intratendinous or articular side partial-thickness tears, the contrast agent can be observed in tendons or from tendons to the articular side by injecting it into the area of the suspected tendon lesion directly. (3) No tear: the contrast agent is scattered only in the subacromial bursa, outlines the regular surface of the rotator cuff, and does not leak into the rotator cuff.				
McGuire, 2023	Type of study[4]: retrospective, quantitative study with data collected<br><br>from participants'	Inclusion criteria:<br><br>NR<br><br>Exclusion criteria: those	Index test: US<br><br>Ultrasounds were performed by<br><br>general sonographers. Ultrasound machines involved in the study	Reference test[6]: shoulder arthroscopic repair<br><br>Cut-off point(s): NR	Time between the index test and reference test: NR<br><br>For how many participants were no complete outcome data available?<br><br>N (%): NR	TP/FP/FN/TN*:<br><br>**Partial thickness**<br><br>**tear <5 mm**	Author conclusion:<br>Advances in USS shoulder imaging due to increased spatial resolution<br><br>has revolutionised shoulder imaging over the last 20 years. This study

[4] In geval van een case-control design moeten de patiëntkarakteristieken per groep (cases en controls) worden uitgewerkt. NB; case control studies zullen de accuratesse overschatten (Lijmer et al., 1999)
[6] De referentiestandaard is de test waarmee definitief wordt aangetoond of iemand al dan niet ziek is. Idealiter is de referentiestandaard de Gouden standaard (100% sensitief en 100% specifiek). Let op! dit is niet de "comparison test/index 2".
[4] Beschrijf de statistische parameters voor de vergelijking van de indextest(en) met de referentietest, en voor de vergelijking tussen de indextesten onderling (als er twee of meer indextesten worden vergeleken).

<table>
<tr><th>Study reference</th><th>Study characteristics</th><th>Patient characteristics</th><th>Index test
(test of interest)</th><th>Reference test</th><th>Follow-up</th><th>Outcome measures and effect size</th><th>Comments</th></tr>
<tr>
<td></td>
<td>medical records; to

compare the diagnostic sensitivity of USS and MRI in the pre-operative diagnosis

of partial thickness tears (PTT) and full thickness tears (FTT) of supraspinatus tendons

compared to gold-standard shoulder arthroscopy to assess if differences in

detection rates exist.</td>
<td>who had a longer than 3-month period

between diagnosis and arthroscope, and whose pathology was unrelated

to the supraspinatus tendon and participants aged less than 18 years.

N= One hundred and three participants who had arthroscopic shoulder

repair had their data reviewed comparing the</td>
<td>included Philips Epiq5, Canon Aplio 500, and General Electric Logiq E9.

These machines are equipped with high frequency linear probes in the

range of 10–15 MHz appropriate for musculoskeletal scanning.

Comparator test[5]: MRI

The MRI machines in this study included a Philips Ingenia 1.5 T, Philips Ingenia 3.T and a Siemens Magnetom 1.5 T. One orthopaedic

practice was used to collate the data where there are two orthopaedic

surgeons.</td>
<td></td>
<td>Reasons for incomplete outcome data described? NR

Specificity could not be calculated as people with negative results did

not present for surgery.</td>
<td><table>
<tr><td></td><td>PTT +</td><td>PT T -</td><td></td></tr>
<tr><td>U S +</td><td>TP: 15</td><td>FP:</td><td></td></tr>
<tr><td>U S -</td><td>FN:</td><td>TN :</td><td></td></tr>
<tr><td></td><td>181 5</td><td></td><td>10 3</td></tr>
</table>
<table>
<tr><td></td><td>PT T +</td><td>PT T -</td><td></td></tr>
<tr><td>MR I +</td><td>TP: 14</td><td>FP:</td><td></td></tr>
<tr><td>MR I -</td><td>FN :</td><td>TN:</td><td></td></tr>
<tr><td></td><td>18</td><td></td><td>10 3</td></tr>
</table>
<u>Sensitivity</u>:

US: 83.3%

MRI: 77.7%

Partial thickness</td>
<td>indicates substantial agreement for supraspinatus tear diagnosis when

comparing MRI to USS performed in general imaging departments.

Given USS is less expensive and more available, it could be considered

as a first line screening tool when the main question is one of tendon

integrity. However, where the patient has significant underlying osteoarthritis,

MRI should be included in the imaging workup prior to

surgery.

As technology continues to improve with ultra-high frequency</td>
</tr>
</table>

[5] Comparator test is vergelijkbaar met de C uit de PICO van een interventievraag. Er kunnen ook meerdere tests worden vergeleken. Voeg die toe als comparator test 2 etc. Let op: de comparator test kan <u>nooit</u> de referentiestandaard zijn.

<table>
<tr><th>Study reference</th><th>Study characteristics</th><th>Patient characteristics</th><th>Index test
(test of interest)</th><th>Reference test</th><th>Follow-up</th><th>Outcome measures and effect size</th><th>Comments</th></tr>
<tr>
<td></td>
<td>Setting and country: single-centre, Australia

Funding and conflicts of interest: Funding: NR; Conflict of interest: Ann Quinton is an editorial board member for Sonography and a coauthor

on this article. Other declare no conflict of interest.</td>
<td>diagnostic accuracy of

pre-operative USS and MRI with arthroscopy used as gold standard.

Overall, there were 63 FTT and 40 PTT diagnosed by arthroscopy.

Prevalence PTT: 40 (38.8%)

Mean age (SD): 64 (10.8) years.

Sex: 63 males, 40 females</td>
<td>Cut-off point(s): Data were analysed reporting on number of supraspinatus tears reported as sensitivity (true positive case) for USS

and MRI and categorised into

1. number and sensitivity of partial thickness tears <than 5 mm

2. number and sensitivity of partial thickness tears >than 5 mm

3. number and sensitivity of full thickness tears</td>
<td></td>
<td></td>
<td>tear >5 mm
<table>
<tr><td></td><td>PTT
+</td><td>PTT
-</td><td></td></tr>
<tr><td>US
+</td><td>TP:
16</td><td>FP:</td><td></td></tr>
<tr><td>US
-</td><td>FN:</td><td>TN:</td><td></td></tr>
<tr><td></td><td>22</td><td></td><td>103</td></tr>
</table>

<table>
<tr><td></td><td>PT
T +</td><td>PT
T -</td><td></td></tr>
<tr><td>MR
I +</td><td>TP:
19</td><td>FP:</td><td></td></tr>
<tr><td>MR
I -</td><td>FN
:</td><td>TN:</td><td></td></tr>
<tr><td></td><td>22</td><td></td><td>10
3</td></tr>
</table>

<u>Sensitivity</u>:

US: 73.3%

MRI: 86.4%</td>
<td>ultrasound probes, further studies to assess accuracy of supraspinatus

tear detection should be evaluated, the assumption is that in future,

USS will have the capacity to serve as a stand-alone diagnostic tool in

accurately assessing supraspinatus tears prior to arthroscopy.</td>
</tr>
</table>

Study reference	Study characteristics	Patient characteristics	Index test (test of interest)	Reference test	Follow-up	Outcome measures and effect size	Comments
Zhu, 2022	Type of study[7]: prospective diagnostic accuracy study; to evaluate the diagnostic reliability of ultrasonography (US) and magnetic resonance imaging (MRI) for<br><br>subscapularis (SSC) tears with shoulder arthroscopy as the gold standard and to investigate the diagnostic value of 2 MRI	Inclusion criteria:<br><br>(1) patients who were diagnosed with RCTs by physical<br><br>examination, US, and MRI; and (2) preoperative MRI scans and US were performed within 3 months of the<br><br>arthroscopic shoulder procedures.	Index test: real-time US scanner (HI Vision<br><br>Ascendus, Hitachi Medical, Japan) and linear-array<br><br>transducer with a frequency of 12 to 18 MHz by a<br><br>single experienced sonographer who specialized in musculoskeletal US.<br><br>Cut-off point(s): NR<br><br>Comparator test[8]: MRI was performed using 1.5-T MRI equipment (Magnetom	Reference test[9]: Diagnostic Shoulder Arthroscopy<br><br>Cut-off point(s): NR	Time between the index test en reference test: < 3 months<br><br>For how many participants were no complete outcome data available?<br><br>N (%): NR<br><br>Reasons for incomplete outcome data described? NR	<u>Sensitivity:</u><br><br>US: 73.8% [65.5%-80.7%] (93/126)<br><br>MRI: 38.1% [29.7%-47.2%] (48/126)<br><br><u>Specificity:</u><br><br>US: 88.7% [84.8%-91.8%] (276/311)<br><br>MRI: 86.5% [82.3%-89.9%] (269/311)<br><br><u>Accuracy:</u><br><br>US: 84.4% [80.7%-87.5%] (369/437)	<u>Author conclusion:</u> The most important finding of the present study was<br><br>that the diagnostic accuracy of US was significantly<br><br>better than that of MRI for SSC tears. The data suggested that 1.5-T MRI was not quite reliable for diagnosing partial-thickness SSC tears, the sensitivity of<br><br>which was only 38.1%, whereas a standardized and<br><br>systematic US evaluation could provide much superior

[7] In geval van een case-control design moeten de patiëntkarakteristieken per groep (cases en controls) worden uitgewerkt. NB; case control studies zullen de accuratesse overschatten (Lijmer et al., 1999)

[8] Comparator test is vergelijkbaar met de C uit de PICO van een interventievraag. Er kunnen ook meerdere tests worden vergeleken. Voeg die toe als comparator test 2 etc. Let op: de comparator test kan <u>nooit</u> de referentiestandaard zijn.

[9] De referentiestandaard is de test waarmee definitief wordt aangetoond of iemand al dan niet ziek is. Idealiter is de referentiestandaard de Gouden standaard (100% sensitief en 100% specifiek). Let op! dit is niet de “comparison test/index 2”.

[4] Beschrijf de statistische parameters voor de vergelijking van de indextest(en) met de referentietest, en voor de vergelijking tussen de indextesten onderling (als er twee of meer indextesten worden vergeleken).

Study reference	Study characteristics	Patient characteristics	Index test (test of interest)	Reference test	Follow-up	Outcome measures and effect size	Comments
	signs (lesser tuberosity cysts and subcoracoid cysts) for SSC tears.<br><br>Setting and country: single-centre, China<br><br>Funding and conflicts of interest: The authors report the following potential conflicts of interest or sources of<br><br>funding: this work was supported by grant from Chongqing medical scientific<br><br>research project (No. 2021MSXM032).	Exclusion criteria: (1) patients with previous<br><br>shoulder surgery or shoulder fracture; (2) recurrent<br><br>shoulder instability; and (3) systemic inflammatory<br><br>disease.<br><br>N=437 (consecutive patient selection jan 2019- dec 2020)<br><br>Prevalence articular-side partial-thickness SSC	Essenza; Siemens Healthcare, Erlangen, Germany) with a dedicated shoulder coil.<br><br>Cut-off point(s): NR			MRI: 72.5% [68.2%-76.5%] (317/437)<br><br>PPV:<br><br>US: 72.7% [65.5%-80.7%] (93/128)<br><br>MRI: 53.3% [43.1%-63.3%] (48/90)<br><br>NPV:<br><br>US: 89.3% [85.4%-92.3%] (276/309)<br><br>MRI: 77.5% [72.8%-81.6%] (269/347)	reliability, with 73.8% sensitivity and 88.7%<br><br>specificity.

Study reference	Study characteristics	Patient characteristics	Index test (test of interest)	Reference test	Follow-up	Outcome measures and effect size	Comments
	Full ICMJE author disclosure forms<br><br>are available for this article online, as supplementary material.	tears: 126 (29%)<br><br>Mean age ± SD:<br><br>Sex: % M / % F<br><br>Other important characteristics:					

Module 4.1

What is the effectiveness of barbotage compared to shockwave in patients with tendinosis calcarean on patient-reported outcome measures?

tog bort: 51

Patients	patients with tendinosis calcarean of the supraspinatus or infraspinatus
Intervention	barbotage
Control	shockwave

tog bort: 51

Outcomes	Pain, PROMs for function (CMS, CASH, WORC, ASES, OSS, DSST), patient satisfaction, complications/adverse events, return to work or leisure

Embase.com

No.	Query	Results
#1	('shoulder impingement syndrome'/exp OR 'shoulder impingement syndrome*':ti,ab,kw OR 'subacromial impingement syndrome*':ti,ab,kw OR (('bursitis'/exp OR bursitis:ti,ab,kw OR 'tendinitis'/exp OR tendinitis:ti,ab,kw OR tendinosis:ti,ab,kw OR tendinopath*:ti,ab,kw OR 'impingement':ti,ab,kw) AND ('shoulder'/exp OR 'shoulder pain'/exp OR 'rotator cuff':ti,ab,kw OR acromion:ti,ab,kw OR acromial:ti,ab,kw OR subacromial:ti,ab,kw OR shoulder*:ti,ab,kw)) OR 'rotator cuff injury'/exp OR 'rotator cuff rupture'/exp OR ((('rotator cuff' OR 'shoulder cuff' OR infraspinatus OR supraspinatus OR subscapularis) NEAR/3 (tear* OR lesion* OR rupture* OR tendinopath* OR laceration*)):ti,ab,kw) OR (('full-thickness':ti,ab,kw OR partial:ti,ab,kw) AND 'rotator cuff':ti,ab,kw) OR ('rotator cuff'/exp AND ('rupture'/exp OR 'injury' OR 'laceration'/exp))) AND ('calcification'/de OR 'calcinosis'/exp OR calcifying:ti,ab,kw OR calcification:ti,ab,kw OR calcarea*:ti,ab,kw OR calcific:ti,ab,kw OR calcinosis:ti,ab,kw OR calcified:ti,ab,kw OR calcinotic:ti,ab,kw)	1352
#2	'barbotage'/exp OR 'lavage'/de OR 'guided needle'/exp OR barbotage*:ti,ab,kw OR (((needle* OR needling OR 'ultrasound guided' OR 'ultrasonography guided' OR 'us guided' OR 'image guided') NEAR/3 (aspiration OR lavage OR fragmentation OR irrigation OR punctur* OR treatment* OR therap* OR procedure*)):ti,ab,kw) OR ((('ultrasound guided' OR 'ultrasonography guided' OR 'us guided') NEAR/3 needl*):ti,ab,kw) OR ((percutaneous NEAR/3 (therap* OR treatment* OR intervention*)):ti,ab,kw)	196517

Formaterat: Avstånd Efter: 0 pt, Radavstånd: enkelt

Formaterat: Avstånd Efter: 0 pt, Radavstånd: enkelt

Formaterat: Avstånd Efter: 0 pt, Radavstånd: enkelt

#3	'shock wave therapy'/exp OR shockwave*:ti,ab,kw OR 'shock wave*':ti,ab,kw OR 'pulsed ultrasound*':ti,ab,kw OR 'ultrasonic vibration*':ti,ab,kw OR 'ultrasonic wave*':ti,ab,kw OR 'radiation ultrasound':ti,ab,kw OR 'ultrasound wave*':ti,ab,kw OR eswt:ti,ab,kw OR 'high energy':ti,ab,kw OR 'high intensity':ti,ab,kw OR 'low energy':ti,ab,kw OR 'low intensity':ti,ab,kw	160869
#4	#1 AND (#2 OR #3)	396
#5	#4 NOT ('conference abstract'/it OR 'editorial'/it OR 'letter'/it OR 'note'/it) NOT (('animal'/exp OR 'animal experiment'/exp OR 'animal model'/exp OR 'nonhuman'/exp) NOT 'human'/exp)	298
#6	#5 AND [01-11-2017]/sd	96
#7	'meta analysis'/exp OR 'meta analysis (topic)'/exp OR metaanaly*:ti,ab OR 'meta analy*':ti,ab OR metanaly*:ti,ab OR 'systematic review'/de OR 'cochrane database of systematic reviews'/jt OR prisma:ti,ab OR prospero:ti,ab OR (((systemati* OR scoping OR umbrella OR 'structured literature') NEAR/3 (review* OR overview*)):ti,ab) OR ((systemic* NEAR/1 review*):ti,ab) OR (((systemati* OR literature OR database* OR 'data base*') NEAR/10 search*):ti,ab) OR (((structured OR comprehensive* OR systemic*) NEAR/3 search*):ti,ab) OR (((literature NEAR/3 review*):ti,ab) AND (search*:ti,ab OR database*:ti,ab OR 'data base*':ti,ab)) OR (('data extraction':ti,ab OR 'data source*':ti,ab) AND 'study selection':ti,ab) OR ('search strategy':ti,ab AND 'selection criteria':ti,ab) OR ('data source*':ti,ab AND 'data synthesis':ti,ab) OR medline:ab OR pubmed:ab OR embase:ab OR cochrane:ab OR (((critical OR rapid) NEAR/2 (review* OR overview* OR synthes*)):ti) OR ((((critical* OR rapid*) NEAR/3 (review* OR overview* OR synthes*)):ab) AND (search*:ab OR database*:ab OR 'data base*':ab)) OR metasynthes*:ti,ab OR 'meta synthes*':ti,ab	970896
#8	'clinical trial'/exp OR 'randomization'/exp OR 'single blind procedure'/exp OR 'double blind procedure'/exp OR 'crossover procedure'/exp OR 'placebo'/exp OR 'prospective study'/exp OR rct:ab,ti OR random*:ab,ti OR 'single blind':ab,ti OR 'randomised controlled trial':ab,ti OR 'randomized controlled trial'/exp OR placebo*:ab,ti	3896362
#9	#6 AND #7 = SR	15
#10	#6 AND #8 NOT #9 = RCT	34
#11	#9 OR #10	49

Formaterat: Avstånd Efter: 0 pt, Radavstånd: enkelt
Formaterat: Avstånd Efter: 0 pt, Radavstånd: enkelt
Formaterat: Avstånd Efter: 0 pt, Radavstånd: enkelt
Formaterat: Avstånd Efter: 0 pt, Radavstånd: enkelt
Formaterat: Avstånd Efter: 0 pt, Radavstånd: enkelt
Formaterat: Avstånd Efter: 0 pt, Radavstånd: enkelt
Formaterat: Avstånd Efter: 0 pt, Radavstånd: enkelt
Formaterat: Avstånd Efter: 0 pt, Radavstånd: enkelt
Formaterat: Avstånd Efter: 0 pt, Radavstånd: enkelt

Ovid/Medline

#	Searches	Results
1	(Shoulder Impingement Syndrome/ or 'shoulder impingement syndrome*'.ti,ab,kf. or 'subacromial impingement syndrome*'.ti,ab,kf. or ((exp bursitis/ or exp Tendinopathy/ or bursitis.ti,ab,kf. or tendinitis.ti,ab,kf. or tendinosis.ti,ab,kf. or tendinopath*.ti,ab,kf. or 'impingement'.ti,ab,kf.) and (exp Shoulder/ or 'rotator cuff'.ti,ab,kf. or acromion.ti,ab,kf. or acromial.ti,ab,kf. or subacromial.ti,ab,kf. or shoulder*.ti,ab,kf.)) or Rotator Cuff/ or exp Rotator Cuff Injuries/ or (('rotator cuff' or 'shoulder cuff' or infraspinatus or supraspinatus or subscapularis) adj3 (tear* or lesion* or rupture* or tendinopath* or laceration*)).ti,ab,kf. or (('full-thickness' or partial) and 'rotator cuff').ti,ab,kf.) and (exp Calcinosis/ or calcifying.ti,ab,kf. or calcification.ti,ab,kf. or calcarea*.ti,ab,kf. or calcific.ti,ab,kf. or calcinosis.ti,ab,kf. or calcified.ti,ab,kf. or calcinotic.ti,ab,kf.)	1025
2	exp Ultrasonography, Interventional/ or Therapeutic Irrigation/ or barbotage*.ti,ab,kf. or ((needle* or needling or 'ultrasound guided' or 'ultrasonography guided' or 'us guided' or 'image guided') adj3 (aspiration or lavage or fragmentation or irrigation or punctur* or treatment* or therap* or procedure*)).ti,ab,kf. or (('ultrasound guided' or 'ultrasonography guided' or 'us guided') adj3 needl*).ti,ab,kf. or (percutaneous adj3 (therap* or treatment* or intervention*)).ti,ab,kf.	156933
3	exp Extracorporeal Shockwave Therapy/ or exp Ultrasonic Waves/ or (shockwave* or 'shock wave*' or 'pulsed ultrasound*' or 'ultrasonic vibration*' or 'ultrasonic wave*' or 'radiation ultrasound' or 'ultrasound wave*' or eswt or 'high energy' or 'high intensity' or 'low energy' or 'low intensity').ti,ab,kf.	146200
4	1 and (2 or 3)	298
5	4 not (comment/ or editorial/ or letter/) not ((exp animals/ or exp models, animal/) not humans/)	280
6	5 and 20171101:20231023.(dt).	78
7	meta-analysis/ or meta-analysis as topic/ or (metaanaly* or meta-analy* or metanaly*).ti,ab,kf. or systematic review/ or cochrane.jw. or (prisma or prospero).ti,ab,kf. or ((systemati* or scoping or umbrella or "structured literature") adj3 (review* or overview*)).ti,ab,kf. or (systemic* adj1 review*).ti,ab,kf. or ((systemati* or literature or database* or data-base*) adj10 search*).ti,ab,kf. or ((structured or comprehensive* or systemic*) adj3 search*).ti,ab,kf. or ((literature adj3 review*) and (search* or database* or	700911

Formaterat: Avstånd Efter: 0 pt, Radavstånd: enkelt
Formaterat: Avstånd Efter: 0 pt, Radavstånd: enkelt
Formaterat: Avstånd Efter: 0 pt, Radavstånd: enkelt
Formaterat: Avstånd Efter: 0 pt, Radavstånd: enkelt
Formaterat: Avstånd Efter: 0 pt, Radavstånd: enkelt
Formaterat: Avstånd Efter: 0 pt, Radavstånd: enkelt
Formaterat: Avstånd Efter: 0 pt, Radavstånd: enkelt
Formaterat: Avstånd Efter: 0 pt, Radavstånd: enkelt
Formaterat: Avstånd Efter: 0 pt, Radavstånd: enkelt

	data-base*)).ti,ab,kf. or (("data extraction" or "data source*") and "study selection").ti,ab,kf. or ("search strategy" and "selection criteria").ti,ab,kf. or ("data source*" and "data synthesis").ti,ab,kf. or (medline or pubmed or embase or cochrane).ab. or ((critical or rapid) adj2 (review* or overview* or synthes*)).ti. or (((critical* or rapid*) adj3 (review* or overview* or synthes*)) and (search* or database* or data-base*)).ab. or (metasynthes* or meta-synthes*).ti,ab,kf.	
8	exp clinical trial/ or randomized controlled trial/ or exp clinical trials as topic/ or randomized controlled trials as topic/ or Random Allocation/ or Double-Blind Method/ or Single-Blind Method/ or (clinical trial, phase i or clinical trial, phase ii or clinical trial, phase iii or clinical trial, phase iv or controlled clinical trial or randomized controlled trial or multicenter study or clinical trial).pt. or random*.ti,ab. or (clinic* adj trial*).tw. or ((singl* or doubl* or treb* or tripl*) adj (blind$3 or mask$3)).tw. or Placebos/ or placebo*.tw.	2645320
9	6 and 7 = SR	12
10	(6 and 8) not 9 = RCT	17
11	9 or 10	29

Formaterat: Avstånd Efter: 0 pt, Radavstånd: enkelt

Formaterat: Avstånd Efter: 0 pt, Radavstånd: enkelt

Formaterat: Avstånd Efter: 0 pt, Radavstånd: enkelt

Formaterat: Avstånd Efter: 0 pt, Radavstånd: enkelt

Module 4.2

What are the benefits and harms of subacromial corticosteroid injection in addition to exercise in patients with subacromial shoulder complaints?

Patients	patients with SAPS
Intervention	exercise therapy
Control	corticosteroid injection + exercise therapy
Outcomes	pain, function, complications, recurrence, patient satisfaction, return to work

Embase.com

No.	Query	Results
#1	'shoulder impingement syndrome'/exp OR 'shoulder impingement syndrome':ti,ab,kw OR 'subacromial impingement syndrome':ti,ab,kw OR (((subacromial OR shoulder) NEAR/3 pain*):ti,ab,kw) OR (('bursitis'/exp OR bursitis:ti,ab,kw OR 'tendinitis'/exp OR tendinitis:ti,ab,kw OR 'impingement':ti,ab,kw) AND ('shoulder'/exp OR 'rotator cuff':ti,ab,kw OR acromion:ti,ab,kw OR acromial:ti,ab,kw OR subacromial:ti,ab,kw OR shoulder*:ti,ab,kw)) OR 'rotator cuff injury'/exp OR 'rotator cuff rupture'/exp OR ((('rotator cuff' OR 'shoulder cuff' OR infraspinatus OR supraspinatus OR subscapularis) NEAR/3 (tear* OR lesion* OR rupture* OR tendinopath* OR laceration*)):ti,ab,kw) OR (('full-thickness':ti,ab,kw OR partial:ti,ab,kw) AND 'rotator cuff':ti,ab,kw) OR ('rotator cuff'/exp AND ('rupture'/exp OR 'injury'/de OR 'laceration'/exp))	37131
#2	'corticosteroid'/exp OR 'corticosteroid therapy'/exp OR corticosteroid*:ti,ab,kw OR glucocortico*:ti,ab,kw OR 'adrenal cortex hormone*':ti,ab,kw OR 'adrenal steroid*':ti,ab,kw OR corticoid*:ti,ab,kw OR corticotherap*:ti,ab,kw OR 'steroid'/exp OR 'steroid therapy'/exp OR 'cyclosteroid*':ti,ab,kw OR 'steroid*':ti,ab,kw OR 'triamcinolone'/exp OR 'triamcinolone derivative'/exp OR 'triamcinolon*':ti,ab,kw	2156208
#3	'physiotherapy'/exp OR 'kinesiotherapy'/exp OR 'occupational therapy'/exp OR kinesiotherap*:ti,ab,kw OR 'occupation* therap*':ti,ab,kw OR ergotherap*:ti,ab,kw OR exercis*:ti,ab,kw OR physiotherap*:ti,ab,kw OR 'physio therap*':ti,ab,kw OR 'physical therap*':ti,ab,kw OR (((functional OR muscular) NEAR/3 training):ti,ab,kw) OR 'manual therap*':ti,ab,kw OR 'manipulat* therap*':ti,ab,kw OR ((musculoskeletal NEAR/3 manipulation*):ti,ab,kw)	728720
#4	#1 AND #2 AND #3 NOT ('conference abstract'/it OR 'editorial'/it OR 'letter'/it OR 'note'/it) NOT (('animal'/exp OR 'animal experiment'/exp OR 'animal model'/exp OR 'nonhuman'/exp) NOT 'human'/exp) AND [01-01-2017]/sd	468
#5	'meta analysis'/exp OR 'meta analysis (topic)'/exp OR metaanaly*:ti,ab OR 'meta analy*':ti,ab OR metanaly*:ti,ab OR 'systematic review'/de OR 'cochrane	1008329

Formaterat: Avstånd Efter: 0 pt, Radavstånd: enkelt

Formaterad tabell

Formaterat: Avstånd Efter: 0 pt, Radavstånd: enkelt

Formaterat: Avstånd Efter: 0 pt, Radavstånd: enkelt

Formaterat: Avstånd Efter: 0 pt, Radavstånd: enkelt

Formaterat: Avstånd Efter: 0 pt, Radavstånd: enkelt

Formaterat: Avstånd Efter: 0 pt, Radavstånd: enkelt

	database of systematic reviews'/jt OR prisma:ti,ab OR prospero:ti,ab OR (((systemati* OR scoping OR umbrella OR 'structured literature') NEAR/3 (review* OR overview*)):ti,ab) OR ((systemic* NEAR/1 review*):ti,ab) OR (((systemati* OR literature OR database* OR 'data base*') NEAR/10 search*):ti,ab) OR (((structured OR comprehensive* OR systemic*) NEAR/3 search*):ti,ab) OR (((literature NEAR/3 review*):ti,ab) AND (search*:ti,ab OR database*:ti,ab OR 'data base*':ti,ab)) OR (('data extraction':ti,ab OR 'data source*':ti,ab) AND 'study selection':ti,ab) OR ('search strategy':ti,ab AND 'selection criteria':ti,ab) OR ('data source*':ti,ab AND 'data synthesis':ti,ab) OR medline:ab OR pubmed:ab OR embase:ab OR cochrane:ab OR (((critical OR rapid) NEAR/2 (review* OR overview* OR synthes*)):ti) OR ((((critical* OR rapid*) NEAR/3 (review* OR overview* OR synthes*)):ab) AND (search*:ab OR database*:ab OR 'data base*':ab)) OR metasynthes*:ti,ab OR 'meta synthes*':ti,ab	
#6	'clinical trial'/exp OR 'randomization'/exp OR 'single blind procedure'/exp OR 'double blind procedure'/exp OR 'crossover procedure'/exp OR 'placebo'/exp OR 'prospective study'/exp OR rct:ab,ti OR random*:ab,ti OR 'single blind':ab,ti OR 'randomised controlled trial':ab,ti OR 'randomized controlled trial'/exp OR placebo*:ab,ti	3987109
#7	#4 AND #5 - SR	86
#8	#4 AND #6 NOT #7 - RCT	133
#9	#7 OR #8	219

Ovid/Medline

#	Searches	Results
1	Shoulder Impingement Syndrome/ or 'shoulder impingement syndrome'.ti,ab,kf. or 'subacromial impingement syndrome'.ti,ab,kf. or ((subacromial or shoulder) adj3 pain*).ti,ab,kf. or ((exp bursitis/ or exp Tendinopathy/ or bursitis.ti,ab,kf. or tendinitis.ti,ab,kf. or 'impingement'.ti,ab,kf.) and (exp Shoulder/ or 'rotator cuff'.ti,ab,kf. or acromion.ti,ab,kf. or acromial.ti,ab,kf. or subacromial.ti,ab,kf. or shoulder*.ti,ab,kf.)) or Rotator Cuff/ or exp Rotator Cuff Injuries/ or (('rotator cuff' or	29934

Formaterat: Avstånd Efter: 0 pt, Radavstånd: enkelt

Formaterat: Avstånd Efter: 0 pt, Radavstånd: enkelt

Formaterat: Avstånd Efter: 0 pt, Radavstånd: enkelt

Formaterat: Avstånd Efter: 0 pt, Radavstånd: enkelt

Formaterat: Radavstånd: enkelt

Formaterat: Radavstånd: enkelt

	'shoulder cuff' or infraspinatus or supraspinatus or subscapularis) adj3 (tear* or lesion* or rupture* or tendinopath* or laceration*)).ti,ab,kf. or (('full-thickness' or partial) and 'rotator cuff').ti,ab,kf.	
2	exp Adrenal Cortex Hormones/ or corticosteroid*.ti,ab,kf. or glucocortico*.ti,ab,kf. or 'adrenal cortex hormone*'.ti,ab,kf. or 'adrenal steroid*'.ti,ab,kf. or corticoid*.ti,ab,kf. or corticotherap*.ti,ab,kf. or exp Steroids/ or 'cyclosteroid*'.ti,ab,kf. or 'steroid*'.ti,ab,kf. or exp Triamcinolone/ or 'triamcinolon*'.ti,ab,kf.	1247865
3	exp Physical Therapy Modalities/ or exp Occupational Therapy/ or kinesiotherap*.ti,ab,kf. or 'occupation* therap*'.ti,ab,kf. or ergotherap*.ti,ab,kf. or exercis*.ti,ab,kf. or physiotherap*.ti,ab,kf. or 'physio therap*'.ti,ab,kf. or 'physical therap*'.ti,ab,kf. or ((functional or muscular) adj3 training).ti,ab,kf. or 'manual therap*'.ti,ab,kf. or 'manipulat* therap*'.ti,ab,kf. or (musculoskeletal adj3 manipulation*).ti,ab,kf.	564636
4	(1 and 2 and 3) not (comment/ or editorial/ or letter/) not ((exp animals/ or exp models, animal/) not humans/)	639
5	limit 4 to dt="20170101-20240308"	258
6	meta-analysis/ or meta-analysis as topic/ or (metaanaly* or meta-analy* or metanaly*).ti,ab,kf. or systematic review/ or cochrane.jw. or (prisma or prospero).ti,ab,kf. or ((systemati* or scoping or umbrella or "structured literature") adj3 (review* or overview*)).ti,ab,kf. or (systemic* adj1 review*).ti,ab,kf. or ((systemati* or literature or database* or data-base*) adj10 search*).ti,ab,kf. or ((structured or comprehensive* or systemic*) adj3 search*).ti,ab,kf. or ((literature adj3 review*) and (search* or database* or data-base*)).ti,ab,kf. or (("data extraction" or "data source*") and "study selection").ti,ab,kf. or ("search strategy" and "selection criteria").ti,ab,kf. or ("data source*" and "data synthesis").ti,ab,kf. or (medline or pubmed or embase or cochrane).ab. or ((critical or rapid) adj2 (review* or overview* or synthes*)).ti. or (((critical* or rapid*) adj3 (review* or overview* or	730984

Formaterat: Radavstånd: enkelt

Formaterat: Radavstånd: enkelt

Formaterat: Radavstånd: enkelt

Formaterat: Radavstånd: enkelt

Formaterat: Radavstånd: enkelt

	synthes*)) and (search* or database* or data-base*)).ab. or (metasynthes* or meta-synthes*).ti,ab,kf.	
7	exp clinical trial/ or randomized controlled trial/ or exp clinical trials as topic/ or randomized controlled trials as topic/ or Random Allocation/ or Double-Blind Method/ or Single-Blind Method/ or (clinical trial, phase i or clinical trial, phase ii or clinical trial, phase iii or clinical trial, phase iv or controlled clinical trial or randomized controlled trial or multicenter study or clinical trial).pt. or random*.ti,ab. or (clinic* adj trial*).tw. or ((singl* or doubl* or treb* or tripl*) adj (blind$3 or mask$3)).tw. or Placebos/ or placebo*.tw.	2698926
8	5 and 6 - SR	55
9	(5 and 7) not 8 - RCT	86
10	8 or 9	141

Formaterat: Radavstånd: enkelt

Formaterat: Radavstånd: enkelt

Formaterat: Radavstånd: enkelt

Formaterat: Radavstånd: enkelt

Module 4.3

What is the effectiveness of suprascapularis blokkade vs. corticosteroid injection in SAPS patients on patient-reported outcome measures?

Patients	patients with SAPS
Intervention	suprascapular nerve block (SSNB)
Control	subacromial infiltration with corticosteroid
Outcomes	pain reduction, function (constant Murley score), quality of life, rehabilitation time, return to work or leisure

Embase.com

No.	Query	Results
#1	'shoulder impingement syndrome'/exp OR 'shoulder impingement syndrome':ti,ab,kw OR 'subacromial impingement syndrome':ti,ab,kw OR (((subacromial OR shoulder) NEAR/3 pain*):ti,ab,kw) OR (('bursitis'/exp OR bursitis:ti,ab,kw OR 'tendinitis'/exp OR tendinitis:ti,ab,kw OR 'impingement':ti,ab,kw) AND ('shoulder'/exp OR 'rotator cuff':ti,ab,kw OR acromion:ti,ab,kw OR acromial:ti,ab,kw OR subacromial:ti,ab,kw OR shoulder*:ti,ab,kw)) OR 'rotator cuff injury'/exp OR 'rotator cuff rupture'/exp OR ((('rotator cuff' OR 'shoulder cuff' OR infraspinatus OR supraspinatus OR subscapularis) NEAR/3 (tear* OR lesion* OR rupture* OR tendinopath* OR laceration*)):ti,ab,kw) OR (('full-thickness':ti,ab,kw OR partial:ti,ab,kw) AND 'rotator cuff':ti,ab,kw) OR ('rotator cuff'/exp AND ('rupture'/exp OR 'injury'/de OR 'laceration'/exp))	36149
#2	'suprascapular nerve block'/exp OR 'nerve block'/exp OR (((nerve* OR neuro* OR suprascapular OR supraclavicular OR brachial) NEAR/3 block*):ti,ab,kw) OR ssnb:ti,ab,kw	84041
#3	#1 AND #2	966
#4	#3 NOT ('conference abstract'/it OR 'editorial'/it OR 'letter'/it OR 'note'/it) NOT (('animal'/exp OR 'animal experiment'/exp OR 'animal model'/exp OR 'nonhuman'/exp) NOT 'human'/exp)	678
#5	#4 AND [2013-2023]/py	445

Formaterat: Avstånd Efter: 0 pt, Radavstånd: enkelt
Formaterat: Avstånd Efter: 0 pt, Radavstånd: enkelt
Formaterat: Avstånd Efter: 0 pt, Radavstånd: enkelt
Formaterat: Avstånd Efter: 0 pt, Radavstånd: enkelt
Formaterat: Avstånd Efter: 0 pt, Radavstånd: enkelt
Formaterat: Avstånd Efter: 0 pt, Radavstånd: enkelt

#6	'meta analysis'/exp OR 'meta analysis (topic)'/exp OR metaanaly*:ti,ab OR 'meta analy*':ti,ab OR metanaly*:ti,ab OR 'systematic review'/de OR 'cochrane database of systematic reviews'/jt OR prisma:ti,ab OR prospero:ti,ab OR (((systemati* OR scoping OR umbrella OR 'structured literature') NEAR/3 (review* OR overview*)):ti,ab) OR ((systemic* NEAR/1 review*):ti,ab) OR (((systemati* OR literature OR database* OR 'data base*') NEAR/10 search*):ti,ab) OR (((structured OR comprehensive* OR systemic*) NEAR/3 search*):ti,ab) OR (((literature NEAR/3 review*):ti,ab) AND (search*:ti,ab OR database*:ti,ab OR 'data base*':ti,ab)) OR (('data extraction':ti,ab OR 'data source*':ti,ab) AND 'study selection':ti,ab) OR ('search strategy':ti,ab AND 'selection criteria':ti,ab) OR ('data source*':ti,ab AND 'data synthesis':ti,ab) OR medline:ab OR pubmed:ab OR embase:ab OR cochrane:ab OR (((critical OR rapid) NEAR/2 (review* OR overview* OR synthes*)):ti) OR ((((critical* OR rapid*) NEAR/3 (review* OR overview* OR synthes*)):ab) AND (search*:ab OR database*:ab OR 'data base*':ab)) OR metasynthes*:ti,ab OR 'meta synthes*':ti,ab	969507
#7	'clinical trial'/exp OR 'randomization'/exp OR 'single blind procedure'/exp OR 'double blind procedure'/exp OR 'crossover procedure'/exp OR 'placebo'/exp OR 'prospective study'/exp OR rct:ab,ti OR random*:ab,ti OR 'single blind':ab,ti OR 'randomised controlled trial':ab,ti OR 'randomized controlled trial'/exp OR placebo*:ab,ti	3892820
#8	'major clinical study'/de OR 'clinical study'/de OR 'case control study'/de OR 'family study'/de OR 'longitudinal study'/de OR 'retrospective study'/de OR 'prospective study'/de OR 'comparative study'/de OR 'cohort analysis'/de OR ((cohort NEAR/1 (study OR studies)):ab,ti) OR (('case control' NEAR/1 (study OR studies)):ab,ti) OR (('follow up' NEAR/1 (study OR studies)):ab,ti) OR (observational NEAR/1 (study OR studies)) OR ((epidemiologic NEAR/1 (study OR studies)):ab,ti) OR (('cross sectional' NEAR/1 (study OR studies)):ab,ti)	7881241
#9	'case control study'/de OR 'comparative study'/exp OR 'control group'/de OR 'controlled study'/de OR 'controlled clinical trial'/de OR 'crossover procedure'/de OR 'double blind procedure'/de OR 'phase 2 clinical trial'/de OR 'phase 3 clinical trial'/de OR 'phase 4 clinical trial'/de OR 'pretest posttest design'/de OR 'pretest posttest control group design'/de OR 'quasi experimental study'/de OR 'single blind procedure'/de OR 'triple blind procedure'/de OR (((control OR controlled) NEAR/6 trial):ti,ab,kw) OR (((control OR controlled) NEAR/6 (study OR studies)):ti,ab,kw) OR (((control OR controlled) NEAR/1 active):ti,ab,kw) OR 'open label*':ti,ab,kw OR (((double OR two OR three OR multi OR trial) NEAR/1 (arm OR arms)):ti,ab,kw) OR ((allocat* NEAR/10 (arm OR arms)):ti,ab,kw) OR placebo*:ti,ab,kw OR	14494134

Formaterat: Avstånd Efter: 0 pt, Radavstånd: enkelt

Formaterat: Avstånd Efter: 0 pt, Radavstånd: enkelt

Formaterat: Avstånd Efter: 0 pt, Radavstånd: enkelt

Formaterat: Avstånd Efter: 0 pt, Radavstånd: enkelt

	'sham-control*':ti,ab,kw OR (((single OR double OR triple OR assessor) NEAR/1 (blind* OR masked)):ti,ab,kw) OR nonrandom*:ti,ab,kw OR 'non-random*':ti,ab,kw OR 'quasi-experiment*':ti,ab,kw OR crossover:ti,ab,kw OR 'cross over':ti,ab,kw OR 'parallel group*':ti,ab,kw OR 'factorial trial':ti,ab,kw OR ((phase NEAR/5 (study OR trial)):ti,ab,kw) OR ((case* NEAR/6 (matched OR control*)):ti,ab,kw) OR ((match* NEAR/6 (pair OR pairs OR cohort* OR control* OR group* OR healthy OR age OR sex OR gender OR patient* OR subject* OR participant*)):ti,ab,kw) OR ((propensity NEAR/6 (scor* OR match*)):ti,ab,kw) OR versus:ti OR vs:ti OR compar*:ti OR ((compar* NEAR/1 study):ti,ab,kw) OR (('major clinical study'/de OR 'clinical study'/de OR 'cohort analysis'/de OR 'observational study'/de OR 'cross-sectional study'/de OR 'multicenter study'/de OR 'correlational study'/de OR 'follow up'/de OR cohort*:ti,ab,kw OR 'follow up':ti,ab,kw OR followup:ti,ab,kw OR longitudinal*:ti,ab,kw OR prospective*:ti,ab,kw OR retrospective*:ti,ab,kw OR observational*:ti,ab,kw OR 'cross sectional*':ti,ab,kw OR cross?ectional*:ti,ab,kw OR multicent*:ti,ab,kw OR 'multi-cent*':ti,ab,kw OR consecutive*:ti,ab,kw) AND (group:ti,ab,kw OR groups:ti,ab,kw OR subgroup*:ti,ab,kw OR versus:ti,ab,kw OR vs:ti,ab,kw OR compar*:ti,ab,kw OR 'odds ratio*':ab OR 'relative odds':ab OR 'risk ratio*':ab OR 'relative risk*':ab OR 'rate ratio':ab OR aor:ab OR arr:ab OR rrr:ab OR ((('or' OR 'rr') NEAR/6 ci):ab)))	
#10	#5 AND #6 **– SR's**	55
#11	#5 AND #7 NOT #10 **– RCT's**	186
#12	#5 AND (#8 OR #9) NOT (#10 OR #11) **- Observationeel**	88
#13	#10 OR #11 OR #12	329

Formaterat: Avstånd Efter: 0 pt, Radavstånd: enkelt
Formaterat: Avstånd Efter: 0 pt, Radavstånd: enkelt
Formaterat: Avstånd Efter: 0 pt, Radavstånd: enkelt
Formaterat: Avstånd Efter: 0 pt, Radavstånd: enkelt

Ovid/Medline 16-10-2023

#	Searches	Results
1	Shoulder Impingement Syndrome/ or 'shoulder impingement syndrome'.ti,ab,kf. or 'subacromial impingement syndrome'.ti,ab,kf. or ((subacromial or shoulder) adj3 pain*).ti,ab,kf. or ((exp bursitis/ or exp Tendinopathy/ or bursitis.ti,ab,kf. or tendinitis.ti,ab,kf. or 'impingement'.ti,ab,kf.) and (exp Shoulder/ or 'rotator cuff'.ti,ab,kf. or acromion.ti,ab,kf. or acromial.ti,ab,kf. or subacromial.ti,ab,kf. or shoulder*.ti,ab,kf.)) or Rotator Cuff/ or exp Rotator Cuff Injuries/ or (('rotator cuff' or 'shoulder cuff' or infraspinatus or	29052

Formaterat: Avstånd Före: 0 pt, Efter: 0 pt, Radavstånd: enkelt
Formaterat: Avstånd Efter: 0 pt, Radavstånd: enkelt

	supraspinatus or subscapularis) adj3 (tear* or lesion* or rupture* or tendinopath* or laceration*)).ti,ab,kf. or (('full-thickness' or partial) and 'rotator cuff').ti,ab,kf.	
2	exp Nerve Block/ or ((nerve* or neuro* or suprascapular or supraclavicular or brachial) adj3 block*).ti,ab,kf. or ssnb.ti,ab,kf.	55198
3	1 and 2	591
4	3 not (comment/ or editorial/ or letter/) not ((exp animals/ or exp models, animal/) not humans/)	552
5	limit 4 to yr=2013-2023	356
6	meta-analysis/ or meta-analysis as topic/ or (metaanaly* or meta-analy* or metanaly*).ti,ab,kf. or systematic review/ or cochrane.jw. or (prisma or prospero).ti,ab,kf. or ((systemati* or scoping or umbrella or "structured literature") adj3 (review* or overview*)).ti,ab,kf. or (systemic* adj1 review*).ti,ab,kf. or ((systemati* or literature or database* or data-base*) adj10 search*).ti,ab,kf. or ((structured or comprehensive* or systemic*) adj3 search*).ti,ab,kf. or ((literature adj3 review*) and (search* or database* or data-base*)).ti,ab,kf. or (("data extraction" or "data source*") and "study selection").ti,ab,kf. or ("search strategy" and "selection criteria").ti,ab,kf. or ("data source*" and "data synthesis").ti,ab,kf. or (medline or pubmed or embase or cochrane).ab. or ((critical or rapid) adj2 (review* or overview* or synthes*)).ti. or (((critical* or rapid*) adj3 (review* or overview* or synthes*)) and (search* or database* or data-base*)).ab. or (metasynthes* or meta-synthes*).ti,ab,kf.	699693
7	exp clinical trial/ or randomized controlled trial/ or exp clinical trials as topic/ or randomized controlled trials as topic/ or Random Allocation/ or Double-Blind Method/ or Single-Blind Method/ or (clinical trial, phase i or clinical trial, phase ii or clinical trial, phase iii or clinical trial, phase iv or controlled clinical trial or randomized controlled trial or multicenter study or clinical trial).pt. or random*.ti,ab. or (clinic* adj trial*).tw. or ((singl* or doubl* or treb* or tripl*) adj (blind$3 or mask$3)).tw. or Placebos/ or placebo*.tw.	2643324
8	Epidemiologic studies/ or case control studies/ or exp cohort studies/ or Controlled Before-After Studies/ or Case control.tw. or cohort.tw. or Cohort analy$.tw. or (Follow up adj (study or studies)).tw. or (observational adj (study or studies)).tw. or Longitudinal.tw. or Retrospective*.tw. or prospective*.tw. or consecutive*.tw. or Cross sectional.tw. or Cross-sectional studies/ or historically controlled study/ or interrupted time series analysis/ [Onder exp cohort studies vallen ook longitudinale, prospectieve en retrospectieve studies]	4553864
9	Case-control Studies/ or clinical trial, phase ii/ or clinical trial, phase iii/ or clinical trial, phase iv/ or comparative study/ or control groups/ or controlled before-after studies/ or controlled clinical trial/ or double-blind method/ or historically controlled study/ or matched-pair analysis/ or single-blind method/ or (((control or controlled) adj6 (study or studies or trial)) or (compar* adj (study or studies)) or ((control or controlled) adj1 active) or "open label*" or ((double or two or three or multi or trial) adj (arm or arms)) or (allocat* adj10 (arm or arms)) or placebo* or "sham-control*" or ((single or double or triple or assessor) adj1 (blind* or masked)) or nonrandom* or "non-random*" or "quasi-experiment*" or "parallel group*" or "factorial trial" or "pretest posttest" or (phase adj5 (study or trial)) or (case* adj6 (matched or control*)) or (match* adj6 (pair or pairs or cohort* or control* or group* or healthy or age or sex or gender or patient* or subject* or participant*)) or (propensity adj6 (scor* or match*))).ti,ab,kf. or (confounding adj6 adjust*).ti,ab. or (versus or vs or compar*).ti. or ((exp cohort studies/ or epidemiologic studies/ or multicenter study/ or	5531506

Formaterat: Avstånd Efter: 0 pt, Radavstånd: enkelt
Formaterat: Avstånd Efter: 0 pt, Radavstånd: enkelt
Formaterat: Avstånd Efter: 0 pt, Radavstånd: enkelt
Formaterat: Avstånd Efter: 0 pt, Radavstånd: enkelt
Formaterat: Avstånd Efter: 0 pt, Radavstånd: enkelt
Formaterat: Avstånd Efter: 0 pt, Radavstånd: enkelt
Formaterat: Avstånd Efter: 0 pt, Radavstånd: enkelt
Formaterat: Avstånd Efter: 0 pt, Radavstånd: enkelt

	observational study/ or seroepidemiologic studies/ or (cohort* or 'follow up' or followup or longitudinal* or prospective* or retrospective* or observational* or multicent* or 'multi-cent*' or consecutive*).ti,ab,kf.) and ((group or groups or subgroup* or versus or vs or compar*).ti,ab,kf. or ('odds ratio*' or 'relative odds' or 'risk ratio*' or 'relative risk*' or aor or arr or rrr).ab. or (("OR" or "RR") adj6 CI).ab.))	
10	5 and 6 **– SR's**	39
11	(5 and 7) not 10 **– RCT's**	145
12	(5 and (8 or 9)) not (10 or 11) **- Observationeel**	73
13	10 or 11 or 12	257

Formaterat: Avstånd Efter: 0 pt, Radavstånd: enkelt

Formaterat: Avstånd Efter: 0 pt, Radavstånd: enkelt

Formaterat: Avstånd Efter: 0 pt, Radavstånd: enkelt

Formaterat: Avstånd Efter: 0 pt, Radavstånd: enkelt

Module 5.1

What is the effectivity of cuff repair compared with physiotherapy with or without corticosteroid injection on patient-reported outcome measures in adult patients (<70 years) with an isolated symptomatic, nontraumatic, supraspinatus tear?

Patients	Adults (<70 years) with an isolated symptomatic, nontraumatic, supraspinatus tear
Intervention	rotator cuff repair (surgery)
Control	physiotherapy with or without injection of corticosteroids
Outcomes	Pain, complications/adverse events, patient reported outcomes measures for function (CMS, DASH, WORC, ASES, OSS, DSST), patient satisfaction, return to work or leisure

Embase.com

No.	Query	Results
#17	#15 OR #16	337
#16	#12 AND #14 NOT #15 **= RCT**	201
#15	#12 AND #13 **= SR**	136
#14	'randomized controlled trial'/exp OR random*:ti,ab OR (((pragmatic OR practical) NEAR/1 'clinical trial*'):ti,ab) OR ((('non inferiority' OR noninferiority OR superiority OR equivalence) NEAR/3 trial*):ti,ab) OR rct:ti,ab,kw	2015900
#13	'meta analysis'/exp OR 'meta analysis (topic)'/exp OR metaanaly*:ti,ab OR 'meta analy*':ti,ab OR metanaly*:ti,ab OR 'systematic review'/de OR 'cochrane database of systematic reviews'/jt OR prisma:ti,ab OR prospero:ti,ab OR (((systemati* OR scoping OR umbrella OR 'structured literature')	733409

	NEAR/3 (review* OR overview*)):ti,ab) OR ((systemic* NEAR/1 review*):ti,ab) OR (((systemati* OR literature OR database* OR 'data base*') NEAR/10 search*):ti,ab) OR (((structured OR comprehensive* OR systemic*) NEAR/3 search*):ti,ab) OR (((literature NEAR/3 review*):ti,ab) AND (search*:ti,ab OR database*:ti,ab OR 'data base*':ti,ab)) OR (('data extraction':ti,ab OR 'data source*':ti,ab) AND 'study selection':ti,ab) OR ('search strategy':ti,ab AND 'selection criteria':ti,ab) OR ('data source*':ti,ab AND 'data synthesis':ti,ab) OR medline:ab OR pubmed:ab OR embase:ab OR cochrane:ab OR (((critical OR rapid) NEAR/2 (review* OR overview* OR synthes*)):ti) OR ((((critical* OR rapid*) NEAR/3 (review* OR overview* OR synthes*)):ab) AND (search*:ab OR database*:ab OR 'data base*':ab)) OR metasynthes*:ti,ab OR 'meta synthes*':ti,ab	
#12	#9 AND #10 AND #11 AND ([english]/lim OR [dutch]/lim) AND [2013-2023]/py NOT ('conference abstract'/it OR 'editorial'/it OR 'letter'/it OR 'note'/it) NOT (('animal experiment'/exp OR 'animal model'/exp OR 'nonhuman'/exp) NOT 'human'/exp)	1129
#11	'conservative treatment'/exp OR 'physiotherapy'/exp OR physiotherap*:ti,ab,kw OR 'physio therap*':ti,ab,kw OR 'physical therap*':ti,ab,kw OR 'kinesiotherapy'/exp OR kinesiotherap*:ti,ab,kw OR kinesitherapeutic*:ti,ab,kw OR 'occupational therapy'/exp OR 'occupation* therap*':ti,ab,kw OR ergotherap*:ti,ab,kw OR 'conservative treatment*':ti,ab,kw OR nonsurg*:ti,ab,kw OR nonoperatic*:ti,ab,kw OR 'non-surg*':ti,ab,kw OR 'non-operati*':ti,ab,kw OR (((exercise* OR manual) NEAR/3 therap*):ti,ab,kw) OR 'exercise'/exp/mj OR 'rehabilitation'/exp OR 'rehabilitation medicine'/exp OR rehabilitat*:ti,ab,kw OR 'manipulative medicine'/exp OR 'manual therapist'/exp OR 'musculoskeletal manipulation'/exp OR manipulat*:ti,ab,kw OR 'mobilization'/exp OR mobilization:ti,ab,kw OR mobilisation:ti,ab,kw OR 'massage'/exp OR 'massage':ti,ab,kw OR 'stretching exercise'/exp OR 'muscle stretching'/exp OR 'stretching'/exp OR stretch*:ti,ab,kw OR 'resistance training'/exp OR (((resistance OR strength*) NEAR/3 (train* OR exercise*)):ti,ab,kw) OR 'injection'/exp OR inject*:ti,ab,kw OR 'nonsteroid antiinflammatory agent'/exp OR 'nonsteroid antiinflammatory agent*':ti,ab,kw OR nsaid*:ti,ab,kw	4349666
#10	'shoulder surgery'/exp OR 'rotator cuff repair'/exp OR ((('rotator cuff' OR 'shoulder' OR tendon) NEAR/5 (repair* OR reconstruct* OR surger* OR surgic* OR operation* OR operative)):ti,ab,kw) OR surgic*:ti,kw OR surger*:ti,kw OR operation*:ti,kw OR operative:ti,kw OR 'single row':ti,ab,kw OR 'double row':ti,ab,kw	1241204
#9	'rotator cuff injury'/exp/mj OR 'rotator cuff rupture'/exp/mj OR ((('rotator cuff' OR 'shoulder cuff' OR infraspinatus OR supraspinatus OR subscapularis) NEAR/3 (tear* OR lesion* OR rupture* OR tendinopath* OR laceration*)):ti,ab,kw) OR (('full-thickness':ti,ab,kw OR partial:ti,ab,kw) AND	13918

	'rotator cuff':ti,ab,kw) OR ('rotator cuff'/exp/mj AND ('rupture'/exp/mj OR 'injury'/mj OR 'laceration'/exp/mj))	

Ovid/Medline

#	Searches	Results
11	9 or 10	298
10	(6 and 8) not 9 **= RCT**	178
9	6 and 7 **= SR**	120
8	exp randomized controlled trial/ or randomized controlled trials as topic/ or random*.ti,ab. or rct?.ti,ab. or ((pragmatic or practical) adj "clinical trial*").ti,ab,kf. or ((non-inferiority or noninferiority or superiority or equivalence) adj3 trial*).ti,ab,kf.	1587608
7	meta-analysis/ or meta-analysis as topic/ or (metaanaly* or meta-analy* or metanaly*).ti,ab,kf. or systematic review/ or cochrane.jw. or (prisma or prospero).ti,ab,kf. or ((systemati* or scoping or umbrella or "structured literature") adj3 (review* or overview*)).ti,ab,kf. or (systemic* adj1 review*).ti,ab,kf. or ((systemati* or literature or database* or data-base*) adj10 search*).ti,ab,kf. or ((structured or comprehensive* or systemic*) adj3 search*).ti,ab,kf. or ((literature adj3 review*) and (search* or database* or data-base*)).ti,ab,kf. or (("data extraction" or "data source*") and "study selection").ti,ab,kf. or ("search strategy" and "selection criteria").ti,ab,kf. or ("data source*" and "data synthesis").ti,ab,kf. or (medline or pubmed or embase or cochrane).ab. or ((critical or rapid) adj2 (review* or overview* or synthes*)).ti. or (((critical* or rapid*) adj3 (review* or overview* or synthes*)) and (search* or database* or data-base*)).ab. or (metasynthes* or meta-synthes*).ti,ab,kf.	649325
6	limit 5 to ((english language or dutch) and yr="2013 -Current")	808
5	4 not (comment/ or editorial/ or letter/ or ((exp animals/ or exp models, animal/) not humans/))	1477
4	1 and 2 and 3	1634
3	exp Conservative Treatment/ or exp Physical Therapy Modalities/ or exp Exercise Therapy/ or exp Occupational Therapy/ or physiotherap*.ti,ab,kf. or 'physio therap*'.ti,ab,kf. or 'physical therap*'.ti,ab,kf. or kinesiotherap*.ti,ab,kf. or kinesitherapeutic*.ti,ab,kf. or 'occupation* therap*'.ti,ab,kf. or ergotherap*.ti,ab,kf. or 'conservative treatment'.ti,ab,kf. or nonsurg*.ti,ab,kf. or nonoperatic*.ti,ab,kf. or 'non-surg*'.ti,ab,kf. or 'non-operati*'.ti,ab,kf. or exp *Exercise/ or ((exercise or manual) adj3 therap*).ti,ab,kf. or exp Rehabilitation/ or exp "Physical and Rehabilitation Medicine"/ or rehabilitat*.ti,ab,kf. or exp Musculoskeletal Manipulations/ or manipulat*.ti,ab,kf. or mobilization.ti,ab,kf. or mobilisation.ti,ab,kf. or exp Massage/ or 'massage'.ti,ab,kf. or exp Muscle Stretching Exercises/ or stretch*.ti,ab,kf. or exp Resistance Training/ or ((resistance or strength*) adj3 (train* or exercise*)).ti,ab,kf. or exp Injections/ or inject*.ti,ab,kf. or exp Anti-Inflammatory Agents, Non-Steroidal/ or 'nonsteroid antiinflammatory agent*'.ti,ab,kf. or nsaid*.ti,ab,kf.	2267264

2	Rotator Cuff/su or Shoulder Joint/su or Acromion/su or Shoulder/su or (('rotator cuff' or 'shoulder' or tendon) adj5 (repair* or reconstruct* or surger* or surgic* or operation* or operative)).ti,ab,kf. or surgic*.ti,kf. or surger*.ti,kf. or operation*.ti,kf. or operative.ti,kf. or 'single row'.ti,ab,kf. or 'double row'.ti,ab,kf.	999872
1	*Rotator Cuff/ or exp Rotator Cuff Injuries/ or (('rotator cuff' or 'shoulder cuff' or infraspinatus or supraspinatus or subscapularis) adj3 (tear* or lesion* or rupture* or tendinopath* or laceration*)).ti,ab,kf. or (('full-thickness' or partial) and 'rotator cuff').ti,ab,kf.	13101

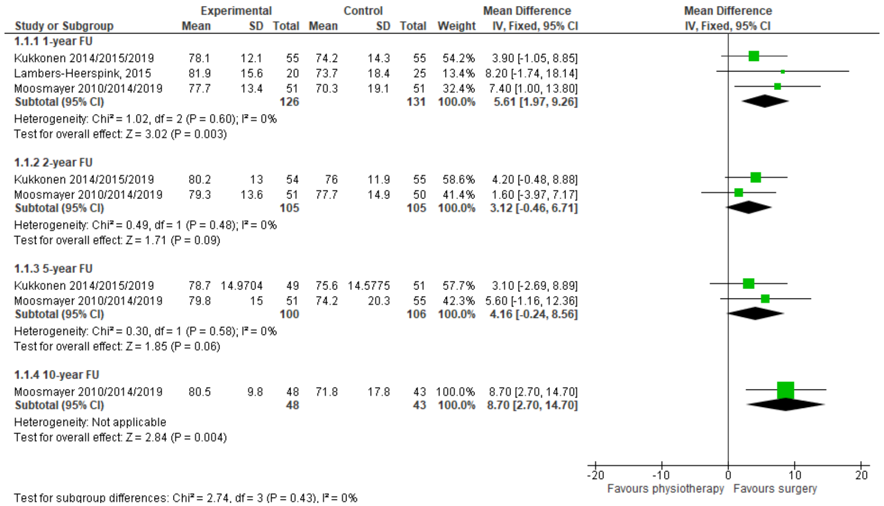

Evidence table module 5.1

Study reference	Study characteristics	Patient characteristics [2]	Intervention (I)	Comparison / control (C) [3]	Follow-up	Outcome measures and effect size [4]	Comments

Formaterat: Avstånd Efter: 0 pt, Radavstånd: enkelt

Formaterad tabell

Cederqvist, 2021<br><br>ClinicalTrials.gov, NCT00695981 and NCT00637013.	Type of study:<br>RCT<br><br>Setting and country:<br>Finland<br><br>Funding and conflicts of interest:<br>“Funding This work was supported by grants from the Academy of Finland (grant 12321/13.9.2007) 265646/17.4.2013) and National Competitive Research Funding of the University of Eastern Finland. None of the writers have any conflicts of interest relevant to this article. Competing interests None declared.”	*In this literature summary, the patients with full-thickness ruptures were included*<br>*Surgical group: 50/95 were full-thickness ruptures, of which 44 (88%%) in the supraspinatus*<br>*Non-surgical group: 48/95 were full-thickness ruptures, of which 44 (92%) in the supraspinatus*<br><br><u>Inclusion criteria</u>:<br>• Pain in abduction of the shoulder<br>• Age over 35 years<br>• Duration of symptoms at least 3 months<br>• Written informed consent by the participating subject<br>• Additional inclusion criteria<br>• Subacromial impingement without full-thickness tendon lesion<br>• Pain in two of the three isometric tests (0 or 30 degrees of abduction or external rotation)<br>• Subacromial injection of lidocaine significantly reduced pain<br>• Full-thickness tendon rupture<br>• Full-thickness rotator cuff rupture in one to three tendons documented with MRI arthrography<br><br><u>Exclusion criteria</u>:<br>• Previous surgery of the same shoulder<br>• High-energy trauma before symptoms<br>• Inflammatory arthritis	Repair: Arthroscopic or mini-open single-row surgical treatment of cuff repair<br>In surgery, patients without full-thickness tendon tears underwent arthroscopic SAD. Patients with full-thickness tears received rotator cuff repair with single-row technique, with one or more bone anchors, via either an arthroscopic or mini-open approach. When necessary, patients underwent acromioplasty, acromioclavicular joint resection or tenotomy of the long head of the biceps.<br><br>All patients followed a structured postoperative rehabilitation protocol (see online supplemental appendix).	Physiotherapy: cold pack + exercises + stretching, manual therapy, cross-friction massages<br>Patients randomised to non-surgical treatment continued the previously initiated rehabilitation programme. Unsuccessful non-surgical treatment was defined as severe pain or poor<br>subjective function in the shoulder during follow-up. These patients were offered a surgical intervention.<br><br>All patients followed a structured postoperative rehabilitation protocol (see<br>online supplemental appendix).	<u>Length of follow-up</u>:<br>2 years<br><br><u>Loss-to-follow-up & incomplete outcome data</u>:<br>Constant score<br>12 months<br>I: 18 (19%)<br>C: 18 (19%)<br>24 months:<br>I: 15 (16%)<br>C: 14 (15%)<br>VAS pain score<br>12 months<br>I: 18 (19%)<br>C: 19 (20%)<br>24 months:<br>I: 15 (16%)<br>C: 15 (16%)	**Complications**<br>“No patients required re-operation, and no serious adverse events were noted.”<br><br>**PROMS: function, strength, pain combined**<br>Constant score<br>FU 2 years, change from baseline<br>I: +20.0 (16.4 to 23.7)<br>C: +13.0 (9.4 to 16.7)<br>MD 7.0 (95%CI 1.8 to 12.2; p=0.008).<br><br>Calculated in RevMan<br>I: +20.0 (SD 16.1769), n=80<br>C: +13.0 (SD 16.1769), n=80<br>MD 7.00 (95% CI 1.99 to 12.01)<br><br>**Pain**<br>**VAS 0-100** change score from baseline, mean (95% CI)<br>FU 2 years<br>I: -37 (95% CI 31 to 43)<br>C: -24 (95% CI 18 to 30)<br>MD -13 (95% CI 5 to 22; p=0.002).<br><br>Calculated in RevMan:<br>I: -37.0 (SD 26.9616), n=80<br>C: -24.0 (SD 26.9616), n=80<br>MD 13.00 (95% CI -4.64 to 21.36)	

Formaterat: Avstånd Efter: 0 pt, Radavstånd: enkelt

Formaterat: Avstånd Efter: 0 pt, Radavstånd: enkelt

Formaterat: Avstånd Efter: 0 pt, Radavstånd: enkelt

		• Adhesive capsulitis<br>• Instability of the affected shoulder<br>• Severe glenohumeral or acromioclavicular joint osteoarthritis<br>• Cervical syndrome/radiculopathy<br>• Progressive cancer<br>• A too high risk for operation<br>• Any disease, social problem or other reason reducing the ability to co-operate and jeopardising informed consent<br>Irreparable rotator cuff tear on MRI arthrography<br><br><u>N total at baseline</u>:<br>Intervention: 50<br>Control: 48<br><br><u>Important prognostic factors[2]</u>:<br>*in general intervention groups; not reported for full-thickness only*<br>*age ± SD*<br>*I: 56 y (SD 8)*<br>*C: 56 y (SD 8)*<br>*Sex:*<br>*I: 50/95 (53%) M*<br>*C: 52/95 (55%) M*<br><br>Groups comparable at baseline.				Not included in plot, as change scores and difference scale (0-100) were used<br><br>**Patient satisfaction**<br>Not reported	
Kukkonen, 2014 / Kukkonen, 2015 a en b / Kukkonen 2019<br><br>NCT01116518	Type of study:<br>RCT<br><br>Setting and country:<br>three hospitals in Finland (Turku University Hospital, Kuopio University Hospital and Hatanpää Hospital)	Group 1 (surgery + physiotherapy) and group 3 (physiotherapy) were included in the current literature summary.<br><br><u>Inclusion criteria</u>:<br>• Age > 55 years	Repair: surgical rotator cuff repair + acromioplasty + immobilization in a sling for 3 weeks + physiotherapy<br><br>(group 3 in article)	Physiotherapy:<br><br>instructions<br>+ home exercises<br>+ 10 sessions of physiotherapy<br><br>(group 1 in article)	<u>Length of follow-up</u>:<br>60 months / 5 years<br>(mean follow-up period of 6.2 years)	**Complications**<br>"No treatment-related complications occurred in any group."	

Formaterat: Avstånd Efter: 0 pt, Radavstånd: enkelt

Formaterat: Avstånd Efter: 0 pt, Radavstånd: enkelt

	between October 2007 and December 2012; Finland<br><br>Funding and conflicts of interest:<br>Not mentioned explicitly; “No benefits in any form have been received or will be received from a commercial party related directly or indirectly to the subject of this article.”	• Atraumatic symptomatic supraspinatus tendon tear comprising < 75% of the tendon insertion and documented with MRI<br>• Full range of motion of the shoulder<br>• Written informed consent<br><br>Exclusion criteria:<br>• Age < 55 years<br>• History of trauma relating to the onset of symptoms<br>• massive tendon tear involving whole supra-spinatus tendon and/or combined tear of two to three tendons, i.e., supra-spinatus with infraspinatus or subscapularis tendon tear<br>• Stiffness of the glenohumeral joint (passive external rotation < 30° ± elevation < 120°)<br>• Glenohumeral osteoarthritis with present osteophytes in radiographs<br>• Systemic corticosteroid or antimetabolite medication<br>• Significant malignant, hematological, endocrine, metabolic, rheumatoid or gastrointestinal disease<br>• History of alcoholism, drug abuse, psycho-logical or other emotional problems that are likely to invalidate informed consent<br>• Previous surgery of same shoulder<br>• Patient refusal<br><br>N total at baseline:<br>Intervention: 60			Loss-to-follow-up & incomplete outcome data:<br>Intervention (group 3)<br>Baseline data: n=59 shoulders<br>Intervention: n=55<br>12m FU: 55<br>24m FU: 54<br>60m FU: 49<br><br>Control (group 1)<br>Baseline data: n=58 shoulders<br>Intervention: n=60<br>12m FU: 55<br>24m FU: 55<br>60m FU: 51	**PROMS: function, strength, pain combined**<br>*Constant score (range from 0 to 100 points: worst and best shoulder function)*<br>FU 1 year<br>I: 78.1 (SD 12.1), n=55<br>C: 74.2 (SD 14.3), n=55<br>FU 2 years<br>I: 80.2 (SD 13.0), n=54<br>C: 76 (SD 11.9), n=55<br>Calculated in RevMan:<br>MD 4.20 (95% CI -0.48 to 8.88)<br>*Mean change score baseline to 24 M (95% CI)*<br>I: 22.6 points (18.4 to 26.8 points)<br>C: 18.4 points (14.2 to<br>22.6 points)<br>FU 5 years<br>Mean (SD) score (95% CI)<br>I: 78.7 (74.4- 83.0)<br>C: 75.6 (71.5- 79.8)<br>Calculated in RevMan:<br>I: 78.7 (SD 14.9704), n=49<br>C: 75.6 (SD 14.5775), n=51<br>MD 3.10 (95% CI -2.69 to 8.89)<br>Mean change (95% CI)<br>I: 20.0 (15.0-24.9)<br>C: 18.5 (13.6-23.4)	

Formaterat: Avstånd Efter: 0 pt, Radavstånd: enkelt

Formaterat: Avstånd Efter: 0 pt, Radavstånd: enkelt

Formaterat: Avstånd Efter: 0 pt, Radavstånd: enkelt

		Control: 60<br><br><u>Important prognostic factors[2]</u>:<br>Female (n, %)<br>I: 26/55 (47%)<br>C: 31/55 (56%)<br><br>Mean (SD) age (yrs)<br>I: 65y (SD 6.0)<br>C: 65y (SD 5.8)<br><br>Groups comparable at baseline.				**Pain**<br>VAS pain scale 0-10; mean (SD)<br>FU 1 year<br>I: 0.9 (SD 2.1), N=55<br>C: 1.2 (SD 1.9), N=55<br>Calculated in RevMan:<br>MD -0.30 (95% CI -1.05 to 0.45)<br>FU 2 years<br>I: 0.6 (SD 1.6), N=54<br>C: 1.4 (SD 2.1), N=55<br>Calculated in RevMan:<br>MD -0.80 (95% CI -1.50 to -0.10)<br>*VAS change score for pain*<br>I: -2.0<br>C: -1.3<br>FU 5 years<br>*VAS pain scale; mean (95% CI)*<br>I: 0.62 (0.16-1.08)<br>C: 1.43 (0.98-1.88)<br>Calculated in RevMan<br>I: 0.62 (SD 1.6015), n=49<br>C: 1.43 (SD 1.6), n=51<br><br>MD -0.81 (95% CI -1.44 to -0.18)<br>Mean change in VAS pain score (95% CI)*<br>I: -1.85 (-2.66 to -1.04)<br>C: -1.55 (-2.35 to -0.75)<br><br>**Patient satisfaction**<br>*At the control visits patients were asked if they were satisfied or*	

						dissatisfied with the treatment outcome<br>FU 1 year<br>I: 95%<br>C: 87%<br>FU 2 years<br>I: 94%<br>C: 89%<br>FU 5 years<br>I: 91.8%<br>C: 88.2%	
Lambers Heerspink, 2015	Type of study:<br>RCT<br><br>Setting and country:<br>January 2009 and December 2012<br><br>Funding and conflicts of interest:<br>“This study received a grant from Anna Fonds. There was no involvement in data collection, data analysis, the preparation, or editing of the manuscript by Anna Fonds. The authors, their immediate families, and any research foundations with which they are affiliated have not received any financial payments or other benefits from any commercial entity related to the subject of this article.”	Inclusion criteria:<br>• degenerative, nontraumatic full-thickness rotator cuff tears<br><br>Exclusion criteria:<br>• traumatic onset of complaints<br>• previous surgical treatment of the shoulder,<br>• frozen shoulder,<br>• radiologic and symptomatic osteoarthritis of the glenohumeral (GH) or acromioclavicular joint, arthritis/rheumatoid arthritis,<br>• diabetes mellitus,<br>• cognitive disorders,<br>• neurologic disease affecting function of the upper extremity,<br>• language barriers impairing participation.<br><br>N total at baseline:<br>Intervention: 25<br>Control: 31<br><br>Important prognostic factors[2]:<br>*Age, SD:*<br>I: 60.8, 7.2<br>C: 60.5, 7.0	Repair: Surgical treatment<br><br>Surgery was scheduled within 6 weeks of inclusion and was done with the patient under general anaesthesia, supplemented with an interscalene brachial plexus block. The operation was performed in beach chair position using an anterolateral miniopen approach. The coraco-acromial ligament was detached from its insertion, and the subacromial bursa was excised. The anteroinferior part of the acromion was removed. The footprint of the rotator cuff on the greater tuberosity was debrided, and a bleeding bony bed was created. Side-to-side repair and repair augmented with bone anchors were performed depending on the shape of the rupture. A side-to-side repair was performed in 6 patients. The deltoid muscle was	Conservative treatment: Subacromial steroid infiltration, physiotherapy, and analgesic medication; further options: analgesic medication with NSAIDs, paracetamol, or tramadol)<br><br>Treatment in the conservative group consisted of subacromial steroid infiltration, physiotherapy, and analgesic medication. After inclusion, patients were given an infiltration in the subacromial space by a posterior approach. If the first infiltration gave no pain relief, a second infiltration was performed under radiologic or ultrasound guidance. The number of subacromial infiltrations was limited to a maximum of 3. Further conservative treatment options consisted of analgesic medication with nonsteroidal anti-inflammatory drugs (NSAIDs), paracetamol, or tramadol. Patients were referred to a physio-	Length of follow-up:<br>12m<br><br>Loss-to-follow-up & incomplete outcome data:<br>Intervention: 20/25 analysed<br>(1 moved, 4 excluded due to failed surgery)<br>Control: 25/31 analysed<br>(3 discontinued intervention, 1 death, 1 moved)	**Complications**<br>Not reported<br><br>**PROMS: function, strength, pain combined**<br>CMS, mean (SD)<br>FU 1 year<br>I: 81.9 (15.6); n=20<br>C: 73.7 (18.4), n=25<br>Calculated in RevMan:<br>MD: 8.20 (95% CI -1.74 to 18.14)<br>DSST<br>FU 1 year<br>I: 11.0 (2.8); n=20<br>C: 9.7 (3.6), n=25<br>Calculated in RevMan:<br>MD 1.30 (95% CI -1.35 to 3.95)<br><br>**Pain**<br>VAS pain score**, 0-10**; 0 represents no pain and restriction, and 10 the most likely pain and disability<br>FU 1 year<br>I: 2.2 (1.9), n=20<br>C: 3.2 (2.1), n=25<br>Calculated in RevMan:	

Formaterat: Avstånd Efter: 0 pt, Radavstånd: enkelt

Formaterat: Avstånd Efter: 0 pt, Radavstånd: enkelt

Formaterat: Avstånd Efter: 0 pt, Radavstånd: enkelt

Formaterat: Avstånd Efter: 0 pt, Radavstånd: enkelt

		Sex:<br>I: 60% M<br>C: 64.5% M<br><br>Groups comparable at baseline.	reattached to the acromion by transosseous refixation.<br><br>The repair in 14 patients was augmented using bone anchors.<br>The tear in 2 patients could not be repaired, and no rotator cuff rupture was found in 2 patients despite an MRI-supported diagnosis. These 4 patients were excluded for primary per-protocol analysis but were included in the intention-to-treat analysis.<br>After surgery, the patient wore a sling for 6 weeks. Patients were referred for physical therapy and treatment was commenced according to a standardized protocol.21 In the first 6 weeks, only passive movements were allowed. Passive GH movement was performed to prevent loss of mobility. The mobility of elbow and wrist was passively maintained. Circumduction exercises were allowed. After 6 weeks, active guided treatment was started and<br>was expanded to active treatment. Strength development was started 3 months postoperatively.	therapist. The Department of<br>Physical Therapy of Martini Hospital, Groningen, The Netherlands, developed a standardized physical therapy protocol for the conservative treatment of rotator cuff tears.21 In addition to explaining the cause of the symptoms and the rehabilitation protocol, the physiotherapist advised about activities of daily living (ADL). Passive GH and scapulothoracic movements were performed, and static and dynamic exercises were started. The aim of these exercises was to improve GH and scapulothoracic musculature. Poor posture was corrected. In weeks 4 to 6, exercises were gradually increased, and deltoid training was started. In weeks 6 to 12, rehabilitation was aimed at further optimization of mobility and strength regeneration<br>of the remaining cuff and deltoid. Physical therapy was continued until patients reached an optimum range of motion and an improvement in strength was achieved. Three patients were dissatisfied with the result of conservative treatment and a decision was made		MD -1.00 (95% CI -2.17 to 0.17)<br><br>**Patient satisfaction**<br>Not reported;<br>“Three patients were dissatisfied with the result of conservative treatment and a decision was made to perform rotator cuff repair (discontinued intervention). In 2 of these patients, data were available until 3 months after treatment and in the other patient until 6 months after treatment.”	

				to perform rotator cuff repair (discontinued intervention). In 2 of these patients, data were available until 3 months after treatment and in the other patient until 6 months after treatment.			
Moosmayer, 2010 / Moosmayer, 2014 / Moosmayer, 2019	Type of study:<br>RCT<br><br>Setting and country:<br>Single-centre; Norway<br><br>Funding and conflicts of interest: “One or more of the authors received payments or services, either directly or indirectly (i.e., via his or her institution), from a third party in support of an aspect of this work. None of the authors, or their institution(s), have had any financial relation-ship, in the thirty-six months prior to submission of this work, with any entity in the biomedical arena that could be perceived to influence or have the potential to influence what is written in this work. Also, no author has had any other relationships, or has engaged in any other activities, that could be perceived to influence or have the potential to influence what is written in this work. The complete Disclosures of Potential Conflicts of Interest submitted by authors are	<u>Inclusion criteria</u>:<br>• lateral shoulder pain at rest or with exercise,<br>• painful arc,<br>• positive impingement signs<br>• passive range of movement of at least 140° for abduction and flexion.<br>• full thickness tear by sonography and MRI,<br>• tear size of ≤ 3 cm on short and long axis ultrasound scans<br>• muscle atrophy on MRI not exceeding stage 2, according to classification of Thomazeau et al.<br>*Traumatic and atraumatic tears were included.*<br><br><u>Exclusion criteria</u>:<br>• age < 18 years,<br>• tears with absolute indication for surgery such as those involving substantial parts of the subscapularis tendon,<br>• presence of other local or systemic diseases affecting shoulder function,<br>• previous tendon surgery on the relevant shoulder,<br>• medical comorbidities<br>• inability to comply with follow-up	Repair: Surgical treatment of cuff repair (through a deltoid splitting approach, anteroinferior acromioplasty was performed)	Physiotherapy: physiotherapy and exercises	<u>Length of follow-up</u>:<br>10 years<br><br><u>Loss-to-follow-up & incomplete outcome data</u>:<br>Intervention:<br>12m FU: 51<br>24m FU: 51<br>5y FU: 51<br>10y: 48<br><br>Control:<br>12m FU: 51<br>24m FU: 50<br>5y FU: 55<br>10y: 43	**Complications**<br>Relevant complications according to guideline development group<br><br>Index shoulder, need for additional physiotherapy<br>I: 1 (within 2 years)<br>C: 3 (after 5 years)<br><br>Index shoulder, need for:<br>I: reoperation with acromioplasty and biceps tenotomy (n = 1)<br>C: Physiotherapy (n = 1)<br><br>C: Glenohumeral arthrosis, conservatively treated (n = 1)<br><br>**PROMS: function, strength, pain combined**<br><u>Constant score</u>,<br>*mean ± SD*<br>FU 1 year<br>I: 77.7 ± 13.4, n=52<br>C: 70.3 ± 19.1, n=51<br>Calculated in RevMan:	

Formaterat: Avstånd Efter: 0 pt, Radavstånd: enkelt

Formaterat: Avstånd Efter: 0 pt, Radavstånd: enkelt

Formaterat: Avstånd Efter: 0 pt, Radavstånd: enkelt

Formaterat: Avstånd Efter: 0 pt, Radavstånd: enkelt

	always provided with the online version of the article"<br><br>Source of Funding<br>"In support of the research for this manuscript, outside funding was given by the South-Eastern Norway Regional Health Authority. Funds were used to pay for salaries. The source of funding did not play a role in the investigation."	<u>N total at baseline</u>:<br>Intervention: 52<br>Control: 51<br><br><u>Important prognostic factors[2]</u>:<br>*age (range):*<br>*I: 59 (44 to 75)*<br>*C: 61 (46 to 75)*<br>*Sex:*<br>*I: 37/52, 71% M*<br>*C:36/51, 71 % M*<br><br>Groups comparable at baseline				MD 7.40 [1.00, 13.80]<br>FU 2 years<br>I: 79.3 ± 13.6<br>C: 77.7 ± 14.9<br>Calculated in RevMan:<br>MD 1.60 [-3.97, 7.17]<br>FU 5 years<br>I: 79.8 ± 15.0<br>C: 74.2 ± 20.3<br>Calculated in RevMan:<br>MD 5.60 [-1.16, 12.36]<br>FU 10 years<br>I: 80.5 ± 9.8<br>C: 71.8 ± 17.8<br>Calculated in RevMan:<br>MD 8.70 [2.70, 14.70]<br><br><u>ASES score</u> - *self-report section of the ASES score; 0-100; higher score indicating better functioning; mean ±* SD<br>FU 1 year<br>I: 93.6 ± 12.5<br>C: 83.6 ± 18.3<br>Calculated in RevMan:<br>MD 10.00 [3.92, 16.08]<br>FU 2 years<br>I: 93.1 ± 13.9<br>C: 88.0 ± 14.9<br>Calculated in RevMan:<br>MD 5.10 [-0.52, 10.72]<br>FU 5 years<br>I: 92.8 ± 13.3<br>C: 85.4 ± 21.0	

						Calculated in RevMan:<br>MD 7.40 [0.76, 14.04]<br>FU 10 years<br>I: 94.0 ± 9.5<br>C: 80.0 ± 20.2<br>Calculated in RevMan:<br>MD 14.00 [7.39, 20.61]<br><br>**Pain**<br><u>VAS</u> pain (cm); *mean ± SD*<br>FU 1 year<br>I: 0.5 ± 1.2<br>C: 1.6 ± 1.6<br>Calculated in RevMan:<br>MD -1.10 [-1.65, -0.55]<br>FU 2 years<br>I: 0.7 ± 1.5<br>C: 1.4 ± 1.4<br>Calculated in RevMan:<br>MD -0.70 [-1.27, -0.13]<br>FU 5 years<br>I: 0.6 ± 1.4<br>C: 1.6 ± 1.6<br>Calculated in RevMan:<br>MD -1.00 [-1.57, -0.43]<br>FU 10 years<br>I: 0.6 ± 1.3<br>C: 2.3 ± 2.4<br>Calculated in RevMan:<br>MD -1.70 [-2.51, -0.89]<br><br>**Patient satisfaction**<br>*VAS – scale: After 1, 5, and 10 years,*	

						patients had to answer the question "How satisfied are you with the treatment result of your shoulder?" on a visual analogue scale (VAS) ranging from 0 (very unsatisfied) to 10 (very satisfied).<br>FU 1 year<br>I: 9.0 (1.0 to 10.0)<br>C: 7.2 (0.0 to 10.0)<br>FU 2 years<br>I:<br>C:<br>FU 5 years<br>I: 9.2 cm<br>C: 8.3 cm<br>MD 1.0 cm [95% CI, 0.1 to 1.8 cm]; p = 0.03)<br>FU 10 years<br>I: 9.2 cm<br>C: 8.2 cm<br>MD 0.97 cm [95% CI, 0.13 to 1.82 cm]; p = 0.03)	

Module 5.2

What are the effects of tenotomy or tenodesis of the biceps tendon in patients with an isolated supraspinatus tendon tear and who have no pathology of the biceps tendon?

Patients	Patients with a repair of a supraspinatus tendon tear and without pathology of the intra-articular part of the biceps tendon
Intervention	Tenotomy or tenodesis of the biceps tendon
Control	No tenotomy or tenodesis
Outcomes	Complications, PROMS for pain and function, patient satisfaction, return to work or leisure

Embase.com

No.	Query	Results
#16	#13 OR #14 OR #15	241
#15	#9 AND #12 NOT (#13 OR #14) **= observationeel**	187
#14	#9 AND #11 NOT #13 **= RCT**	32
#13	#9 AND #10 **= SR**	22
#12	'clinical trial'/exp OR 'randomization'/exp OR 'single blind procedure'/exp OR 'double blind procedure'/exp OR 'crossover procedure'/exp OR 'placebo'/exp OR 'prospective study'/exp OR rct:ab,ti OR random*:ab,ti OR 'single blind':ab,ti OR 'randomised controlled trial':ab,ti OR 'randomized controlled trial'/exp OR placebo*:ab,ti OR 'major clinical study'/de OR 'clinical study'/de OR 'family study'/de OR 'longitudinal study'/de OR 'retrospective study'/de OR 'prospective study'/de OR 'comparative study'/de OR 'cohort analysis'/de OR ((cohort NEAR/1 (study OR studies)):ab,ti) OR (('case control' NEAR/1 (study OR studies)):ab,ti) OR (('follow up' NEAR/1 (study OR studies)):ab,ti) OR (observational NEAR/1 (study OR studies)) OR ((epidemiologic NEAR/1 (study OR studies)):ab,ti) OR (('cross sectional' NEAR/1 (study OR	16441812

	studies)):ab,ti) OR 'case control study'/de OR 'comparative study'/exp OR 'control group'/de OR 'controlled study'/de OR 'controlled clinical trial'/de OR 'crossover procedure'/de OR 'double blind procedure'/de OR 'phase 2 clinical trial'/de OR 'phase 3 clinical trial'/de OR 'phase 4 clinical trial'/de OR 'pretest posttest design'/de OR 'pretest posttest control group design'/de OR 'quasi experimental study'/de OR 'single blind procedure'/de OR 'triple blind procedure'/de OR (((control OR controlled) NEAR/6 trial):ti,ab,kw) OR (((control OR controlled) NEAR/6 (study OR studies)):ti,ab,kw) OR (((control OR controlled) NEAR/1 active):ti,ab,kw) OR 'open label*':ti,ab,kw OR (((double OR two OR three OR multi OR trial) NEAR/1 (arm OR arms)):ti,ab,kw) OR ((allocat* NEAR/10 (arm OR arms)):ti,ab,kw) OR placebo*:ti,ab,kw OR 'sham-control*':ti,ab,kw OR (((single OR double OR triple OR assessor) NEAR/1 (blind* OR masked)):ti,ab,kw) OR nonrandom*:ti,ab,kw OR 'non-random*':ti,ab,kw OR 'quasi-experiment*':ti,ab,kw OR crossover:ti,ab,kw OR 'cross over':ti,ab,kw OR 'parallel group*':ti,ab,kw OR 'factorial trial':ti,ab,kw OR ((phase NEAR/5 (study OR trial)):ti,ab,kw) OR ((case* NEAR/6 (matched OR control*)):ti,ab,kw) OR ((match* NEAR/6 (pair OR pairs OR cohort* OR control* OR group* OR healthy OR age OR sex OR gender OR patient* OR subject* OR participant*)):ti,ab,kw) OR ((propensity NEAR/6 (scor* OR match*)):ti,ab,kw) OR versus:ti OR vs:ti OR compar*:ti OR ((compar* NEAR/1 study):ti,ab,kw) OR (('major clinical study'/de OR 'clinical study'/de OR 'cohort analysis'/de OR 'observational study'/de OR 'cross-sectional study'/de OR 'multicenter study'/de OR 'correlational study'/de OR 'follow up'/de OR cohort*:ti,ab,kw OR 'follow up':ti,ab,kw OR followup:ti,ab,kw OR longitudinal*:ti,ab,kw OR prospective*:ti,ab,kw OR retrospective*:ti,ab,kw OR observational*:ti,ab,kw OR 'cross sectional*':ti,ab,kw OR cross?ectional*:ti,ab,kw OR multicent*:ti,ab,kw OR 'multi-cent*':ti,ab,kw OR consecutive*:ti,ab,kw) AND (group:ti,ab,kw OR groups:ti,ab,kw OR subgroup*:ti,ab,kw OR versus:ti,ab,kw OR vs:ti,ab,kw OR compar*:ti,ab,kw OR 'odds ratio*':ab OR 'relative odds':ab OR 'risk ratio*':ab OR 'relative risk*':ab OR 'rate ratio':ab OR aor:ab OR arr:ab OR rrr:ab OR ((('or' OR 'rr') NEAR/6 ci):ab)))	
#11	'randomized controlled trial'/exp OR random*:ti,ab OR (((pragmatic OR practical) NEAR/1 'clinical trial*'):ti,ab) OR ((('non inferiority' OR noninferiority OR superiority OR equivalence) NEAR/3 trial*):ti,ab) OR rct:ti,ab,kw	1839814
#10	'meta analysis'/exp OR 'meta analysis (topic)'/exp OR metaanaly*:ti,ab OR 'meta analy*':ti,ab OR metanaly*:ti,ab OR 'systematic review'/de OR 'cochrane database of systematic reviews'/jt OR prisma:ti,ab OR prospero:ti,ab OR (((systemati* OR scoping OR umbrella OR 'structured literature') NEAR/3 (review* OR overview*)):ti,ab) OR ((systemic* NEAR/1	733409

	review*):ti,ab) OR (((systemati* OR literature OR database* OR 'data base*') NEAR/10 search*):ti,ab) OR (((structured OR comprehensive* OR systemic*) NEAR/3 search*):ti,ab) OR (((literature NEAR/3 review*):ti,ab) AND (search*:ti,ab OR database*:ti,ab OR 'data base*':ti,ab)) OR (('data extraction':ti,ab OR 'data source*':ti,ab) AND 'study selection':ti,ab) OR ('search strategy':ti,ab AND 'selection criteria':ti,ab) OR ('data source*':ti,ab AND 'data synthesis':ti,ab) OR medline:ab OR pubmed:ab OR embase:ab OR cochrane:ab OR (((critical OR rapid) NEAR/2 (review* OR overview* OR synthes*)):ti) OR ((((critical* OR rapid*) NEAR/3 (review* OR overview* OR synthes*)):ab) AND (search*:ab OR database*:ab OR 'data base*':ab)) OR metasynthes*:ti,ab OR 'meta synthes*':ti,ab	
#9	#6 AND (#7 OR #8) AND ([english]/lim OR [dutch]/lim) AND [2013-2023]/py NOT ('conference abstract'/it OR 'editorial'/it OR 'letter'/it OR 'note'/it) NOT (('animal experiment'/exp OR 'animal model'/exp OR 'nonhuman'/exp) NOT 'human'/exp)	349
#8	('biceps brachii muscle'/exp OR biceps:ti,ab,kw) AND ('tenodesis'/exp OR tenodesis:ti,ab,kw)	1286
#7	('biceps brachii muscle'/exp OR biceps:ti,ab,kw) AND ('tenotomy'/exp OR tenotom*:ti,ab,kw)	729
#6	'rotator cuff injury'/exp/mj OR 'rotator cuff rupture'/exp/mj OR 'rotator cuff repair'/exp/mj OR ((('rotator cuff' OR 'shoulder cuff' OR infraspinatus OR supraspinatus OR subscapularis OR cuff) NEAR/3 (tear* OR lesion* OR rupture* OR tendinopath* OR laceration* OR repair)):ti,ab,kw) OR ('rotator cuff'/exp/mj AND ('rupture'/exp/mj OR 'injury'/mj OR 'laceration'/exp/mj)) OR 'supraspinatus muscle'/exp	16992

Ovid/Medline

#	Searches	Results
11	8 or 9 or 10	220
10	(4 and 7) not (8 or 9) **= observationeel**	153
9	(4 and 6) not 8 **= RCT**	40
8	4 and 5 **= SR**	27
7	exp clinical trial/ or randomized controlled trial/ or exp clinical trials as topic/ or randomized controlled trials as topic/ or Random Allocation/ or Double-Blind Method/ or Single-Blind Method/ or (clinical trial, phase i or clinical trial, phase ii or clinical trial, phase iii or clinical trial, phase iv or controlled clinical trial or randomized controlled trial or multicenter study or clinical trial).pt. or random*.ti,ab. or (clinic* adj trial*).tw. or ((singl* or doubl* or treb* or tripl*) adj (blind$3 or mask$3)).tw. or Placebos/ or placebo*.tw. or pidemiologic studies/ or case control studies/ or exp cohort studies/ or Controlled Before-After Studies/ or Case control.tw. or cohort.tw. or Cohort analy$.tw. or (Follow up adj (study or	8150482

	studies)).tw. or (observational adj (study or studies)).tw. or Longitudinal.tw. or Retrospective*.tw. or prospective*.tw. or consecutive*.tw. or Cross sectional.tw. or Cross-sectional studies/ or historically controlled study/ or interrupted time series analysis/ or Case-control Studies/ or clinical trial, phase ii/ or clinical trial, phase iii/ or clinical trial, phase iv/ or comparative study/ or control groups/ or controlled before-after studies/ or controlled clinical trial/ or double-blind method/ or historically controlled study/ or matched-pair analysis/ or single-blind method/ or (((control or controlled) adj6 (study or studies or trial)) or (compar* adj (study or studies)) or ((control or controlled) adj1 active) or "open label*" or ((double or two or three or multi or trial) adj (arm or arms)) or (allocat* adj10 (arm or arms)) or placebo* or "sham-control*" or ((single or double or triple or assessor) adj1 (blind* or masked)) or nonrandom* or "non-random*" or "quasi-experiment*" or "parallel group*" or "factorial trial" or "pretest posttest" or (phase adj5 (study or trial)) or (case* adj6 (matched or control*)) or (match* adj6 (pair or pairs or cohort* or control* or group* or healthy or age or sex or gender or patient* or subject* or participant*)) or (propensity adj6 (scor* or match*))).ti,ab,kf. or (confounding adj6 adjust*).ti,ab. or (versus or vs or compar*).ti. or ((exp cohort studies/ or epidemiologic studies/ or multicenter study/ or observational study/ or seroepidemiologic studies/ or (cohort* or 'follow up' or followup or longitudinal* or prospective* or retrospective* or observational* or multicent* or 'multi-cent*' or consecutive*).ti,ab,kf.) and ((group or groups or subgroup* or versus or vs or compar*).ti,ab,kf. or ('odds ratio*' or 'relative odds' or 'risk ratio*' or 'relative risk*' or aor or arr or rrr).ab. or (("OR" or "RR") adj6 CI).ab.))	
6	exp randomized controlled trial/ or randomized controlled trials as topic/ or random*.ti,ab. or rct?.ti,ab. or ((pragmatic or practical) adj "clinical trial*").ti,ab,kf. or ((non-inferiority or noninferiority or superiority or equivalence) adj3 trial*).ti,ab,kf.	1593790
5	meta-analysis/ or meta-analysis as topic/ or (metaanaly* or meta-analy* or metanaly*).ti,ab,kf. or systematic review/ or cochrane.jw. or (prisma or prospero).ti,ab,kf. or ((systemati* or scoping or umbrella or "structured literature") adj3 (review* or overview*)).ti,ab,kf. or (systemic* adj1 review*).ti,ab,kf. or ((systemati* or literature or database* or data-base*) adj10 search*).ti,ab,kf. or ((structured or comprehensive* or systemic*) adj3 search*).ti,ab,kf. or ((literature adj3 review*) and (search* or database* or data-base*)).ti,ab,kf. or (("data extraction" or "data source*") and "study selection").ti,ab,kf. or ("search strategy" and "selection criteria").ti,ab,kf. or ("data source*" and "data synthesis").ti,ab,kf. or (medline or pubmed or embase or cochrane).ab. or ((critical or rapid) adj2 (review* or overview* or synthes*)).ti. or (((critical* or rapid*) adj3 (review* or overview* or synthes*)) and (search* or database* or data-base*)).ab. or (metasynthes* or meta-synthes*).ti,ab,kf.	654023
4	limit 3 to ((english language or dutch) and yr="2013 -Current")	335
3	1 and 2	492

2	(biceps.ti,ab,kf. and (exp Tenotomy/ or tenotom*.ti,ab,kf.)) or (biceps.ti,ab,kf. and (exp Tenodesis/ or tenodesis*.ti,ab,kf.))	1188
1	*Rotator Cuff/ or exp Rotator Cuff Injuries/ or (('rotator cuff' or 'shoulder cuff' or infraspinatus or supraspinatus or subscapularis or cuff) adj3 (tear* or lesion* or rupture* or tendinopath* or laceration* or repair)).ti,ab,kf.	14066

Module 5.3

What are the prognostic factors for success or failure after a rotator cuff repair?

Patients	patients with a cuff tear
Intervention	prediction model that predicts success of operative treatment for patients with a cuff tear
Control	other prediction model or usual care
Outcomes	predictive value/model performance
Timing	before an operative treatment
Setting	during a consult with an orthopedic surgeon

Embase.com

No.	Query	Results
#10	#7 AND #8 AND #9 AND ([english]/lim OR [dutch]/lim) AND [2000-2023]/py NOT ('conference abstract'/it OR 'editorial'/it OR 'letter'/it OR 'note'/it) NOT (('animal experiment'/exp OR 'animal model'/exp OR 'nonhuman'/exp) NOT 'human'/exp)	604
#9	'area under the curve'/exp OR 'brier score'/exp OR 'computer prediction'/exp OR 'c statistic'/exp OR 'c statistics'/exp OR 'integrated discrimination improvement'/exp OR 'net reclassification improvement'/exp OR 'net reclassification index'/exp OR 'prediction'/exp OR 'predictive model'/exp OR 'predictive modeling'/exp OR 'predictive validity'/exp OR 'predictive value'/exp OR 'regression analysis'/exp OR 'statistical model'/exp OR 'area under the curve':ti,ab,kw OR 'brier score*':ti,ab,kw OR 'c statistic*' OR 'computer prediction':ti,ab,kw OR 'decision curve anal*':ti,ab,kw OR (('net reclassification' NEAR/2 (improvement OR index)):ti,ab,kw) OR (((predict* OR statistical*) NEAR/3 (model* OR validity OR value)):ti,ab,kw) OR 'proportional hazards model*':ti,ab,kw OR 'r square*':ti,ab,kw OR regression:ti,ab,kw OR predict*:ti OR multivariate:ti,ab,kw OR multivariab*:ti,ab,kw OR 'univariate analysis'/exp OR 'multivariate analysis'/exp OR univariate:ti,ab,kw	3395824

#8	'shoulder surgery'/exp/mj OR 'rotator cuff repair'/exp/mj OR ((('rotator cuff' OR 'shoulder' OR tendon) NEAR/5 (repair* OR reconstruct* OR reparability OR surger* OR surgic* OR operation* OR operative)):ti,ab,kw) OR surgic*:ti OR surger*:ti OR operation*:ti OR operative:ti OR 'single row':ti,ab,kw OR 'double row':ti,ab,kw OR 'surgery'/mj OR 'surgical patient'/mj OR 'surgical risk'/mj OR 'perioperative period'/mj	1150564
#7	'shoulder impingement syndrome'/exp/mj OR 'shoulder impingement syndrome':ti,ab,kw OR 'subacromial impingement syndrome':ti,ab,kw OR (('bursitis'/exp/mj OR bursitis:ti,ab,kw OR 'tendinitis'/exp/mj OR tendinitis:ti,ab,kw OR 'impingement':ti,ab,kw) AND ('shoulder'/exp/mj OR 'rotator cuff':ti,ab,kw OR acromion:ti,ab,kw OR acromial:ti,ab,kw OR subacromial:ti,ab,kw OR shoulder*:ti,ab,kw)) OR ((('rotator cuff' OR 'shoulder cuff' OR infraspinatus OR supraspinatus OR subscapularis) NEAR/3 (tear* OR lesion* OR rupture* OR tendinopath* OR laceration*)):ti,ab,kw) OR (('full-thickness':ti,ab,kw OR partial:ti,ab,kw) AND 'rotator cuff':ti,ab,kw) OR 'rotator cuff injury'/exp/mj OR 'rotator cuff rupture'/exp/mj OR ((('rotator cuff' OR 'shoulder cuff' OR infraspinatus OR supraspinatus OR subscapularis OR cuff) NEAR/3 (tear* OR lesion* OR rupture* OR tendinopath* OR laceration*)):ti,ab,kw) OR ('rotator cuff'/exp/mj AND ('rupture'/exp/mj OR 'injury'/mj OR 'laceration'/exp/mj)) OR 'supraspinatus muscle'/exp	19135

Ovid/Medline

#	Searches	Results
6	limit 5 to ((english language or dutch) and yr="2000 -Current")	685
5	4 not (comment/ or editorial/ or letter/ or ((exp animals/ or exp models, animal/) not humans/))	732
4	1 and 2 and 3	750
3	Area Under Curve/ or exp Forecasting/ or "Predictive Value of Tests"/ or exp Multivariate Analysis/ or exp Regression Analysis/ or exp Models, Statistical/ or area under the curve.ti,ab,kf. or brier score*.ti,ab,kf. or c statistic*.ti,ab,kf. or computer prediction.ti,ab,kf. or decision curve anal*.ti,ab,kf. or (net reclassification adj2 (improvement or index)).ti,ab,kf. or ((predict* or statistical*) adj3 (model* or validity or value)).ti,ab,kf. or proportional hazards model*.ti,ab,kf. or r square*.ti,ab,kf. or regression.ti,ab,kf. or predict*.ti. or multivaria*.ti,ab,kf.	2394116
2	Rotator Cuff/su or Shoulder Joint/su or Acromion/su or Shoulder/su or (('rotator cuff' or 'shoulder' or tendon) adj5 (repair* or reconstruct* or surger* or surgic* or operation* or operative)).ti,ab,kf. or surgic*.ti,kf. or surger*.ti,kf. or operation*.ti,kf. or operative.ti,kf. or 'single row'.ti,ab,kf. or 'double row'.ti,ab,kf.	1010948

1	*Rotator Cuff/ or exp Rotator Cuff Injuries/ or (('full-thickness' or partial) and 'rotator cuff').ti,ab,kf. or (('rotator cuff' or 'shoulder cuff' or infraspinatus or supraspinatus or subscapularis or cuff) adj3 (tear* or lesion* or rupture* or tendinopath* or laceration*)).ti,ab,kf. or *Shoulder Impingement Syndrome/ or 'shoulder impingement syndrome'.ti,ab,kf. or 'subacromial impingement syndrome'.ti,ab,kf. or ((exp *bursitis/ or exp *Tendinopathy/ or bursitis.ti,ab,kf. or tendinitis.ti,ab,kf. or 'impingement'.ti,ab,kf.) and (exp *Shoulder/ or 'rotator cuff'.ti,ab,kf. or acromion.ti,ab,kf. or acromial.ti,ab,kf. or subacromial.ti,ab,kf. or shoulder*.ti,ab,kf.))	18674

5.4

What is the effectiveness of early mobilization (up to max. 3 weeks) in which possibly a sling can be used as follow-up treatment, versus long-term (6 weeks) immobilization in patients where the supraspinatus tendon is repaired?

Patients	patients in whom the supraspinatus tendon is repaired
Intervention	early mobilization whereby a sling can be used for the first few weeks (up to a maximum of 3) (short-term immobilization to achieve early mobilization. Immediate practice with pendulum exercises after surgery can be performed/is allowed).
Control	long-term immobilization: 6 weeks
Outcomes	function (including post-op stiffness), pain, frozen shoulder (postoperative stiffness), complications (re-ruptures), patient satisfaction, return to work/sport.

No.	Query	Results
#1	'rotator cuff repair'/exp OR ((('rotator cuff' OR 'shoulder cuff' OR supraspinatus) NEAR/3 (repair* OR reconstruct* OR arthroscop* OR arthroendoscop* OR surg*)):ti,ab,kw) OR (('rotator cuff rupture'/exp OR 'rotator cuff injury'/de OR 'supraspinatus tendon tear'/exp OR 'supraspinatus tear'/exp OR 'supraspinatus tendon rupture'/exp OR (('rotator cuff'/exp OR 'supraspinatus muscle'/exp OR 'supraspinatus tendon'/exp OR 'supraspinatus muscle tendon'/exp OR 'supraspinatus'/exp) AND ('rupture'/exp OR 'injury'/de OR 'laceration'/exp)) OR	14412

	((('rotator cuff' OR 'shoulder cuff' OR supraspinatus) NEAR/3 (ruptur* OR tear* OR torn OR lesion* OR laceration*)):ti,ab,kw)) AND ('arthroscopy'/de OR 'shoulder arthroscopy'/exp OR 'surgery'/exp OR 'surgical patient'/exp OR 'perioperative period'/exp OR 'postoperative period'/exp OR 'postoperative care'/exp OR 'surgery'/lnk OR surgic*:ti,ab,kw OR surger*:ti,ab,kw OR operation*:ti,ab,kw OR operative:ti,ab,kw OR arthroscop*:ti,ab,kw OR perisurg*:ti,ab,kw OR perioperati*:ti,ab,kw OR postsurg*:ti,ab,kw OR 'post surg*':ti,ab,kw OR postoperati*:ti,ab,kw OR 'post operati*':ti,ab,kw OR intraoperati*:ti,ab,kw))	
#2	'mobilization'/exp OR 'immobilization'/exp OR 'immobilization device'/de OR 'limb immobilizer'/exp OR 'shoulder sling'/exp OR 'arm sling'/exp OR 'shoulder brace'/exp OR 'brace'/de OR 'orthosis'/de OR mobiliz*:ti,ab,kw OR mobilis*:ti,ab,kw OR immobilis*:ti,ab,kw OR immobiliz*:ti,ab,kw OR sling*:ti,ab,kw OR polysling*:ti,ab,kw OR orthosis:ti,ab,kw OR orthoses:ti,ab,kw OR orthotic:ti,ab,kw OR orthesis:ti,ab,kw OR brace*:ti,ab,kw OR bracing:ti,ab,kw OR (((early OR accelerat* OR late OR delay* OR conservative) NEAR/3 (motion OR movement OR rehabilitat*)):ti,ab,kw)	391152
#3	#1 AND #2	949
#4	#3 NOT ('conference abstract'/it OR 'editorial'/it OR 'letter'/it OR 'note'/it) NOT (('animal'/exp OR 'animal experiment'/exp OR 'animal model'/exp OR 'nonhuman'/exp) NOT 'human'/exp) NOT (('adolescent'/exp OR 'child'/exp OR adolescent*:ti,ab,kw OR child*:ti,ab,kw OR schoolchild*:ti,ab,kw OR infant*:ti,ab,kw OR girl*:ti,ab,kw OR boy*:ti,ab,kw OR teen:ti,ab,kw OR teens:ti,ab,kw OR teenager*:ti,ab,kw OR youth*:ti,ab,kw OR pediatr*:ti,ab,kw OR paediatr*:ti,ab,kw OR puber*:ti,ab,kw) NOT ('adult'/exp OR 'aged'/exp OR 'middle aged'/exp OR adult*:ti,ab,kw OR man:ti,ab,kw OR men:ti,ab,kw OR woman:ti,ab,kw OR women:ti,ab,kw))	748

#5	#4 AND [2013-2023]/py	496
#6	'meta analysis'/exp OR 'meta analysis (topic)'/exp OR metaanaly*:ti,ab OR 'meta analy*':ti,ab OR metanaly*:ti,ab OR 'systematic review'/de OR 'cochrane database of systematic reviews'/jt OR prisma:ti,ab OR prospero:ti,ab OR (((systemati* OR scoping OR umbrella OR 'structured literature') NEAR/3 (review* OR overview*)):ti,ab) OR ((systemic* NEAR/1 review*):ti,ab) OR (((systemati* OR literature OR database* OR 'data base*') NEAR/10 search*):ti,ab) OR (((structured OR comprehensive* OR systemic*) NEAR/3 search*):ti,ab) OR (((literature NEAR/3 review*):ti,ab) AND (search*:ti,ab OR database*:ti,ab OR 'data base*':ti,ab)) OR (('data extraction':ti,ab OR 'data source*':ti,ab) AND 'study selection':ti,ab) OR ('search strategy':ti,ab AND 'selection criteria':ti,ab) OR ('data source*':ti,ab AND 'data synthesis':ti,ab) OR medline:ab OR pubmed:ab OR embase:ab OR cochrane:ab OR (((critical OR rapid) NEAR/2 (review* OR overview* OR synthes*)):ti) OR ((((critical* OR rapid*) NEAR/3 (review* OR overview* OR synthes*)):ab) AND (search*:ab OR database*:ab OR 'data base*':ab)) OR metasynthes*:ti,ab OR 'meta synthes*':ti,ab	970896
#7	'clinical trial'/exp OR 'randomization'/exp OR 'single blind procedure'/exp OR 'double blind procedure'/exp OR 'crossover procedure'/exp OR 'placebo'/exp OR 'prospective study'/exp OR rct:ab,ti OR random*:ab,ti OR 'single blind':ab,ti OR 'randomised controlled trial':ab,ti OR 'randomized controlled trial'/exp OR placebo*:ab,ti	3896362
#8	#5 AND #6 = SR	55
#9	#5 AND #7 NOT #8 = RCT	106
#10	#8 OR #9	161

Ovid/Medline

#	Searches	Results
1	(('rotator cuff' or 'shoulder cuff' or supraspinatus) adj3 (repair* or reconstruct* or arthroscop* or arthroendoscop* or surg*)).ti,ab,kf. or ((exp Rotator Cuff Injuries/ or (exp Rotator Cuff/ and (Rupture/ or exp Lacerations/ or exp Tendon Injuries/)) or (('rotator cuff' or 'shoulder cuff' or supraspinatus) adj3 (ruptur* or tear* or torn or lesion* or laceration*)).ti,ab,kf.) and (exp Arthroscopy/ or exp Surgical Procedures, Operative/ or exp Specialties, Surgical/ or exp Perioperative Care/ or exp Perioperative Period/ or Postoperative Care/ or exp Postoperative Period/ or su.fs. or surgic*.ti,ab,kf. or surger*.ti,ab,kf. or operation*.ti,ab,kf. or operative.ti,ab,kf. or arthroscop*.ti,ab,kf. or perisurg*.ti,ab,kf. or perioperati*.ti,ab,kf. or postsurg*.ti,ab,kf. or 'post surg*'.ti,ab,kf. or postoperati*.ti,ab,kf. or 'post operati*'.ti,ab,kf. or intraoperati*.ti,ab,kf.))	10747
2	exp Immobilization/ or exp Orthotic Devices/ or mobiliz*.ti,ab,kf. or mobilis*.ti,ab,kf. or immobilis*.ti,ab,kf. or immobiliz*.ti,ab,kf. or sling*.ti,ab,kf. or polysling*.ti,ab,kf. or orthosis.ti,ab,kf. or orthoses.ti,ab,kf. or orthotic.ti,ab,kf. or orthesis.ti,ab,kf. or brace*.ti,ab,kf. or bracing.ti,ab,kf. or ((early or accelerat* or late or delay* or conservative) adj3 (motion or movement or rehabilitat*)).ti,ab,kf.	290280
3	1 and 2	469
4	3 not (comment/ or editorial/ or letter/) not ((exp animals/ or exp models, animal/) not humans/) not ((Adolescent/ or Child/ or Infant/ or adolescen*.ti,ab,kf. or child*.ti,ab,kf. or schoolchild*.ti,ab,kf. or infant*.ti,ab,kf. or girl*.ti,ab,kf. or boy*.ti,ab,kf. or teen.ti,ab,kf. or teens.ti,ab,kf. or teenager*.ti,ab,kf. or youth*.ti,ab,kf. or pediatr*.ti,ab,kf. or paediatr*.ti,ab,kf. or puber*.ti,ab,kf.) not (Adult/ or	425

	adult*.ti,ab,kf. or man.ti,ab,kf. or men.ti,ab,kf. or woman.ti,ab,kf. or women.ti,ab,kf.))	
5	limit 4 to yr="2013 -Current"	261
6	meta-analysis/ or meta-analysis as topic/ or (metaanaly* or meta-analy* or metanaly*).ti,ab,kf. or systematic review/ or cochrane.jw. or (prisma or prospero).ti,ab,kf. or ((systemati* or scoping or umbrella or "structured literature") adj3 (review* or overview*)).ti,ab,kf. or (systemic* adj1 review*).ti,ab,kf. or ((systemati* or literature or database* or data-base*) adj10 search*).ti,ab,kf. or ((structured or comprehensive* or systemic*) adj3 search*).ti,ab,kf. or ((literature adj3 review*) and (search* or database* or data-base*)).ti,ab,kf. or (("data extraction" or "data source*") and "study selection").ti,ab,kf. or ("search strategy" and "selection criteria").ti,ab,kf. or ("data source*" and "data synthesis").ti,ab,kf. or (medline or pubmed or embase or cochrane).ab. or ((critical or rapid) adj2 (review* or overview* or synthes*)).ti. or (((critical* or rapid*) adj3 (review* or overview* or synthes*)) and (search* or database* or data-base*)).ab. or (metasynthes* or meta-synthes*).ti,ab,kf.	700911
7	exp clinical trial/ or randomized controlled trial/ or exp clinical trials as topic/ or randomized controlled trials as topic/ or Random Allocation/ or Double-Blind Method/ or Single-Blind Method/ or (clinical trial, phase i or clinical trial, phase ii or clinical trial, phase iii or clinical trial, phase iv or controlled clinical trial or randomized controlled trial or multicenter study or clinical trial).pt. or random*.ti,ab. or (clinic* adj trial*).tw. or ((singl* or doubl* or treb* or tripl*) adj (blind$3 or mask$3)).tw. or Placebos/ or placebo*.tw.	2645320
8	5 and 6 = SR	40
9	(5 and 7) not 8 = RCT	49

10	8 or 9	89

Evidence table module 5.4

Study reference	Study characteristics	Patient characteristics [2]	Intervention (I)	Comparison / control (C) [3]	Follow-up	Outcome measures and effect size [4]	Comments
Düzgün, 2014	Type of study:<br>a prospective, clinical study (quasi-randomly assigned into accelerated groups)<br><br>Setting and country:<br>Sports Physiotherapy Unit, University, Turkey.<br><br>Funding and conflicts of interest:<br>Written informed consent (Hacettepe University Ethics Committee; FON 05/15-30) was obtained from all patients.<br><br>No conflict of interest.	Inclusion criteria:<br>Patients with stage 2 or 3 rotator cuff tear determined by MRI whom underwent arthroscopic rotator cuff repair for a full-thickness tear were included in the study.<br><br>Exclusion criteria:<br>Patients presenting with a central nervous system disorder or a peripheral nerve disorder, who were not willing to cooperate with the rehabilitation duration, or who self-reported psychological disorder, were excluded.<br><br>N total at baseline: 40<br>Intervention: 19<br>Control: 21<br><br>Important prognostic factors:	Patients were quasi-randomly assigned to one of the two groups based on their year of enrolment in the study. The 19 patients presenting in the 1st year were placed in the accelerated (ACCEL) protocol group (17 females, 2 males).<br>Surgery was performed according to the procedure described by Düzgün et al.<br><br>Patients enrolled in the ACCEL group were given 6 weeks of preoperative rehabilitation. The ACCEL protocol was initiated at the 2nd postoperative week and included soft tissue mobilization for the scapulothoracic and glenohumeral joint along with motion exercises. Active ROM exercises with scapular plane elevation, flexion and	21 patients were enrolled in the 2nd year were placed in the slow (SLOW) protocol group (17 females, 4 males).<br>Surgery was performed according to the procedure described by Düzgün et al.<br><br>In the SLOW group, soft tissue mobilization for the scapulothoracic and glenohumeral joint along with passive ROM exercises were initiated at the 4th postoperative week. Active ROM in scapular plane elevation, flexion and abduction was initiated at the 6th week and light resistive elastic resistance exercises at the 8th week. The protocol was applied 3 days a week for 14 weeks.	Length of follow-up:<br>at the 3rd, 5th, 8th, 12th, and 24th postoperative week<br><br>Loss-to-follow-up:<br>Intervention: 1 (loss to follow-up since 24 week follow-up).<br>Control: 1 (loss to follow-up since 24 week follow-up).<br><br>Incomplete outcome data:<br>Intervention: 0<br>Control: 0	**1. Function (including post-op stiffness)**<br><br>**1.1. Function at 6 weeks post op**<br>Not reported<br><br>**1.2. Function at 3 months post op**<br>Not reported<br><br>**1.3. Function at 6 months post op**<br>Not reported<br><br>**1.4. Function at 12 months post op**<br>Not reported<br><br>**2. Pain**<br>Not reported<br>**2.1. Pain at 6 weeks post op**<br>Not reported<br>**2.2. Pain at 3 months post op**<br>Not reported<br>**2.3. Pain at 6 months post op**	Authors' conclusion:<br>In conclusion, in both early and late initiation of the rehabilitation protocol, ROM eventually reaches normal values by 6 months.<br><br>Patients in the current study received the same rehabilitation protocol with passive, active, and resistive exercises introduced at either the earlier (ACCEL) or later (SLOW) postoperative period. All patients demonstrated improvement in ROM through the course of rehabilitation.<br><br>-No baseline assessment/measurement (makes it difficult to determine whether the protocol or the individual in the groups accounted for the differences observed).

Formaterat: Avstånd Efter: 0 pt, Radavstånd: enkelt

Formaterad tabell

Formaterat: Avstånd Efter: 0 pt, Radavstånd: enkelt

			All patients had non-traumatic degenerative tears.<br><br>*Age (years):*<br>*Mean ± SD*<br>*I: 57.68 ± 7.8*<br>*C: 57.2 ± 10.1*<br><br>*Sex:*<br>*I: 17 females, 2 males*<br>*C: 17 females, 4 males*<br><br>Groups comparable at baseline? yes	abduction was initiated at the 3rd week as long as the patient reported no pain at rest with their surgically repaired shoulder. Active exercises were delayed by 1 week in 1 patient due to pain upon removal of the support which later resolved. Light resistive elastic resistance (Thera-Band, red color-coded) exercises were initiated at the 4th postoperative week. The ACCEL protocol was applied 3 days a week for 6 weeks.			Not reported<br>**2.4. Pain at 12 months post op**<br>Not reported<br><br>**3. Frozen shoulder**<br>**3.1. Frozen shoulder at 6 weeks post op**<br>Not reported<br>**3.2. Frozen shoulder at 3 months post op**<br>Not reported<br>**3.3. Frozen shoulder at 6 months post op**<br>Not reported<br>**3.4. Froze shoulder at 12 months post op.**<br><br>**4. Complications/adverse events**<br>Not reported<br>**4.1. Complications at 6 weeks post op**<br>Not reported<br>**4.2. Complications at 3 months post op**<br>Not reported<br>**4.3. Complications at 6 months post op**<br>Not reported<br>**4.4. Complications at 12 months post op**<br>Surgical complications or adverse responses throughout the duration of the rehabilitation protocol, NO (N, %):<br>I: 19 (100%)<br>C: 21 (100%)<br><br>**5. Patient satisfaction**<br>Not reported<br>**5.1. Patient satisfaction at 6 weeks post op**<br>Not reported	

						**<u>5.2. Patient satisfaction at 3 months post op</u>**<br>Not reported<br>**<u>5.3. Patient satisfaction at 6 months post op</u>**<br>Not reported<br>**<u>5.4. Patient satisfaction at 12 months post op</u>**<br>Not reported<br><br>**<u>6. Return to work/sport</u>**<br>Not reported<br>**<u>6.1. Return to work/sport at 6 weeks post op</u>**<br>Not reported<br>**<u>6.2. Return to work/sport at 3 months post op</u>**<br>Not reported<br>**<u>6.3. Return to work/sport at 6 months post op</u>**<br>Not reported<br>**<u>6.4. Return to work/sport at 12 months post op</u>**<br>Not reported	
Jenssen 2018	<u>Type of study:</u><br>a prospective, randomized, non-inferiority trial.<br><br><u>Setting and country:</u><br>Patients were recruited between 2013 to 2015. Patients from a single orthopaedic practice of 5 shoulder surgeons were enrolled in this trial. Further not specified: first author was affiliated with Oslo University Hospital in Oslo, Norway.<br><br><u>Funding and conflicts of interest:</u>	<u>Inclusion criteria</u>:<br>Eligible patients had repairable full-thickness RC tears =< 3 cm affecting the supraspinatus or upper infraspinatus tendon. The patients had dysfunctional and painful shoulders due to a chronic RC tear nonresponsive to exercise therapy for a minimum of 3 months or an acute on chronic RC tear.<br><br><u>Exclusion criteria</u>:<br>-irreparable cuff tears, -tears >= 3 cm, -full-thickness subscapularis tendon tear, -adhesive capsulitis, -concomitant labral repair, -revision	Surgical procedures to repair RC, were performed by specialized shoulder surgeons.<br>Surgical procedures were the same in both groups.<br>RC repair was performed with an arthroscopic, single-row, repair technique using 1 or 2 triple-loaded healicoil PK suture anchors after debridement and micro fracture of the tendon footprint.<br><br>Had an early active range of motion starting at 3 weeks. Had a simple sling for 3 weeks.<br>The patients were told to keep the sling on day and night and to take it off 3 times	Surgical procedures to repair RC, were performed by specialized shoulder surgeons.<br>Surgical procedures were the same in both groups.<br>RC repair was performed with an arthroscopic, single-row, repair technique using 1 or 2 triple-loaded healicoil PK suture anchors after debridement and micro fracture of the tendon footprint.<br><br>Had a delayed active range of motion starting 6 weeks after surgery. Had a brace with a small abduction pillow with the arm in neutral position for 6 weeks after surgery. The patients	<u>Length of follow-up</u>:<br>1 year follow-up<br><br><u>Loss-to-follow-up</u>:<br>2 patients loss to follow-up after 1 year: one withdrew and one was terminally ill.<br><br>Intervention:<br>Control:<br><br><u>Incomplete outcome data</u>:<br>Intervention:<br>Control:	**<u>1. Function (including post-op stiffness)</u>**<br><br>**<u>1.1. Function at 6 weeks post op</u>**<br>Not reported<br><br>**<u>1.2. Function at 3 months post op</u>**<br>*Constant Murley Score*<br>I: 41 ± 23<br>C: 38 ± 19<br>MD: -3, 95% CI -5 to -10.<br><br>*WORC*<br>I: 58 ± 20<br>C: 59 ± 18<br>MD: -1, 95% CI -8 to 6.<br><br>**<u>1.3. Function at 6 months post op</u>**<br>*CM*	<u>Authors' conclusion:</u><br>Clinical trial registered<br><br>RC repair resulted in improved postoperative shoulder function, regardless of whether the shoulder was immobilized for 3 or 6 weeks.<br><br>Regarding WORC index: a difference between the 2 groups of 13 points (out of a total of 100) on the WORC index after 12 months was considered clinically relevant.

Formaterat: Avstånd Efter: 0 pt, Radavstånd: enkelt

		Not reported	repair, -fatty muscle infiltration of the RC > 50%, -shoulder joint osteoarthritis, -diabetes mellitus, and -systemic inflammatory disorders.<br><br>*In both groups, patients reported the duration of symptoms to be longer than 1 year on average.*<br><br>N total at baseline: 120<br>Intervention: 60<br>Control: 58<br><br>Important prognostic factors:<br><br>*Age, mean (min-max) years*<br>I: 56 (34-69)<br>C: 55 (34-73)<br><br>*Gender (male/female)*<br>I: 37/23<br>C: 32/26<br><br>RC tear associated with a previous trauma to the shoulder:<br>I: 33 patients<br>C: 26 patients<br><br>Groups comparable at baseline?<br>Yes	a day to perform pendulum exercises.<br><br>All patients were allowed active ROM of elbow and hand and passive ROM of the shoulder joint from day 1. An in-house physiotherapist educated the patients before they were discharged, and they were advised to visit their own physiotherapist for 2 to 3 times a week.<br>A report regarding advised rehabilitation after RC surgery was given to the patients' physiotherapist, and individual adjustments were made at the 6-weeks follow-up visit in the orthopaedic clinic.<br><br>Both groups were not allowed active ROM with resisted loading (lifting anything greater than the weight of the arm) for the first 3 months after surgery.	were told to keep the brace on day and night and to take it off 3 times a day to perform pendulum exercises. Some asked to remove the brace when sitting because it was pain relieving – however not reported whether patients did so.<br><br>All patients were allowed active ROM of elbow and hand and passive ROM of the shoulder joint from day 1. An in-house physiotherapist educated the patients before they were discharged, and they were advised to visit their own physiotherapist for 2 to 3 times a week.<br>A report regarding advised rehabilitation after RC surgery was given to the patients' physiotherapist, and individual adjustments were made at the 6-weeks follow-up visit in the orthopaedic clinic.<br><br>Both groups were not allowed active ROM with resisted loading (lifting anything greater than the weight of the arm) for the first 3 months after surgery.		I: 68 ± 28<br>C: 71 ± 25<br>MD: -3, 95% CI -12 to -7.<br><br>*WORC*<br>I: 75 ± 22<br>C: 77 ± 18<br>MD: -2, 95% CI -9 to -5.<br><br>**1.4. Function at 12 months post op**<br>*Constant Murley score*<br>I: 86 ± 27<br>C: 90 ± 23<br>MD: -4, 95% CI -13 to 5.<br><br>*Western Ontario Rotator Cuff (WORC) index.*<br>I: 83 (SD ± 19)<br>C: 87 (SD ± 14)<br>MD: -4, 95% CI -10 to -3.<br><br>**2. Pain**<br>Not reported<br>**2.1. Pain at 6 weeks post op**<br>Not reported<br>**2.2. Pain at 3 months post op**<br>Not reported<br>**2.3. Pain at 6 months post op**<br>Not reported<br>**2.4. Pain at 12 months post op**<br>Not reported<br><br>**3. Frozen shoulder**<br>Not reported<br>**3.1. Frozen shoulder 6 weeks post op**<br>Not reported<br>**3.2. Frozen shoulder 3 months post op**<br>Not reported	

							**<u>3.3. Frozen shoulder 6 months post op</u>**<br>Not reported<br>**<u>3.4. Frozen shoulder 12 months post op</u>**<br>Not reported<br><br>**<u>4. Complications/adverse events</u>**<br>**<u>4.1. Complications/adverse events 6 weeks post op</u>**<br>Not reported<br>**<u>4.2. Complications/adverse events 3 months post op</u>**<br>Not reported<br>**<u>4.3. Complications/adverse events 6 months post op</u>**<br>Not reported<br>**<u>4.4. Complications at 12 months post op</u>**<br>*Corticosteroid injections because of postoperative capsulitis*<br>I: 2/60<br>C: 0/58<br><br>**<u>5. Patient satisfaction</u>**<br>Not reported<br>**<u>5.1. Patient satisfaction at 6 weeks post op</u>**<br>Not reported<br>**<u>5.2. Patient satisfaction at 3 months post op</u>**<br>Not reported<br>**<u>5.3. Patient satisfaction at 6 months post op</u>**<br>Not reported<br>**<u>5.4. Patient satisfaction at 12 months post op</u>**<br>Patient satisfaction VAS ratings at 12 months follow-up:<br>I: 8.6 (SD ± 1.8)	

						C: 8.7 (SD ± 1.9)<br>MD: -0.03, 95% CI -0.7 to 0.7.<br><br>**<u>6. Return to work/sport</u>**<br>Not reported<br>**<u>6.1. Return to work/sport at 6 weeks post op</u>**<br>Not reported<br>**<u>6.2. Return to work/sport at 3 months post op</u>**<br>Not reported<br>**<u>6.3. Return to work/sport at 6 months post op</u>**<br>Not reported<br>**<u>6.4. Return to work/sport at 12 months post op</u>**<br>Not reported	
Zhang (2017)	<u>Type of study:</u><br>Clinical study<br><br><u>Setting and country:</u><br>132 cases were recruited from January 2013 to July 2016. Further not specified: however author was affiliated with the department of orthopedics, in Shanxi province, China.<br><br><u>Funding and conflicts of interest:</u><br>not reported	<u>Inclusion criteria</u>:<br>-restricted mobility of shoulder joint was no less than in 30 degrees at least 2 directions out of a total of 3 directions (anteflexion, abduction and external rotation); rotator cuff injury diagnosed by MRI; patients were willing to receive rehabilitation, and have good cooperation in evaluation.<br><br><u>Exclusion criteria</u>:<br>Patients with acute phase of shoulder injury, and the age is older than 70 years (> 70 years); patients with neurological disorders of the cervical vertebra of upper limbs; patients with dislocation of shoulder joint and previous surgical history, patients combined with severe cardiovascular,	Early motion group.<br>Patients included after undergoing an arthroscopic rotator cuff repair.<br><br>After surgery, the affected limbs were immobilized and suspended by using brackets in the two groups to keep the shoulder joint at 30 degrees abduction and 0 degrees external rotation. The patients in the observation group began exercise at 24 h postoperative and the motion range was gradually increased, active external rotation and back extensor exercise began at 72 h postoperative, strength training of the deltoid started 1 w postoperatively and muscular counterforce training started 6 w after surgery.<br>.	Control group received immobilization. Patients included after undergoing an arthroscopic rotator cuff repair.<br><br>Patients in the control group began exercise at 6 w after surgery.<br><br>The affected limbs were immobilized in the control group until 6 w after surgery, and the motion range of the shoulder joint was gradually increased.	<u>Length of follow-up</u>:<br>1 year follow-up<br><br><u>Loss-to-follow-up</u>: 0<br>Intervention:<br>Control:<br><br><u>Incomplete outcome data</u>: 0<br>Intervention:<br>Control:	**<u>1. Function (including post-op stiffness)</u>**<br>**<u>1.1. Function at 6 weeks post op</u>**<br>Not reported<br>**<u>1.2. Function at 3 months post op</u>**<br>Not reported<br>**<u>1.3. Function at 6 months post op</u>**<br>Not reported<br>**<u>1.4. Function at 12 months post op</u>**<br>*Constant shoulder score at 12 months postop*<br>I: 91.25 ± 10.93<br>C: 88.40 ± 11.37<br>MD: 2.85, 95% CI -0.95 to 6.65<br><br>**<u>2. Pain</u>**<br>Not reported<br>**<u>2.1. Pain at 6 weeks post op</u>**<br>Not reported<br>**<u>2.2. Pain at 3 months post op</u>**<br>Not reported	<u>Authors' conclusion:</u><br><br>Compared with immobilization, early motion can obtain similar functional outcomes in the later stage and reduce incidence of stiffness, which should be recommended in large size rotator cuff tear after arthroscopy repair.

Formaterat: Avstånd Efter: 0 pt, Radavstånd: enkelt

		cerebrovascular diseases, nervous system diseases and infection.<br><br>N total at baseline: 132<br>Intervention: 66<br>Control: 66<br><br>Important prognostic factors:<br><br>*Age (year)*<br>I: 52.32 ± 12.71<br>C: 50.43 ± 10.92<br><br>*Gender (male/female)*<br>I: 37/29<br>C: 32/34<br><br>*Tear length (mm)*<br>I: 37.28 ± 2.28<br>C: 38.49 ± 3.38<br><br>Groups comparable at baseline? Yes				**2.3. Pain at 6 months post op**<br>Not reported<br>**2.4. Pain at 12 months post op**<br>VAS score:<br>I: 3.27 ± 1.02<br>C: 3.51 ± 0.92<br>MD: -0.24, 95% CI -0.57 to 0.09<br><br>**3. Frozen shoulder**<br>Not reported<br>**3.1. Frozen shoulder 6 weeks post op**<br>Not reported<br>**3.2. Frozen shoulder 3 months post op**<br>Not reported<br>**3.3. Frozen shoulder 6 months post op**<br>Not reported<br>**3.4. Frozen shoulder 12 months post op**<br>*Ankylosis*<br>I: 10 (15.15)<br>C: 24 (36.36)<br>RD: -0.21, 95% CI -0.35 to -0.06; and RR: 0.41, 95% CI 0.21 to 0.80<br><br>**4. Complications/adverse events**<br>**4.1. Complications at 6 weeks post op**<br>Not reported<br>**4.2. Complications at 3 months post op**<br>Not reported<br>**4.3. Complications at 6 months post op**<br>Not reported<br>**4.4. Complications at 12 months post op**<br>*Incidence of complications: Re-tear*	

						(N, %) (time period not specified)<br>I: 6 (9.09)<br>C: 4 (6.06)<br>RD: 0.03, 95% CI -0.05 to 0.12; and RR: 1.5, 95% CI 0.44 to 5.07<br><br>**5. Patient satisfaction**<br>Not reported<br>**5.1. Patient satisfaction at 6 weeks post op**<br>Not reported<br>**5.2. Patient satisfaction at 3 months post op**<br>Not reported<br>**5.3. Patient satisfaction at 6 months post op**<br>Not reported<br>**5.4. Patient satisfaction at 12 months post op**<br>Not reported<br><br>**6. Return to work/sport**<br>Not reported<br>**6.1. Return to work/sport at 6 weeks post op**<br>Not reported<br>**6.2. Return to work/sport at 3 months post op**<br>Not reported<br>**6.3. Return to work/sport at 6 months post op**<br>Not reported<br>**6.4. Return to work/sport at 12 months post op**<br>Not reported	

Module 5.5

What is the effectiveness of surgical calcium removal compared to barbotage on patient-reported outcome measures in adult patients with supra- or infraspinatus calcarea tendinosis?

Patients	Patients with a calcareous tendinosis of the supra- or infraspinatus
Intervention	Surgical removal of limescale
Control	Barbotage
Outcomes	Pain, complications/adverse events, PROMs for function (CMS, DASH, WORC, ASES, OSS, DSST), patient satisfaction, return to work or leisure

Embase.com

**No.**	**Query**	**Results**
#11	#9 OR #10	88
#10	#6 AND #8 NOT #9 = RCT	62
#9	#6 AND #7 = SR	26
#8	'clinical trial'/exp OR 'randomization'/exp OR 'single blind procedure'/exp OR 'double blind procedure'/exp OR 'crossover procedure'/exp OR 'placebo'/exp OR 'prospective study'/exp OR rct:ab,ti OR random*:ab,ti OR 'single blind':ab,ti OR 'randomised controlled trial':ab,ti OR 'randomized controlled trial'/exp OR placebo*:ab,ti	3853133
#7	'meta analysis'/exp OR 'meta analysis (topic)'/exp OR metaanaly*:ti,ab OR 'meta analy*':ti,ab OR metanaly*:ti,ab OR 'systematic review'/de OR 'cochrane database of systematic reviews'/jt OR prisma:ti,ab OR prospero:ti,ab OR (((systemati* OR scoping OR umbrella OR 'structured	953361

Formaterat: Avstånd Efter: 0 pt, Radavstånd: enkelt

Formaterat: Avstånd Efter: 0 pt, Radavstånd: enkelt

Formaterat: Avstånd Efter: 0 pt, Radavstånd: enkelt

Formaterat: Avstånd Efter: 0 pt, Radavstånd: enkelt

Formaterat: Avstånd Efter: 0 pt, Radavstånd: enkelt

Formaterat: Avstånd Efter: 0 pt, Radavstånd: enkelt

	literature') NEAR/3 (review* OR overview*)):ti,ab) OR ((systemic* NEAR/1 review*):ti,ab) OR (((systemati* OR literature OR database* OR 'data base*') NEAR/10 search*):ti,ab) OR (((structured OR comprehensive* OR systemic*) NEAR/3 search*):ti,ab) OR (((literature NEAR/3 review*):ti,ab) AND (search*:ti,ab OR database*:ti,ab OR 'data base*':ti,ab)) OR (('data extraction':ti,ab OR 'data source*':ti,ab) AND 'study selection':ti,ab) OR ('search strategy':ti,ab AND 'selection criteria':ti,ab) OR ('data source*':ti,ab AND 'data synthesis':ti,ab) OR medline:ab OR pubmed:ab OR embase:ab OR cochrane:ab OR (((critical OR rapid) NEAR/2 (review* OR overview* OR synthes*)):ti) OR ((((critical* OR rapid*) NEAR/3 (review* OR overview* OR synthes*)):ab) AND (search*:ab OR database*:ab OR 'data base*':ab)) OR metasynthes*:ti,ab OR 'meta synthes*':ti,ab	
#6	#5 AND [2008-2023]/py	298
#5	#4 NOT ('conference abstract'/it OR 'editorial'/it OR 'letter'/it OR 'note'/it) NOT (('animal'/exp OR 'animal experiment'/exp OR 'animal model'/exp OR 'nonhuman'/exp) NOT ('human'/exp OR 'groups by age and sex'/exp))	500
#4	#1 AND (#2 OR #3)	616
#3	'barbotage'/exp OR 'lavage'/de OR 'guided needle'/exp OR barbotage*:ti,ab,kw OR (((needle* OR needling OR 'ultrasound guided' OR 'us guided' OR 'image guided') NEAR/3 (aspiration OR lavage OR fragmentation OR irrigation OR punctur* OR treatment* OR therap* OR procedure*)):ti,ab,kw)	93451
#2	'arthroscopy'/de OR 'arthroscopic surgery'/exp OR 'shoulder arthroscopy'/exp OR 'arthroendoscop*':ti,ab,kw OR 'arthroscop*':ti,ab,kw OR 'debridement'/exp OR 'debridement':ti,ab,kw OR 'surgery'/exp OR 'surgical patient'/exp OR 'surgical risk'/exp OR 'perioperative period'/exp OR surgic*:ti,ab,kw OR surger*:ti,ab,kw OR operation*:ti,ab,kw OR operative:ti,ab,kw OR presurg*:ti,ab,kw OR preoperati*:ti,ab,kw OR perisurg*:ti,ab,kw OR perioperati*:ti,ab,kw OR postsurg*:ti,ab,kw OR postoperati*:ti,ab,kw OR laparoscop*:ti,ab,kw OR intraoperati*:ti,ab,kw	7420378

Formaterat: Avstånd Efter: 0 pt, Radavstånd: enkelt

Formaterat: Avstånd Efter: 0 pt, Radavstånd: enkelt

Formaterat: Avstånd Efter: 0 pt, Radavstånd: enkelt

Formaterat: Avstånd Efter: 0 pt, Radavstånd: enkelt

Formaterat: Avstånd Efter: 0 pt, Radavstånd: enkelt

#1	('shoulder impingement syndrome'/exp/mj OR 'shoulder impingement syndrome*':ti,ab,kw OR 'subacromial impingement syndrome*':ti,ab,kw OR (('bursitis'/exp/mj OR bursitis:ti,ab,kw OR 'tendinitis'/exp/mj OR tendinitis:ti,ab,kw OR tendinosis:ti,ab,kw OR tendinopath*:ti,ab,kw OR 'impingement':ti,ab,kw) AND ('shoulder'/exp/mj OR 'rotator cuff':ti,ab,kw OR acromion:ti,ab,kw OR acromial:ti,ab,kw OR subacromial:ti,ab,kw OR shoulder*:ti,ab,kw)) OR 'rotator cuff injury'/exp/mj OR 'rotator cuff rupture'/exp/mj OR ((('rotator cuff' OR 'shoulder cuff' OR infraspinatus OR supraspinatus OR subscapularis) NEAR/3 (tear* OR lesion* OR rupture* OR tendinopath* OR laceration*)):ti,ab,kw) OR (('full-thickness':ti,ab,kw OR partial:ti,ab,kw) AND 'rotator cuff':ti,ab,kw) OR ('rotator cuff'/exp/mj AND ('rupture'/exp/mj OR 'injury'/mj OR 'laceration'/exp/mj))) AND ('calcification'/de OR 'calcinosis'/exp OR calcifying:ti,ab,kw OR calcification:ti,ab,kw OR calcarea*:ti,ab,kw OR calcific:ti,ab,kw OR calcinosis:ti,ab,kw OR calcified:ti,ab,kw OR calcinotic:ti,ab,kw)	1183

Ovid/Medline

#	Searches	Results
11	9 or 10	72
10	(6 and 8) not 9 = RCT	46
9	6 and 7 = SR	26
8	exp clinical trial/ or randomized controlled trial/ or exp clinical trials as topic/ or randomized controlled trials as topic/ or Random Allocation/ or Double-Blind Method/ or Single-Blind Method/ or (clinical trial, phase i or clinical trial, phase ii or clinical trial, phase iii or clinical trial, phase iv or controlled clinical trial or randomized controlled trial or multicenter study or clinical trial).pt. or random*.ti,ab. or (clinic* adj trial*).tw. or ((singl* or doubl* or treb* or tripl*) adj (blind$3 or mask$3)).tw. or Placebos/ or placebo*.tw.	2621313

Formaterat: Avstånd Efter: 0 pt, Radavstånd: enkelt

Formaterat: Avstånd Efter: 0 pt, Radavstånd: enkelt

Formaterat: Avstånd Efter: 0 pt, Radavstånd: enkelt

Formaterat: Avstånd Efter: 0 pt, Radavstånd: enkelt

Formaterat: Avstånd Efter: 0 pt, Radavstånd: enkelt

Formaterat: Avstånd Efter: 0 pt, Radavstånd: enkelt

7	meta-analysis/ or meta-analysis as topic/ or (metaanaly* or meta-analy* or metanaly*).ti,ab,kf. or systematic review/ or cochrane.jw. or (prisma or prospero).ti,ab,kf. or ((systemati* or scoping or umbrella or "structured literature") adj3 (review* or overview*)).ti,ab,kf. or (systemic* adj1 review*).ti,ab,kf. or ((systemati* or literature or database* or data-base*) adj10 search*).ti,ab,kf. or ((structured or comprehensive* or systemic*) adj3 search*).ti,ab,kf. or ((literature adj3 review*) and (search* or database* or data-base*)).ti,ab,kf. or (("data extraction" or "data source*") and "study selection").ti,ab,kf. or ("search strategy" and "selection criteria").ti,ab,kf. or ("data source*" and "data synthesis").ti,ab,kf. or (medline or pubmed or embase or cochrane).ab. or ((critical or rapid) adj2 (review* or overview* or synthes*)).ti. or (((critical* or rapid*) adj3 (review* or overview* or synthes*)) and (search* or database* or data-base*)).ab. or (metasynthes* or meta-synthes*).ti,ab,kf.	687043
6	limit 5 to yr="2008 -Current"	291
5	4 not (comment/ or editorial/ or letter/) not ((exp animals/ or exp models, animal/) not (humans/ or exp Age Groups/))	509
4	1 and (2 or 3)	536
3	exp Ultrasonography, Interventional/ or Therapeutic Irrigation/ or barbotage*.ti,ab,kf. or ((needle* or needling or 'ultrasound guided' or 'us guided' or 'image guided') adj3 (aspiration or lavage or fragmentation or irrigation or punctur* or treatment* or therap* or procedure*)).ti,ab,kf.	96650
2	exp Arthroscopy/ or 'arthroendoscop*'.ti,ab,kf. or 'arthroscop*'.ti,ab,kf. or exp Debridement/ or 'debridement'.ti,ab,kf. or exp Surgical Procedures, Operative/ or exp Specialties, Surgical/ or exp Perioperative Period/ or surgic*.ti,ab,kf. or surger*.ti,ab,kf. or operation*.ti,ab,kf. or operative.ti,ab,kf. or presurg*.ti,ab,kf. or preoperati*.ti,ab,kf. or perisurg*.ti,ab,kf. or perioperati*.ti,ab,kf. or postsurg*.ti,ab,kf. or postoperati*.ti,ab,kf. or laparoscop*.ti,ab,kf. or intraoperati*.ti,ab,kf.	5280298
1	(Shoulder Impingement Syndrome/ or 'shoulder impingement syndrome*'.ti,ab,kf. or 'subacromial impingement syndrome*'.ti,ab,kf. or ((exp bursitis/ or exp Tendinopathy/ or bursitis.ti,ab,kf. or tendinitis.ti,ab,kf. or tendinosis.ti,ab,kf. or tendinopath*.ti,ab,kf. or	1016

Formaterat: Avstånd Efter: 0 pt, Radavstånd: enkelt

Formaterat: Avstånd Efter: 0 pt, Radavstånd: enkelt

Formaterat: Avstånd Efter: 0 pt, Radavstånd: enkelt

Formaterat: Avstånd Efter: 0 pt, Radavstånd: enkelt

Formaterat: Avstånd Efter: 0 pt, Radavstånd: enkelt

Formaterat: Avstånd Efter: 0 pt, Radavstånd: enkelt

Formaterat: Avstånd Efter: 0 pt, Radavstånd: enkelt

	'impingement'.ti,ab,kf.) and (exp Shoulder/ or 'rotator cuff'.ti,ab,kf. or acromion.ti,ab,kf. or acromial.ti,ab,kf. or subacromial.ti,ab,kf. or shoulder*.ti,ab,kf.)) or Rotator Cuff/ or exp Rotator Cuff Injuries/ or (('rotator cuff' or 'shoulder cuff' or infraspinatus or supraspinatus or subscapularis) adj3 (tear* or lesion* or rupture* or tendinopath* or laceration*)).ti,ab,kf. or (('full-thickness' or partial) and 'rotator cuff').ti,ab,kf.) and (exp Calcinosis/ or calcifying.ti,ab,kf. or calcification.ti,ab,kf. or calcarea*.ti,ab,kf. or calcific.ti,ab,kf. or calcinosis.ti,ab,kf. or calcified.ti,ab,kf. or calcinotic.ti,ab,kf.)	

Evidence table module 5.5

Study reference	Study characteristics	Patient characteristics [2]	Intervention (I)	Comparison / control (C) [3]	Follow-up	Outcome measures and effect size [4]	Comments
Maugars (2009)	Type of study:<br>A randomized controlled study<br><br>Setting and country:<br>Patients were recruited between 1996 and 2001 in the departments of Rheumatology and Orthopaedics of the Nantes University Hospital.<br><br>Funding and conflicts of interest:	Inclusion criteria:<br>-chronic painful shoulders related to one or more tendon calcification of the cuff, symptomatic for more than 4 months, permanent or intermittent but with a continuation, despite usual medical symptomatic treatments, including analgesics, NSAID and infiltration.<br>-The length of the calcifications in their larger axis was more than 5 mm; localization of the calcification was in the infra or supra-spinatus, -the calcification of type A or	Describe intervention (treatment/procedure/test):<br><br>Bursoscopy (occurs under anaesthesia)<br><br>Almost all the patients without contraindication received a treatment with analgesics, NSAID and blinded corticosteroid infiltration before the protocol. To standardize a same medical approach of these patients, another corticosteroid infiltration (3.75 mg of cortivazol) was performed exactly at the proximity of the calcification under fluoroscopy. They were re-examined thereafter 2 weeks later. In the absence of a subjective global improvement higher than 70% (scale 0-100%) evaluated by the	Describe control (treatment/procedure/test):<br><br>Needling fragmentation irrigation (NFI)<br><br>Almost all the patients without contraindication received a treatment with analgesics, NSAID and blinded corticosteroid infiltration before the protocol. To standardize a same medical approach of these patients, another corticosteroid infiltration (3.75 mg of cortivazol) was performed exactly at the proximity of the calcification under fluoroscopy. They were re-examined thereafter 2 weeks later. In the absence of a subjective global improvement higher than 70% (scale 0-100%) evaluated by the	Length of follow-up:<br>T 1-4-12-24 months FU<br><br>Loss-to-follow-up:<br>Intervention:<br>Needling fragmentation irrigation<br>N (%) 3<br>Reasons (describe)<br>Lost of sight between T12 and T24 months (reason not described)<br><br>Intervention:<br>barbotage	**Outcome measures and effect size (include 95%CI and p-value if available):**<br><br>**T 12 months**<br>**1. Pain**<br>*Pain VAS improvement (%):*<br>I (BS): -26.7 ± 62.1<br>C (NFI): -56.1 ± 51.7<br>*Function VAS improvement (%):*<br>I: -27.9 ± 65.9<br>C: -50.1 ± 57.0<br>**2.Complications/adverse events**<br>Not reported<br>**3. PROMs for function:**<br>**3.1. CMS**<br>*Constant score improvement (%)*<br>I: +10.7 ± 29.2<br>C: + 20.7 ± 33.0<br>**3.2. DASH**	Conclusion: NFI and BS are validated techniques when other medical treatments have failed. Results were maintained after 24 months, and were similar between NFI and BS. However, NFI could e preferred because of its simplicity and costs (low).<br><br>N.B. the 49 other shoulders (54 patients) which had not been randomized and which had

Formaterat: Avstånd Efter: 0 pt

Formaterat: Avstånd Efter: 0 pt, Radavstånd: enkelt

Formaterad tabell

Formaterat: Avstånd Efter: 0 pt, Radavstånd: enkelt

Formaterat: Avstånd Efter: 0 pt, Radavstånd: enkelt

	Authors declare no conflict of interest.<br><br>Funding not reported	B was in accordance with the classification of the French Arthroscopic Society: type A: well-defined limits, dense and homogenous; type B: well-defined limits and fragmented.<br><br><u>Exclusion criteria</u>:<br>-excluded heterogeneous calcification with poorly defined limits (type C), often secondary to hyperalgic acute episodes with resorption of the calcification. -anticoagulant treatment, -haemorrhagiparous diseases, -recent acute hyperalgic flare (<1 month), -capsulitis, -known or clinically significant tear of the rotator cuff, -previous history of shoulder arthritis (infection or metabolic or chronic inflammatory rheumatism of the concerned shoulder), -previous history of fracture, luxation, surgery or arthroscopy, extracorporeal shock wave therapy or needling of the concerned shoulder, -type 3 accromion (Aggressive) according to the classification of Bigliani, -pregnancy, -allergy to Lidocaine, -	patient, a randomization was carried out by drawing preestablished lots in 3 groups.<br><br>The bursoscopy (BS) was carried out under general anaesthesia by two experienced surgeons. Both articular and sub-acromial stages were performed. The calcification was extracted either with a curette or with a high pressure water jet when it was softened. There was no acromioplasty neither tendon suture nor total bursectomy. The patients were hospitalized 12-24 h, and a rehabilitation began in the next 24-48 h, passive then active. A prescription of NSAID and analgesics were systematically prescribed, to use if necessary. They were each given a medical certificate for a 2 week recovery period.	patient, a randomization was carried out by drawing preestablished lots in 3 groups.<br><br>Needling fragmentation irrigation (NFI) were performed by the same practitioner. The patients was lengthened in dorsal decubitus, a cushion under the concerned shoulder and the arm in rotation to release calcification under the fluoroscopy. The conditions of asepsis included mask, casaque, sterile gloves and fields. The skin was disinfected with an iodized solution. A local anaesthesia of 15-30 cc of lidocaine 1% was injected from the surface of the skin until the proximity of the calcification. The first stage was a needling under fluoroscopy. A first needle (18 Gauge, 50 mm) was inserted directly in line with the X-Ray by an anterior way until the center of the calcification. The depth was located rotating the arm of the fluoroscopy. A second needle was inserted by an external way, perpendicular to the X-ray, in the axis of calcification. Several tens of intra-calcic drillings were carried out via the needles for this second phase of fragmentation. When the needle no longer meets any more resistance and does not bring back any more calcic cores, this second stage of fragmentation is considered as finished. The third stage is irrigation. One hundred to 200 cc of physiological salt solution is first injected through a needle	<u>N (%)</u> 2<br><u>Reasons (describe)</u><br>Lost of sight between T12 and T24 months (reason not described)<br><br><u>Control:</u> 1<br><u>N (%)</u><br><u>Reasons (describe):</u><br>Stopped attending consultation between T12 and T24 months<br><br><u>Incomplete outcome data:</u><br>not reported	**3.3. WORC**<br>**3.4. ASES**<br>**3.5. OSS**<br>**3.6. DSST**<br>**4. Patient satisfaction**<br>not reported<br><br>**<u>T 24 months</u>**<br>**1. Pain**<br>*Pain VAS improvement (%):*<br>I: -54.7 ± 56.9<br>C: -64.2 ± 47.8<br>*Function VAS improvement (%):*<br>I: -56.1 ± 48.0<br>C: -64.3 ± 55.5<br>**2.Complications/adverse events**<br>*Partial rupture of rotator cuff (all superficial and at the supra-spinatus level)*<br>I: 1<br>C: 1<br>*Complete rupture:*<br>I: 0<br>C: 0<br>*Capsulitis after removal of the calcification*<br>I: 1<br>C: 1<br>*Bursitis*<br>n = 8 cases<br>*Tendinitis of supra and/or infra-spinatus*<br>n = 15 cases<br>N.B.: without any significant difference between the three groups (NFI, CT, and BS) > exact numbers per group remains unknown.<br>**3. PROMs for function:**<br>**3.1. CMS**<br>*Constant score improvement (%)*<br>I: + 46.5 ± 37.4	significantly improved by the initial infiltration under fluoroscopy also benefited from a follow-up over two years. Twelve patients were randomized a second time. At T4 months: 3 in the NFI group and 4 in the BS group; at T12 months 2 in the NFI group and 1 in the BS group, and at T12 months, 1 in the NFI group and 1 in the BS group.<br><br>In this study: three groups were distinguished: the third was a control group of patients whom could benefit from NSAID and analgesics on request. After randomization of the 53 shoulders, patients were again randomized at amongst others T1 and T4 (due to condition which has not improved by more than 70 per cent at T4 for instance). In the long term, there were only six patients in the control group, the

		general anaesthesia contraindication.<br><br>N total at baseline: 96 patients (102 shoulders), however eventually 53 shoulders were randomized (49 shoulders not randomized; since these were improved by more than 70% by the simple infiltration near the calcification area).<br><br>Intervention (BS): 20<br>Control (NFI): 16<br><br>Important prognostic factors[2]:<br>*For example*<br>*age ± SD:*<br>*I (BS): 48.1*<br>*C (NFI): 46.8*<br><br>*Sex (F/M ratio):*<br>*I (BS): 12/5*<br>*C: 16/4*<br><br>*NSAID use*<br>*I: 94%*<br>*C: 90%*<br><br>Groups comparable at baseline? Yes		and then recovered by the other, making it possible to bring back calcic material and tissue fragments, sometimes more or less moderately haemorrhagic. Irrigation is considered as finished when the seringe brings back a clear liquid. Corticoid infiltration is not carried out at the end of the technique, so as not to interfere with the results of the two other groups on the one hand, and so as not to prevent the expected calcic resorption on the other hand. A control X-ray is taken at the end of the technique, showing most often a persisting calcification, which evolved heterogenous and hypodense.<br>A gleno-humeral arthrography was performed to make sure that there was no rupture of the rotator cuff caused by the technique. The patient was informed of a possible recrudescence of the pain in the 24-72 h following the technique, and an analgesic and NSAID treatment was systematically prescribed. Each patient was given a minimum of two weeks time off work. Physiotherapy was not systematically recommended.		C: + 25.9 ± 27.8<br>**3.2. DASH**<br>**3.3. WORC**<br>**3.4. ASES**<br>**3.5. OSS**<br>**3.6. DSST**<br>**4. Patient satisfaction**<br>not reported	other 11 patients required a therapeutic removal technique of the calcification during the 2 year follow-up.<br><br>**Outcomes (secondary outcomes of this study)**<br>Functional score of Constant out of 100; VAS of the pain; VAS of the functional repercussion; mobility of the shoulder; testing for sub-acromial conflict (Hawkins and Neer); testing of the infra and supra-spinatus tendons; intake of NSAID and analgesics; duration of time off work; area of calcifications

Formaterat: Avstånd Efter: 0 pt
